# Dairy Intake in Relation to Prediabetes and Continuous Glycemic Outcomes: A Systematic Review and Dose-Response Meta-Analysis of Prospective Cohort Studies

**DOI:** 10.1016/j.cdnut.2024.104470

**Published:** 2024-09-29

**Authors:** Isabel AL Slurink, Yakima D Vogtschmidt, Bo Brummel, Tom Smeets, Nina Kupper, Sabita S Soedamah-Muthu

**Affiliations:** 1 Center of Research on Psychological disorders and Somatic Diseases (CoRPS), Department of Medical and Clinical Psychology, Tilburg University, Tilburg, The Netherlands; 2 Hugh Sinclair Unit of Human Nutrition, Department of Food and Nutritional Sciences, University of Reading, Reading, United Kingdom; 3 Institute for Cardiovascular and Metabolic Research, University of Reading, Reading, United Kingdom; 4 Institute for Food, Nutrition and Health, University of Reading, Reading, United Kingdom

**Keywords:** dairy, milk, yogurt, cheese, dose-response associations, meta-analysis, observational studies, prediabetes, impaired fasting glucose, impaired glucose tolerance

## Abstract

**Background:**

Modest inverse associations have been found between dairy intake, particularly yogurt, and type 2 diabetes risk. Investigating associations of dairy intake with early onset of type 2 diabetes offers opportunities for effective prevention of this condition.

**Objectives:**

This study aims to investigate the relationships between the intake of different dairy types, prediabetes risk, and continuous glycemic outcomes.

**Methods:**

Systematic literature searches across multiple databases were performed of studies published up to September 2023. Included were prospective cohort studies in healthy adults that examined the association between dairy intake and prediabetes risk according to diagnostic criteria, or continuous glycemic markers. A dose-response random-effects meta-analysis was used to derive incremental relative risks (RRs) for associations of total dairy, fermented dairy, milk, yogurt, cheese (all total, high-fat, and low-fat), cream, and ice cream with prediabetes risk adjusted for sociodemographic, health and cardiometabolic risk factors, and dietary characteristics.

**Results:**

The meta-analyses encompassed 6653 prediabetes cases among 95,844 individuals (age range 45.5–65.5 y) including 6 articles describing 9 cohorts. A quadratic inverse association was observed for total dairy intake and prediabetes risk, with the lowest risk at 3.4 servings/d (RR: 0.75; 95% confidence interval: 0.60, 0.93; *I*^2^ = 18%). Similarly, total, and high-fat cheese exhibited nonlinear inverse associations with prediabetes risk, showing the lowest risk at 2.1 servings/d (0.86; 0.78, 0.94; *I*^2^ = 0%, and 0.90; 0.81, 0.99; *I*^2^ = 12%), but a higher risk at intakes exceeding 4 servings/d. Ice cream intake was linearly associated with prediabetes risk (0.85; 0.73, 0.99; *I*^2^ = 0% at the highest median intake of 0.23 servings/d). Other dairy types showed no statistically significant associations. The systematic review on dairy intake and glycemic outcomes showed considerable variabilities in design and results.

**Conclusions:**

The findings suggest an inverse association between moderate dairy and cheese intake in preventing prediabetes. The potential for reverse causation and residual confounding highlights the need for studies with comprehensive repeated measurements.

**Trial registration number:**

PROSPERO 2023 CRD42023431251.

## Introduction

Prediabetes is a high-risk stage for developing type 2 diabetes. People in this risk stage already display some insulin resistance and declined pancreatic beta-cell function, resulting in impaired fasting or postprandial glycaemia. The prevalence of prediabetes is increasing at an alarming rate due to the aging of populations, economic developments, and unhealthier lifestyles [1]. Thus, effective preventive strategies for prediabetes are crucially needed. Many dietary guidelines worldwide advise the consumption of 2–3 daily servings of dairy, based on systematic reviews showing evidence of a protective association between low-fat dairy and yogurt intake and type 2 diabetes [2,3].

The role of different dairy types in relation to prediabetes risk was investigated in detail in prospective cohorts in various Western world countries [[4], [5], [6], [7], [8], [9]]. By focusing on individuals without prediabetes at baseline, these cohort studies offer insights into the risk factors associated with the early onset of type 2 diabetes. This approach also eliminates a potential source of heterogeneity, as associations of risk factors with type 2 diabetes may vary depending on the level of glycemic disturbances at baseline [9]. Findings from these reports, however, show conflicting results [[4], [5], [6], [7], [8], [9]]. Discrepancies in the direction and strength of these associations across different cohorts may be attributed to variations in countries, study population characteristics, variety in dairy products, and corresponding intake ranges.

To comprehensively investigate the association between dairy consumption and glycemic control, studies incorporating continuous glycemic markers, including those for insulin sensitivity and resistance, are crucial. These studies may provide insights into distinct mechanistic aspects related to maintenance of normal glycemic control before the onset of disease. Moreover, continuous glycemic markers may provide a more sensitive assessment compared with binary outcomes such as prediabetes. However, interpreting related effect estimates across studies becomes challenging due to variations in baseline glycemic status. Additionally, as compared with risk estimates, effect estimates related to continuous glycemic markers might be more difficult to translate into actionable public health implications.

To draw robust conclusions about how the type and dosage of dairy products consumed relate to incident prediabetes and glycemic markers, a systematic review of the literature and meta-analysis of prospective cohort studies is needed. These types of extensive meta-analyses are considered at the top of the hierarchy of evidence [10]. Therefore, these results may further refine current scientific-based food-based dietary guidelines [11]. This study aims to conduct a systematic review and meta-analysis of prospective observational associations between intake of total and different types of dairy products, with different fat contents, and incident prediabetes and glycemic markers in healthy adult populations.

## Methods

The study protocol for this review can be found at https://www.crd.york.ac.uk/prospero/display_record.php?RecordID=431251. This review was performed following the PRISMA and Meta-analysis of Observational Studies in Epidemiology guidelines [12,13].

### Search strategy

Articles were retrieved from electronic databases including PubMed, Scopus, Web of Science, and the Cochrane Library. The search was confined to studies published from the initiation of research in the field up to 18 April, 2024. Searches were performed using key terms in the title/abstract of published studies and with Medical Subject Heading terms, where possible. Authors of relevant abstracts were contacted for potential inclusion of unpublished data. Grey literature was examined by inspecting the first 200 items of a Google Scholar search. The full search strategy can be found in the Supplemental Table 1.

### Study selection

**FIGURE 1:**
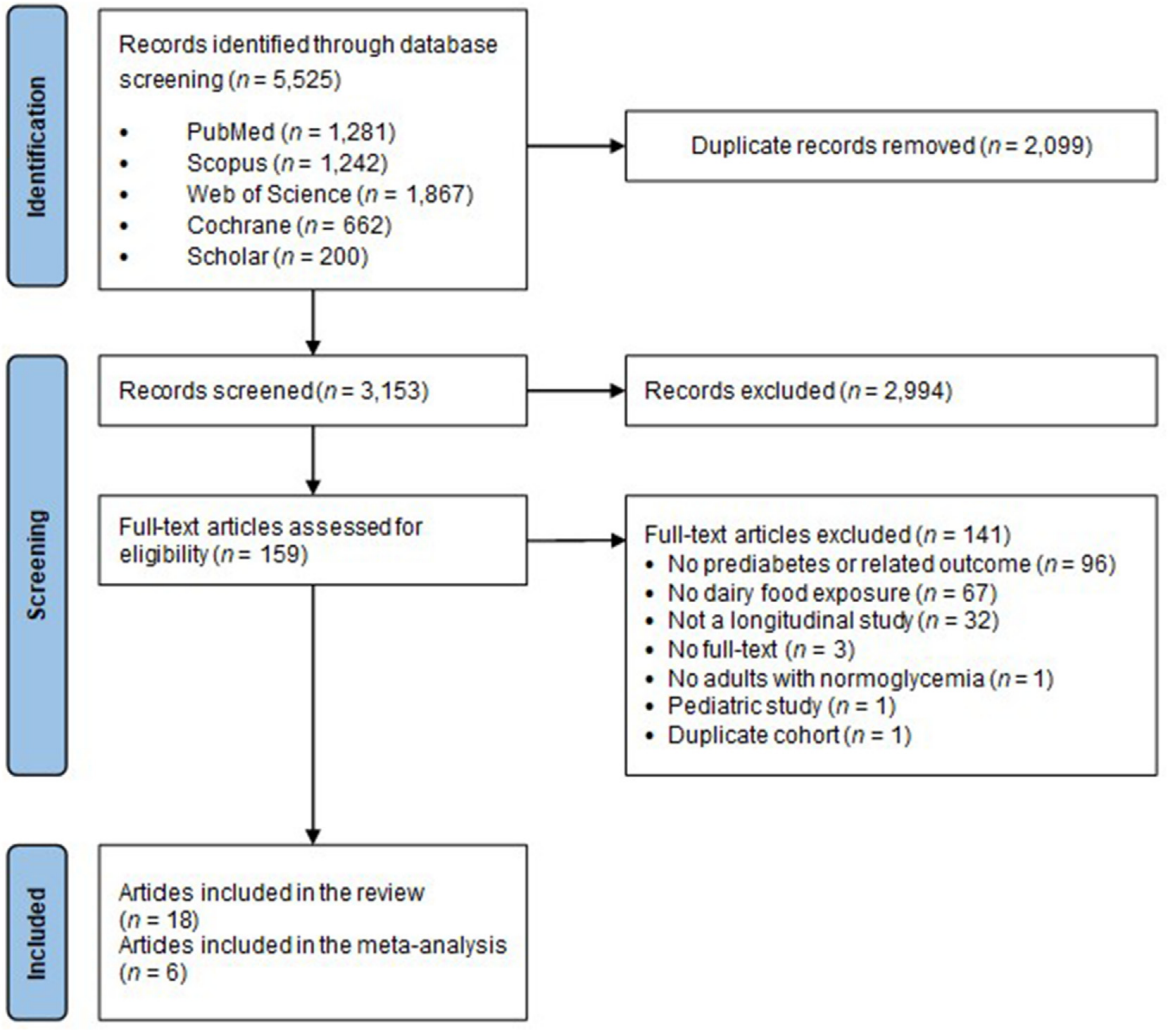
PRISMA flowchart for the systematic review detailing the database searches, the number of abstracts screened, and the full-text retrieved.

Articles were imported in Endnote and duplicates were removed based on article references. In a step-by-step process, 2 authors (IS and YDV) independently performed the title screening, abstract screening, and full-text screening according to predefined eligibility criteria in Rayyan [14]. Inclusion criteria were observational studies as design involving adult (>18 y) participants with normoglycemia as the study population, dairy food consumption as the main exposure of interest, and prediabetes or glycemic markers as the main study outcomes. Study designs may include prospective cohort studies, nested case-control studies, case-cohort studies, and observational follow-up studies of randomized controlled trials (RCTs). The articles had to be original research and written in English. For prediabetes (i.e., impaired fasting glucose or impaired glucose tolerance) the following recognized diagnostic criteria were used: fasting plasma glucose (FPG) between 110 and 125 mg/dL [15] or between 100 mg/dL and 125 mg/dL [16]; or hemoglobin A1c (HbA1c) levels between 6.0% and 6.4% [17] or 5.7% and 6.4% [16]; or 2-h plasma glucose (2hPG) based on an oral glucose tolerance test of ≥140 and <200 mg/dL [15,16]. Other glycemic outcomes included fasting (or random) plasma glucose, 2hPG, HbA1c, fasting serum insulin, HOMA-IR, insulin sensitivity index (Matsuda index), Stumvoll metabolic clearance rate, Stumvoll insulin sensitivity index, oral glucose insulin sensitivity index, Gutt index, quantitative insulin sensitivity check index, glucose-to-insulin ratio, other measures or indices of glucose or insulin sensitivity. We excluded studies conducted in animals, children, pregnant or lactating female, and ill populations (e.g., in patients with diabetes, cardiovascular disease, or cancer). Any disagreement was resolved until consensus was reached (IS and YDV). The reference lists of eligible articles and review articles were checked for additional eligible studies. Of the 159 fully reviewed articles, 18 met the inclusion criteria for a systematic review, of which 6 met the criteria for meta-analysis (Figure 1). Details on the reasons for the exclusion of fully reviewed articles are provided in Supplemental Table 2.

### Data extraction

Three authors (IS, YDV, and BB) independently extracted data from the full-text of eligible articles, according to a predefined protocol. The following data were extracted: bibliographic information; author(s); publication year; journal; title of the article; country; cohort study name; sample size; follow-up; participant characteristics; median or range of intake; number of subjects and prediabetes cases or mean outcome values; and confounder adjusted relative risks (RRs), odds ratios, hazard ratios or beta coefficients (*β*s) and their corresponding 95% confidence intervals (CI) or SEs. Effect estimates derived from multiple adjusted models in different studies, where similar confounders were considered, were pooled, allowing for insights into the importance of confounding. For studies not reporting the median of each category, the means of the lower and the upper limits were extracted. The meta-analyses for composite dairy types (total and fermented dairy) were conducted in servings/d. Definitions of the different types of dairy in each included article can be found in Supplemental Table 3. This approach was chosen as all studies uniformly defined a serving size by distinguishing liquid and solid dairy types (e.g., 200 g for liquid dairy foods and 20 g for solid dairy foods). The intake of different dairy types, presented in servings/d or wk, was converted into g/d using either the reported conversion units in the article or country-specific standard units. For dairy types consisting of food items with different serving sizes, we averaged the serving sizes, which was the case for cheese in 1 article [9], and cream in 2 studies [8,9]. Subsequently, we conducted the meta-analyses, standardizing the measurements to servings/d, enforcing equal water content of each type: 150 g for milk, yogurt, and ice cream; and 20 g for cheese. An exception was made for cream because of low intakes, where we operationalized the serving size as 15 g.

### Risk of bias assessment

Three authors (IS, YDV, and BB) independently evaluated the risk of bias for the included studies using the Risk Of Bias In Non-randomized Studies – of Exposure (ROBINS-E) tool (for studies in which IS was involved as an author, only YDV and BB conducted the assessment) [18]. The tool evaluates 7 domains of bias: *1*) confounding, *2*) measurement of exposure, *3*) selection of participants into the study (or analysis), *4*) postexposure interventions, *5*) missing data, *6*) measurement of the outcome, and *7*) selection of the reported result. Each domain, as well as the overall risk of bias, was rated as either low risk, some concerns, high risk, or very high risk of bias. Further details on the ROBINS-E assessment are provided in Supplemental Table 4.

### Data synthesis and analysis

Meta-analyses were performed when ≥3 cohorts per dairy type and outcome were available. Although this criterion was met for all dairy types regarding prediabetes, it was not fulfilled for the glycemic markers. Analyses were performed using the R packages dosresmeta, metafor, and rms in R version 4.1.2 (R Foundation for Statistical Computing) [[19], [20], [21]].

A 2-stage linear random-effects meta-analysis was performed to obtain a single RR of each study expressed per serving size. Forest plots were created displaying the effect size of each study, its precision, and its weight to the summary estimate (Supplemental Figures 1–17). Contour-enhanced funnel plots were used to investigate potential publication bias and small-study effects were tested using the Egger’s test [22]. Furthermore, small-study effects were ascertained using a DOI plot to visualize asymmetry and the Luis Furuya–Kanamori index (LFK) index to quantify asymmetry of study effects [23]. The LFK indexes indicated asymmetry as follows: <±1 no asymmetry, ±1–2 minor asymmetry, and >±2 major asymmetry.

Two-stage dose-response random-effects meta-analyses were used to derive incremental dose-response RRs [24]. Potential nonlinear associations were examined using quadratic and restricted cubic spline models. Likelihood ratio tests and information criteria (Akaike Information Criterion and the Bayesian Information Criterion) were used to determine the most appropriate model fit and knot points (Supplemental Table 5). Associations were visualized using spaghetti plots. In spaghetti plots, the pooled RR and 95% CI at each quantity of intake is plotted, as well as cohort-specific RRs with study-specific weights. To assess heterogeneity between studies, the heterogeneity statistic (*I*^2^) was calculated with the Higgins and Thompson method [25]. Cochrane’s *Q* test was conducted to evaluate if variation in effect estimates is likely due to chance alone. Four models with similar confounder adjustments in each study were compared; model 1 included age, sex, and energy intake, and model 2 additionally adjusted for an indicator of socioeconomic status (SES) such as educational level, smoking, alcohol use, physical activity level, family history of diabetes, model 3 additionally adjusted for food groups associated with type 2 diabetes including fruit, vegetables, bread, legumes, nuts, red and processed meat, coffee, tea, and sugar-sweetened beverages, and model 4 additionally adjusted for BMI (in kg/m^2^), waist circumference, and cardiometabolic risk factors (e.g., dyslipidemia, hypertension). The results of model 4 are presented in the main text.

We performed a sensitivity analysis, excluding 1 study at a time from the analyses. Furthermore, we repeated the meta-analyses using a fixed-effect model to evaluate the consistency of results assuming a single true effect size across all studies, showing no differences in effect estimates (Supplemental Table 6). A meta-regression was performed to explain heterogeneity. Potential moderators included follow-up duration, calendar year of dairy intake assessment, the prediabetes definition used, and the literature quality score. Moderation by age, sex, and BMI was not explored due to limited variation and a restricted number of cohorts. Moderation by geographical location and country-level SES was not feasible as all studies were conducted in Western, high-SES countries. Additionally, the dietary assessment method and quality score were not considered as moderators, as all studies uniformly utilized a food frequency questionnaire (FFQ) and were graded with similar quality. Each moderator was sequentially added as a covariate in the meta-regression model. The model fit of each bivariate model was compared using a deviance test and the adjusted *R*^2^, indicating the percentage of heterogeneity explained by the covariate.

### Certainty of evidence

The Grading of Recommendations Assessment, Development and Evaluation method was applied by 1 author (IS) to assess the certainty of the evidence (CoE) for associations between dairy exposures and prediabetes risk on a 4-point scale, ranging from “very low” to “high” [26,27]. A second author (YDV) reviewed the results. Observational studies start with a “low” certainty rating due to their inherent limitations, which may be adjusted based on specific criteria. Evidence quality may be downgraded for high risk of bias (indicated by the ROBINS-E [18]); inconsistency (substantial unexplained heterogeneity based on *I*^2^ > 50% and a *P* value for heterogeneity <0.10); indirectness (due to factors affecting generalizability including differences in populations, exposures, or outcomes); imprecision (95% CIs include the minimally important difference of 5%, RR: 0.95–1.05); or publication bias (significant evidence of small-study effects). Certainty of evidence can be upgraded for a large risk estimate (RR: <0.5 or >2), or a dose-response relationship.

## Results

### Dairy and prediabetes

An overview of the characteristics of 6 articles incorporating 9 study populations in prospective cohort designs can be found in Table 1. A total of 6653 prediabetes cases among 95,844 individuals were identified, with the mean age ranging from 45.5 to 65.5 y. The sample sizes ranged from 997 to 74,132, and the follow-up duration ranged from 4.1 to 20.9 y. All articles were based on Western samples; 3 in the Netherlands, 2 in the United Kingdom [8,9], and 1 in Australia [6]. Five articles were published by our research group in collaboration with principal investigators from the respective cohorts [[4], [5], [6], [7], [8]], and only 1 additional article was identified through the systematic review [9]. On the basis of population-level median intake quantities, total dairy intake ranged from 1.1 to 3.7 servings/d (Table 2). Among all dairy types, milk contributed most to the total dairy intake, with median intakes ranging from 0.6 to 1.5 serving/d (96.5–220 g/d) for low-fat milk and 0.03 to 1.3 servings/d (5.2–200 g/d) for high-fat milk. All studies were judged as having some concerns regarding the risk of bias (Supplemental Figure 18), primarily due to confounding in all studies, lack of repeated exposure measurements in 4 studies [4,[6], [7], [8]], and missing data from lost-to-follow-up in 3 studies [4,6,8] (Supplemental Figure 19A).

**TABLE 1:** Prospective cohort studies reporting associations between dairy product intake and prediabetes risk.

Author, year	Cohort (follow-up duration), baseline period, country	Female (%)	Mean age (y)	Mean BMI (kg/m^2^)	Dietary assessment	Dairy types^1^	Prediabetes assessment	Cases, *N*	Adjustments
Hruby et al. [9], 2017	Framingham Heart Study Offspring Cohort (10.5 y), 1991–1995, United States	57.0	52.6	26.4	126-item FFQ averaged across 4 examinations. Validated against dietary records, highest for yogurt, *r* = 0.94–0.97; lowest for cheese, *r* = 0.38–0.57	Dairy, milk (all total, high-fat and low-fat), yogurt, cheese and cream	FPG 100–126 mg/dL, first incident measurement	902/1867	Age, sex, energy intake, parental history of diabetes, smoking, dyslipidemia, hypertension, coffee, nuts, fruits, vegetables, meats, alcohol, fish, glycemic index, other dairy, baseline BMI and weight change over follow-up
Slurink et al. [4], 2022	Hoorn Study I (6 y), 1989–1992, The Netherlands	44.0	59.6	25.9	92-item FFQ. Validated against a dietary history, *r* = 0.72 for ranking of energy intake, .68 for animal protein, 0.73 for SFA, 0.69 for sodium, and 0.75 for calcium	Dairy, fermented dairy, milk and milk products, plain milk, yogurt, cheese (all total, high-fat and low-fat), cream and ice cream	FPG 110–125 mg/dL or 2hPG 140–199 mg/dL or HbA1c 42–46 mmol/mol	329/997	Age, sex, energy intake, follow-up duration, cohort, education, smoking, PA, alcohol intake, family history of diabetes, fruit, vegetables, tea, coffee, grains, meat, SSBs, BMI, LDL-c, and blood pressure
Slurink et al. [5], 2022	Hoorn Study II (6.7 y), 2006–2007, The Netherlands	55.0	53.0	25.6	104-item FFQ. Validated against actual energy intake in controlled feeding trials, *r* = 0.82 for energy intake, and validated against 3 24–h recalls, energy adjusted, deattenuated *r* = 0.60 for animal protein, 0.55 for SFA, 0.67 for calcium, 0.61 for cheese, and 0.75 for milk and milk products	—	—	482/1265	—
Slurink et al. [6], 2023	AusDiab (12 y), 1999–2000, Australia	56.7	49.0	26.1	74-item FFQ. Validation against 7-d weighted food records, energy adjusted *r* = 0.39 for protein, 0.64 for SFA, 0.59 for calcium, and 0.30 for sodium	Dairy, milk, cheese (all total, high-fat and low-fat), fermented dairy, yogurt, and ice cream	FPG 110–125 mg/dL or 2hPG 140–199 mg/dL	765/4891	Age, sex, energy intake, education, smoking, PA, alcohol intake, family history of diabetes, fruit, vegetables, grains, legumes, nuts, red and processed meat, fruit juice, WC, change in WC, LDL-c, and hypertension
Slurink et al. [5], 2022	Rotterdam Study, I (20.9 y), 1989–1993, The Netherlands	59.6	65.5	26.0	170-item FFQ. Validated against 15 24-h recalls; energy adjusted, deattenuated *r* = .66 for protein, 0.52 for SFA, 0.72 for calcium, 0.58 for sodium, and against 24h urine collection; 0.67 for protein	Dairy, fermented dairy, milk and milk products, plain milk, yogurt, cheese (all total, high-fat and low-fat), cream and ice cream	FPG 110–125 mmol/L or PG 140–199 mg/dL, first incident measurement	519/2617	Age, sex, energy intake, education, smoking, PA, alcohol intake, family history of diabetes (not in RS-III), fruit, vegetables, whole grains, legumes, nuts, tea, coffee, red meat and SSBs and longitudinal waist circumference
Slurink et al. [5], 2022	Rotterdam Study, II (12.6 y), 2000, The Netherlands	55.1	63.6	27.0	—	—	—	290/1250	—
Slurink et al. [5], 2022	Rotterdam Study, III (7 y), 2006, The Netherlands	59.9	56.8	27.2	389-item FFQ. Validated against 9-d dietary records and a 4-wk dietary history, *r* = 0.74 for energy, 0.61 for animal protein intake, 0.73–.75 for SFA, 0.60 for calcium, 0.60 for milk and milk products and 0.61 for cheese	—	—	330/2186	—
Slurink et al. [7], 2023	Lifelines Study (4.1 y), 2006–2013, The Netherlands	59.7	45.5	25.7	110-item FFQ (heart). The complete FFQ (heart + petals was compared with a conventional FFQ, *r* = 0.68 for energy, 0.61 for animal protein, 0.65 for SFA, 0.62 for calcium, 0.57 for cheese and 0.69 for dairy, and validated against urinary sodium (0.40) and potassium (0.37) excretions. 75% and 73% of participants, respectively, ranked in the same or adjacent quartile	Dairy, fermented dairy, plain milk, yogurt, cheese (all total, high-fat and low-fat), milk and milk products, cream and ice cream	FPG 110–125 mg/dL or HbA1c 42–46 mmol/mol	2746/74,132	Age, sex, energy intake, follow-up duration, educational level, alcohol use, smoking, PA, family history of diabetes, fruit, vegetables, bread, legumes, nuts, red and processed meat, coffee, tea, SSBs, TAGs, LDL-c, waist circumference, and hypertension
Slurink et al. [8], 2024	Fenland Study (6.7 y), 2005–2015, United Kingdom	51.9	48.7	26.4	130-item FFQ. Validated against 16-d weighted records, *r* = 0.52 for energy, 0.43, for protein, 0.56 for and SFA, 0.50 for calcium, and against 7-d food diaries, 0.56 for milk, 0.57 for yogurt and 0.33 for cheese	Dairy, fermented dairy, plain milk, yogurt, cheese (all total, high-fat and low-fat), cream and ice cream	FPG 110–125 mg/dL, 2hPG 140–199 mg/dL or HbA1c 42–46 mmol/mol	290/6639	Age, sex, study site, energy intake, educational level, age at completion of education, ethnic origin, alcohol use, smoking, PA, family history, fruit, vegetables, whole grains, refined grains, potatoes, legumes, nuts, red and processed meat, fatty fish, coffee, tea, SSBs, hypertension, dyslipidemia, and waist circumference

Abbreviations: 2hPG, 2-h postprandial glucose; FFQ, food frequency questionnaire; FPG, fasting plasma glucose; HbA1c, hemoglobin A1c; PA, physical activity; RS, Rotterdam Study; SSB, sugar-sweetened beverage; TAG, triacylglycerol; WC, waist circumference.

1 The dairy types were defined into uniform categories by the authors. The original definitions are found in Supplemental Table 3.

**TABLE 2:** Characteristics and outcomes of separate 2-stage random-effects dose-response meta-analyses, per dairy exposure.

Exposure	No. of cohorts (articles)	Total *N*	No. of cases	Mean follow-up (y)	Range median intake^1^ (servings/d)	Model fit (*P*_nonlinearity_)	RR (95% CI) at 1 serving/d^2^	Lowest or highest RR of quadratic fit (95% CI)^2^	Heterogeneity			Certainty of evidence
									*I*^2^	*Q* test	*P* value	
Total dairy	9 (6)	95,844	6653	9.6	1.1–3.7	Quadratic (*P* < 0.0001)	0.87 (0.78, 0.96)	0.75 (0.60, 0.93) at 3.4 servings/d	18%	19.5	0.24	Low
High-fat dairy	9 (6)	95,844	6653	9.6	0.3–2.0	Linear	0.99 (0.96, 1.02)		37%	12.7	0.12	Low
Low-fat dairy	9 (6)	95,844	6653	9.6	0.8–2.6	Quadratic (*P* < 0.0001)	1.00 (0.95, 1.05)	0.93 (0.66, 1.32) at 5.2 servings/d	28%	22.1	0.14	Very low
Fermented dairy	8 (5)	93,975	5751	9.5	0.7–2.4	Quadratic (*P* < 0.0001)	0.93 (0.86, 1.01)	0.91 (0.81, 1.02) at 2 servings/d	0%	9.4	0.81	Low
High-fat fermented dairy	7 (4)	89,089	4986	9.1	0.8–1.7	Quadratic (*P* = 0.001)	0.95 (0.88, 1.02)	0.94 (0.86, 1.03) at 1.7 servings/d	0%	7.9	0.79	Low
Low-fat fermented dairy	7 (4)	89,089	4986	9.1	0.2–0.7	Linear	0.98 (0.94, 1.03)		0%	1.8	0.94	Low
Total milk	9 (6)	95,844	6653	9.6	0.9–2.4	Quadratic (*P* < 0.0001)	0.98 (0.88, 1.08)	0.96 (0.84, 1.09) at 3.5 servings/d	37%	25.3	0.07	Very low
High-fat milk	9 (6)	95,844	6653	9.6	0.03–1.3	Linear	0.97 (0.88, 1.08)		59%	19.3	0.01	Very low
Low-fat milk	9 (6)	95,844	6653	9.6	0.6–1.5	Quadratic (*P* < 0.0001)	1.03 (0.95, 1.12)	0.87 (0.73, 1.04) at 4.1 servings/d	30%	22.8	0.12	Low
Total yogurt	9 (6)	95,844	6653	9.6	0.02–0.5	Linear	0.98 (0.90, 1.07)		8%	8.7	0.37	Very low
High-fat yogurt	7 (4)	89,086	4986	9.6	0.2–0.4	Linear	1.04 (0.87, 1.25)		21%	7.6	0.27	Very low
Low-fat yogurt	7 (4)	89,086	4986	9.6	0.09–0.4	Linear	0.97 (0.82, 1.15)		0%	2.9	0.83	Very low
Total cheese	9 (6)	95,844	6653	9.6	0.6–2.9	Quadratic (*P* < 0.0001)	0.89 (0.84, 0.95)	0.86 (0.78, 0.94) at 2.1 servings/d	0%	12.6	0.70	Low
High-fat cheese	8 (5)	93,977	5751	9.5	0.4–2.4	Quadratic (*P* < 0.0001)	0.92 (0.87, 0.98)	0.90 (0.81, 0.99) at 2.1 servings/d	12%	15.8	0.32	Low
Low-fat cheese	8 (5)	93,977	5751	9.5	0.2–1.0	Linear	1.05 (0.98, 1.14)		48%	13.5	0.06	Very low
Cream	8 (5)	90,953	5888	9,3	0.02–0.8	Linear	0.85 (0.69, 1.05)		0%	6.2	0.51	Very low
Ice cream	8 (5)	96,239	6562	9.5	0.02–0.06	Linear	0.50 (0.26, 0.94)		0%	2.4	0.93	Low

Abbreviations: CI, confidence interval; RR, relative risk.

1 For composite dairy types, serving sizes were 200 g for liquid dairy foods and 20 g for solid dairy foods. For individual dairy types, serving sizes were 150 g for milk, yogurt, and ice cream; 20 g for cheese, and 15 g for cream.

2 The relative risks (95% CIs) were adjusted for age, sex, energy intake, educational level, smoking behavior, physical activity, alcohol intake, family history of diabetes, intake of food groups, waist circumference or BMI, hypertension, and dyslipidemia.

#### Total, low-fat, and high-fat dairy and prediabetes

**FIGURE 2:**
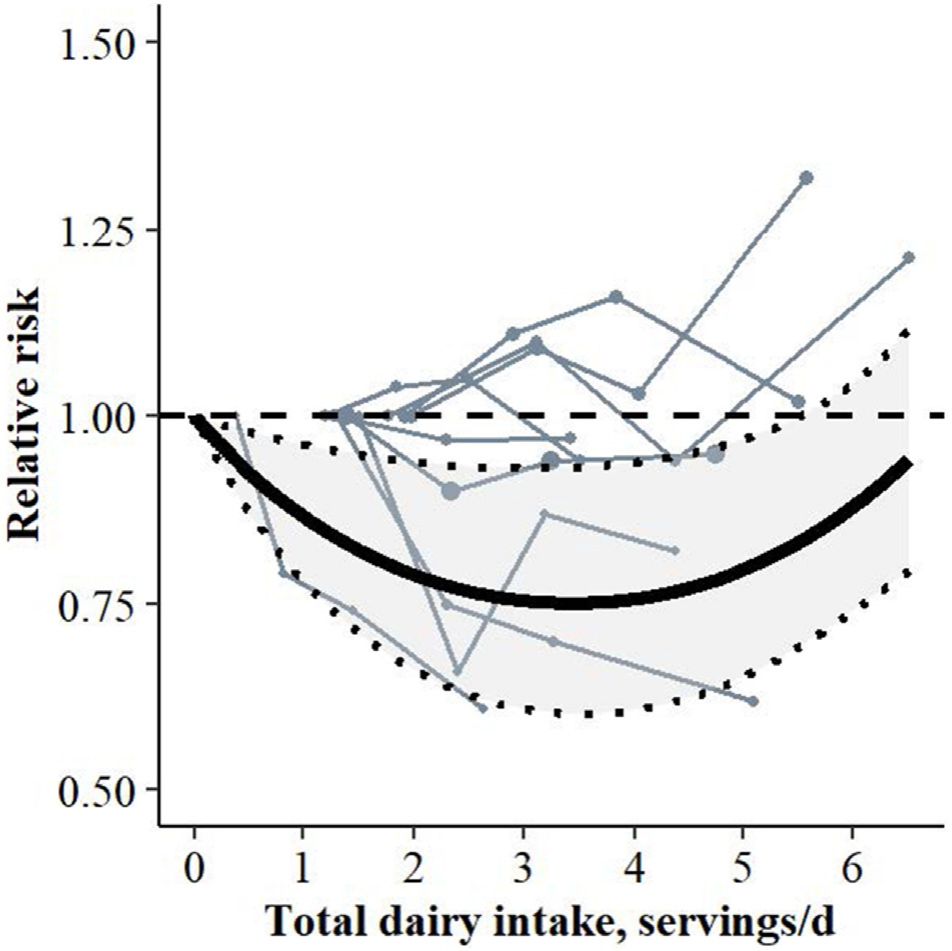
Spaghetti plot based on dose-response meta-analysis including 6 studies and 9 cohorts (6653 cases among 95,844 participants) for the association between total dairy and prediabetes risk (lowest RR at 3.4 servings/d: 0.75, 95% CI: 0.60, 0.93, *I*^2^ = 18%). The solid black line represents the pooled RR at each quantity of intake. The light gray colored area between the dotted black lines indicates the 95% CI. The dashed gray line at RR = 1.00 represents the reference line. Each solid gray line represents a cohort with circles placed at the cohort-specific RRs at the corresponding intake level. The area of the circle is proportional to the study-specific weight. The associations were adjusted for age, sex, energy intake, educational level, smoking behavior, physical activity, alcohol intake, family history of diabetes, intake of food groups, waist circumference or BMI, hypertension, and dyslipidemia. Serving sizes were 200 g for liquid dairy foods and 20 g for solid dairy foods. CI, confidence interval; RR, relative risk.

Total dairy intake (9 cohorts, 6 articles) was nonlinearly inversely associated with prediabetes in the most adjusted models (*P*_nonlinearity_ < 0.0001, *I*^2^ = 18%) (Table 2; Figure 2), but CoE was rated as low (Supplemental Table 7). Consuming 1 serving/d of total dairy was associated with a 13% lower risk of prediabetes (RR: 0.87 95% CI: 0.78, 0.96), and the lowest risk (25%) at 3.4 servings/d (RR: 0.75, 95% CI: 0.60, 0.93). The association weakened at higher intakes.

Neither high-fat nor low-fat dairy intake showed a statistically significant association with prediabetes risk (RR per serving/d: 0.99, 95% CI: 0.96, 1.02, *I*^2^ = 37%, low CoE, and *P*_nonlinearity_ < 0.0001, RR at 5.2 servings/d: 0.93, 95% CI: 0.66, 1.32, *I*^2^ = 28%, very low CoE, respectively) (Supplemental Figure 20). There was no evidence for publication bias in the meta-analyses for total, high-fat and low-fat dairy, as indicated by the funnel and DOI plots (Supplemental Figures 21–23).

#### Fermented dairy and prediabetes

No associations with prediabetes were found for fermented dairy intake, irrespective of fat content (Table 2; Supplemental Figure 24), there was no significant heterogeneity or evidence for publication bias (Supplemental Figures 25–27), and the CoE was rated as low (Supplemental Table 7).

#### Milk and prediabetes

No associations were observed for the consumption of total milk, high-fat milk, and low-fat milk with prediabetes risk (Table 2; Supplemental Figure 28). The CoE was rated as very low for total and high-fat milk, and as low for low-fat milk (Supplemental Table 7). For total and low-fat milk, there was moderate but nonsignificant heterogeneity, with *I*^2^ of 37% and 30%, respectively, and no evidence was found for publication bias (Supplemental Figures 29 and 30). Sensitivity analyses showed that with the exclusion of the Framingham Heart Study-Offspring Cohort (FHS-OC) from the meta-analysis, low-fat milk was associated with a 7% higher risk at 1.5 servings/d (RR: 1.07, 95% CI: 1.01, 1.14) (Supplemental Table 8). For high-fat milk, significant heterogeneity was observed (*I*^2^ = 59%, *P* = 0.01). Accounting for moderation by the prediabetes assessment method resulted in a slightly better model fit for total milk (*R*^2^ change 0.001–0.18) and high-fat milk (*R*^2^ change 0.03–0.33), indicating that part of these association was explained by the prediabetes assessment method used (Supplemental Table 9). This was especially noticeable in associations for high-fat milk. Cohorts that defined prediabetes based on FPG (FHS-OC) or FPG and non-FPG [Rotterdam Study (RS)] showed an inverse or no association between high-fat milk and prediabetes risk (Supplemental Figure 8). Conversely, cohorts defining prediabetes using FPG, 2hPG, and HbA1c [i.e., the Fenland study and Hoorn Study II (HS-II)] showed a positive association between high-fat milk and prediabetes risk. The assessment of publication bias in the association with high-fat milk yielded inconclusive results. The RS-II was an outlier in the funnel plot, although Egger’s test was not significant (*P* = 0.45), and the LFK index indicated minor asymmetry (Supplemental Figure 31).

#### Yogurt and prediabetes

Total, high-fat and low-fat yogurt were not associated with prediabetes risk in the most adjusted models (Table 2; Supplemental Figure 32), graded as very low CoE (Supplemental Table 7). In the minimally adjusted model, total yogurt was linearly associated with lower prediabetes risk (RR per serving/d: 0.90, 95% CI: 0.81, 0.99, *I*^2^ = 8%), but this association attenuated in models including adjustments for sociodemographic and health factors (Supplemental Table 10). Including the prediabetes assessment method as a moderator in the model improved the model fit for high-fat yogurt (*R*^2^ change 0.02–0.29) (Supplemental Table 9). Although none of the associations in the individual cohorts was statistically significant, a discernible pattern emerged (Supplemental Figure 11). In cohorts in which prediabetes was defined using FPG, 2hPG, and HbA1c (i.e., the Fenland study and HS-II), high-fat yogurt intake was associated with higher prediabetes risk. In contrast, in cohorts in which prediabetes was defined, based on the FPG and non-FPG (RS), an inverse association was found between high-fat yogurt intake and prediabetes risk, albeit not statistically significant. There was no evidence of publication bias in the meta-analyses of total and high-fat yogurt (Supplemental Figures 33 and 34). Evidence for publication bias for low-fat yogurt was inconclusive based on the funnel plot and DOI plot (Supplemental Figure 35).

#### Cheese and prediabetes

**FIGURE 3:**
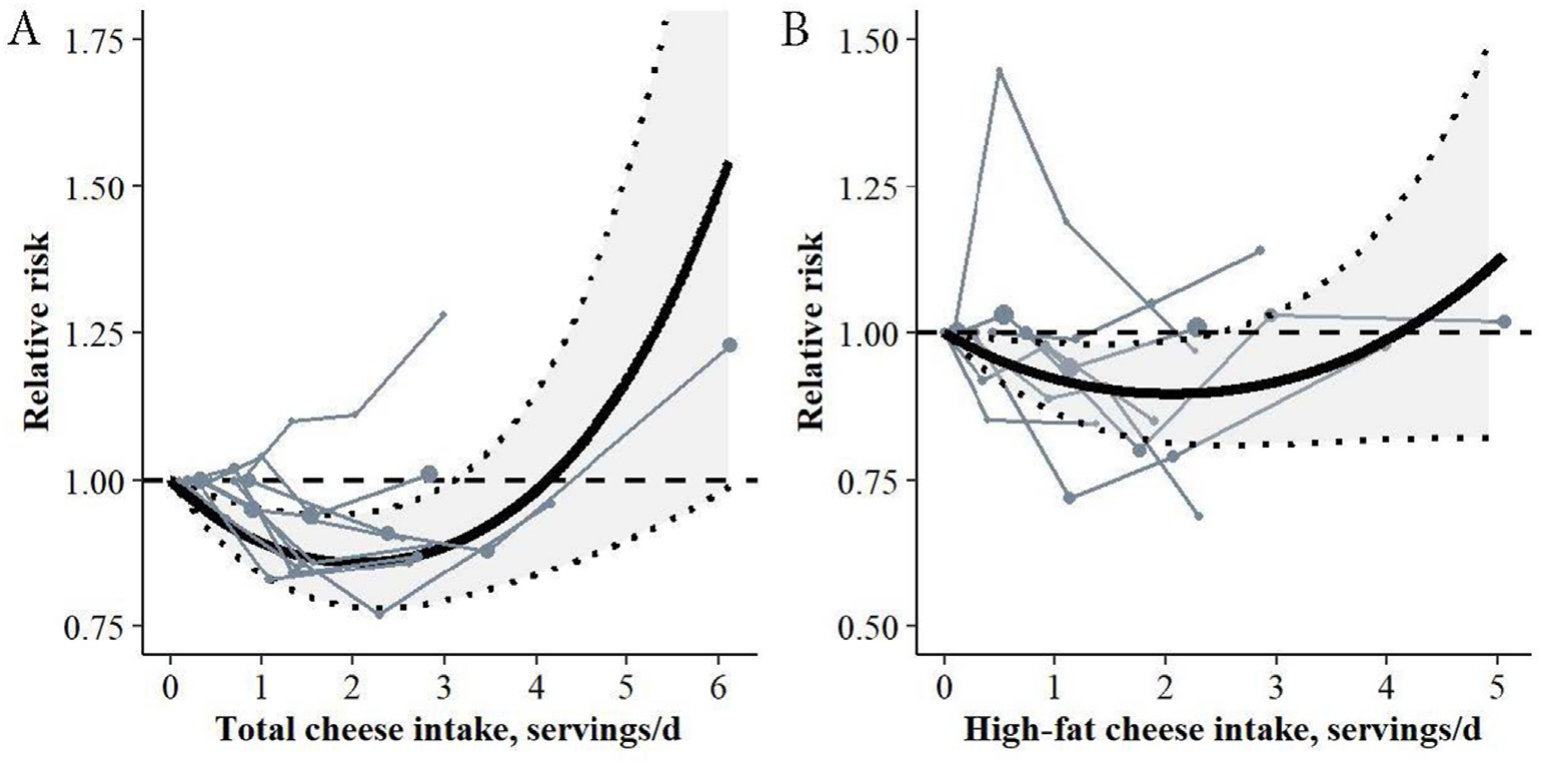
Spaghetti plot based on dose-response meta-analysis for the associations between (A) total cheese (lowest RR at 2.1 servings/d: 0.86, 95% CI: 0.78, 0.94, *I*^2^ = 0%, including 6 studies and 9 cohorts, 6653 cases among 95,844 participants) and (B) high-fat cheese (lowest RR at 2.1 servings/d: 0.90, 95% CI: 0.81, 0.99, *I*^2^ = 12%, including 5 studies and 8 cohorts, 5751 cases among 93,977 participants) and prediabetes risk. The solid black line represents the pooled RR at each quantity of intake. The light gray colored area between the dotted black lines indicates the 95% CI interval. The dashed gray line at RR = 1.00 represents the reference line. Each solid gray line represents a cohort with circles placed at the cohort-specific RRs at the corresponding intake level. The area of the circle is proportional to the study-specific weight. The associations were adjusted for age, sex, energy intake, educational level, smoking behavior, physical activity, alcohol intake, family history of diabetes, intake of food groups, waist circumference or BMI, hypertension, and dyslipidemia. A serving size of cheese was 20 g. CI, confidence interval; RR, relative risk.

A nonlinear inverse association was found between total and high-fat cheese intake and prediabetes risk (both *P*_nonlinearity_ < 0.0001) in the most adjusted models (Table 2; Figure 3A and B), both graded as low CoE (Supplemental Table 7). The risk of prediabetes was the lowest at intakes of 2.1 servings/d of total (RR: 0.86, 95% CI: 0.78, 0.94, *I*^2^ = 0%) and high-fat cheese intake (RR: 0.90, 95% CI: 0.81, 0.99, *I*^2^ = 12%), and increased to a RR of >1 at intakes of >4 servings/d. Some asymmetry was observed in the funnel plot and the LFK index indicated major asymmetry; studies reporting positive associations tended to have smaller SEs, whereas studies reporting inverse associations had higher SEs (Supplemental Figures 36 and 37). In sensitivity analyses, excluding the HS-1 cohort attenuated the association between high-fat cheese and prediabetes risk (RR at 1.8 servings/d: 0.93, 95% CI: 0.87, 1.01) (Supplemental Table 8), but not with total cheese. The prediabetes assessment method explained some additional variance for total cheese (*R*^2^ change from 0.46 to 0.68) and high-fat cheese (*R*^2^ change from 0.21 to 0.37) (Supplemental Table 9). However, differences in model fit were minor and no evident pattern was found to explain this finding.

For low-fat cheese intake, a linear positive association with prediabetes risk was observed in models adjusted for age, sex, energy intake, sociodemographic and health factors, and food group intake (RR per serving/d: 1.10, 95% CI: 1.02, 1.19, *I*^2^ = 51%, very low CoE) (Supplemental Table 10). In models additionally adjusted for BMI or waist circumference, hypertension, and dyslipidemia, this association attenuated (RR per serving/d: 1.05, 0.98, 1.14, *I*^2^ = 48%) (Table 2; Supplemental Figure 38). There was no evidence for publication bias (Supplemental Figure 39). Meta-regression analyses showed that the year of dairy intake assessment in individual studies explained some additional variance (*R*^2^ change from 0.17 to 0.50) (Supplemental Table 9). The 2 oldest cohorts, the HS-I and RS-I, both started in 1989, reported the highest risk for prediabetes with higher low-fat cheese intake, whereas the more recent cohorts did not report a statistically significant association (RS-III: 2006–2007, Fenland: 2005–2015 and Lifelines: 2006–2013) (Supplemental Figure 15).

#### Other dairy foods and prediabetes

Higher cream intake was not associated with prediabetes risk (RR per serving/d 0.85, 95% CI: 0.69, 1.05, *I*^2^ = 0%, very low CoE) (Table 2; Supplemental Figure 40). Only the FHS-OC had high dairy intake levels and thereby contributed to 94% of the weight in the meta-analysis (Supplemental Figure 16). Excluding this cohort from the model led to an additional risk reduction, although the association remained nonsignificant (RR: 0.73 per serving/d, 95% CI: 0.52, 1.02) (Supplemental Table 8). The RS-II and RS-III had extremely low median intake levels with high SEs resulting in major asymmetry of the funnel and DOI plot indicating potential publication bias (Supplemental Figure 41).

**FIGURE 4:**
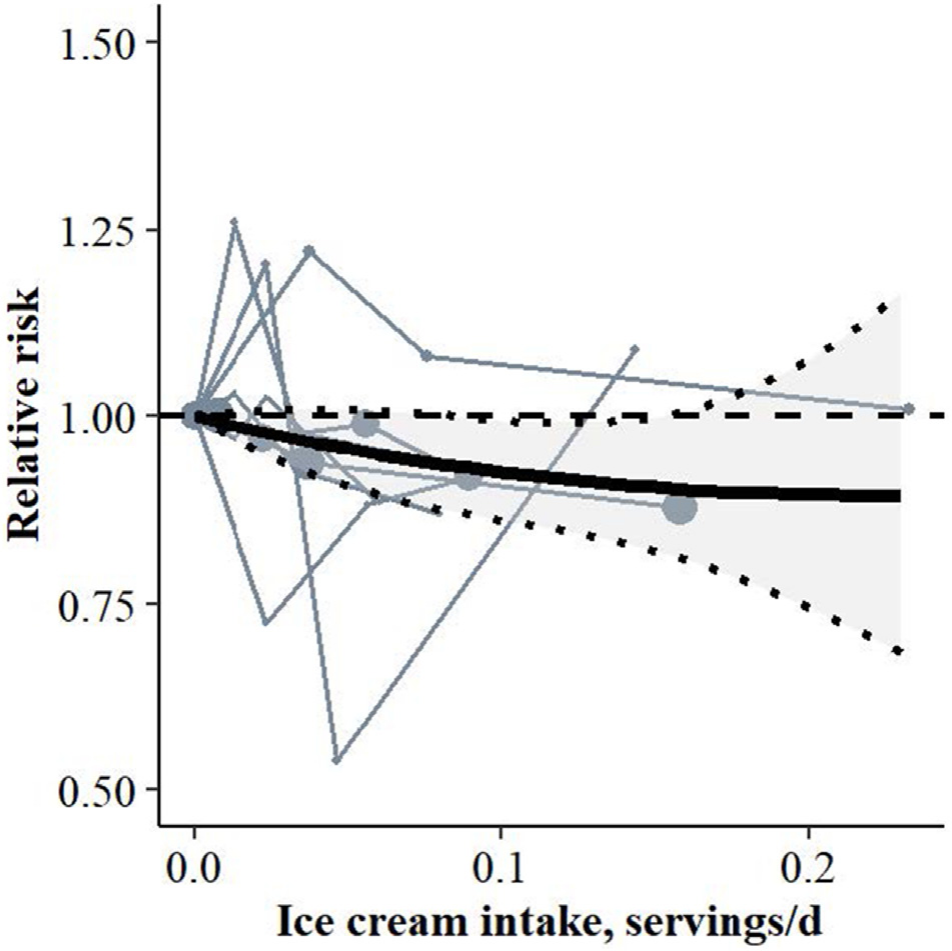
Spaghetti plot based on dose-response meta-analysis including 5 studies and 8 cohorts (6562 cases among 96,239 participants) for the associations between ice cream intake and prediabetes risk (RR per serving/d: 0.50, 95% CI: 0.26, 0.94, *I*^2^ = 0%). The solid black line represents the pooled RR at each quantity of intake. The light gray colored area between the dotted black lines indicates the 95% CI. The dashed gray line at RR = 1.00 represents the reference line. Each solid gray line represents a cohort with circles placed at the cohort-specific RRs at the corresponding intake level. The area of the circle is proportional to the study-specific weight. The associations were adjusted for age, sex, energy intake, educational level, smoking behavior, physical activity, alcohol intake, family history of diabetes, intake of food groups, waist circumference or BMI, hypertension and dyslipidemia. A serving size of ice cream was 150 g. CI, confidence interval; RR, relative risk.

A linear association was found between higher ice cream intake and lower prediabetes risk (RR per serving/d 0.50, 95% CI: 0.26, 0.94, *I*^2^ = 0%, low CoE) (Table 2; Figure 4). At the highest reported median intake of 0.23 servings/d, the RR was 0.85 (95% CI: 0.73, 0.99). However, this association was not statistically significant in models that were not adjusted for cardiometabolic risk markers (RR per serving/d: 0.57, 95% CI: 0.30, 1.08, *I*^2^ = 0.0%) (Supplemental Table 10). Minor asymmetry was identified in the funnel and DOI plot, with some smaller studies (HS-I and RS-I) reporting inverse associations, indicating some publication bias (Supplemental Figure 42).

### Dairy and glycemic markers

The characteristics, results, and conclusions of 14 prospective cohort studies that reported associations between dairy products and glycemic markers in a total of 38,441 participants are shown in Supplemental Table 11. Furthermore, 1 meta-analysis including 182,041 participants from 18 studies conducted in the United States, Denmark, Spain, Australia, and Finland was identified [28]. These studies were highly heterogeneous in analytical approaches, particularly in the use of baseline compared with changes in dairy intake and in defining the outcome at follow-up compared with change during follow-up. This variation prevented us from conducting meta-analyses on the relationship between dairy products and glycemic markers.

Baseline dairy intake in relation to glycemic markers at follow-up was reported in 8 studies; 2 for FPG [29,30], 2 for 2hPG [31,32], 2 for HbA1c [28,31], 3 for insulin resistance indices [5,28,31], and 3 for fasting insulin levels [28,30,33] with heterogeneous results. Inverse associations were observed in 2 studies, specifically for yogurt with FPG and fasting insulin levels [30], cheese and FPG, and fermented dairy and 2hPG [31]. Two studies reported positive associations between total dairy and HbA1c [28], and for total dairy, low-fat dairy, milk and milk products, and HOMA2-B [31]. Other studies did not report any statistically significant associations.

Baseline dairy intake in relation to changes in glycemic markers during follow-up was reported in 5 studies; 4 for changes in FPG [[34], [35], [36], [37]], 1 for changes in 2hPG [35], 1 for estimates of insulin resistance [37], and 1 for fasting insulin levels [37]. Inverse associations were found for total dairy and FPG [36], milk, yogurt, and cottage cheese intake and FPG changes in men only [34]. All other studies reported no statistically significant associations.

Changes in dairy in relation to changes in glycemic markers were reported in 2 studies; 1 for FPG and 2hPG [8], and 2 for HbA1c [8,38]. Both studies assessed a wide range of dairy types, most of which were not associated with the outcome. One study found that changes in low-fat dairy and low-fat milk intake were positively associated with changes in FPG and 2hPG during follow-up [8], whereas another study found that changes in high-fat milk intake were positively associated with changes in HbA1c [38]. Both studies also reported associations of linear mixed models with repeated outcome measures, in which several additional or inconsistent associations were found. For example, repeated measures of high-fat milk and cheese were inversely associated with repeated measures of HbA1c [38]. Two other studies also estimated associations of repeated measures of dairy and glycemic markers [5,39]. Total dairy and cheese intake were associated with lower insulin levels in a study that involved repeated assessments of insulin [39]. In contrast, another study measuring HOMA-IR found no statistically significant associations for total dairy and cheese. However, this study showed that high-fat yogurt was associated with lower HOMA-IR, whereas low-fat dairy, total milk, and low-fat milk were associated with higher HOMA-IR [5].

Eight studies were judged to have some concerns regarding the risk of bias, 3 studies were judged as being at high risk of bias and 2 at very high risk of bias (Supplemental Figures 18 and 19B). Five studies lacked sufficient adjustment for confounding factors [30,32,35,37,39]. Most studies used FFQs that were validated against other dietary assessment methods for dairy components (Supplemental Table 11). Repeated measures were available and accounted for in 5 studies [8,30,32,33,38]. The follow-up rate was <80% in 6 studies [8,30,31,35,36,38,39], or no information was given on follow-up rates in 2 studies [34,37], and 8 studies had considerable proportions of missing data [28,[30], [31], [32],[34], [35], [36], [37]].

## Discussion

The main findings of the dose-response meta-analysis of 6 articles based on 9 prospective cohorts indicated a nonlinear inverse association between total dairy intake and prediabetes risk. A 25% lower risk was identified at 3.4 servings/d, and the risk attenuated at higher intake levels, considering the limited data points available due to narrower intake ranges in underlying studies. Nevertheless, no associations were found for either high-fat or low-fat dairy intake. Furthermore, total and high-fat cheese were nonlinearly inversely associated with prediabetes risk, with a 14% and 10% lower risk at 2.1 servings/d, respectively, but a positive risk at intakes of >4 servings/d. A linear inverse association was found for ice cream intake. No associations were found for fermented dairy, milk, yogurt, and cream intake, irrespective of fat content.

Furthermore, a systematic review was conducted of 14 prospective cohort studies exploring the relationship between dairy consumption and glycemic outcomes. These studies presented a mix of inverse, positive, and null associations, reflected by the diversity in analytical strategies used. Thus, the existing body of evidence remains inconclusive regarding the relationship between subtypes of dairy and glycemic outcomes.

The reduced risk of prediabetes observed at 3.4 servings of total dairy/d appeared not to be attributable to a specific fat content. This finding is plausibly driven by inverse associations between total and high-fat cheese intake and prediabetes. This association was in the same direction as in a preliminary meta-analysis of total cheese and prediabetes of 3 studies also included in our meta-analysis (RR highest compared with lowest intake 0.93, 95% CI: 0.78, 1.12, *I*^2^ = 66%) [40]. As we utilized serving size definitions weighed for the liquid content, the relative contribution of cheese is higher, as opposed to operationalization in grams. This inverse association of total and high-fat cheese might relate to cheese matrix effects. Specific components, including proteins, milk fat globule membrane, medium- and branched chain fatty acids, calcium, and vitamin K2 are abundantly present in high-fat cheese, which may exert possible beneficial effects on glucose homeostasis [41]. In line with our findings, the most comprehensive meta-analysis, based on 25 prospective studies with type 2 diabetes as the outcome, showed that high compared with low total cheese intake was associated with lower risk (44,584 cases among 674,107 participants, RR: 0.93, 95% CI: 0.88, 0.98, *I*^2^ = 45%), but there was no evidence for a dose-response association (*n =* 18, 35,449 cases among 394,508 participants, RR per 30 g/d: 1.00 95% CI: 0.95, 1.06, *I*^2^ = 57%) [40]. Limited trial data to date, comparing cheese with different fat contents, suggested no effects on FPG, fasting insulin, and HOMA-IR [42,43].

The inverse association we observed between total dairy and prediabetes is consistent in direction with the associations found with type 2 diabetes, with the most complete meta-analysis showing a 3% lower risk per 200 g/d of total dairy (22 studies, RR: 0.97, 95% CI: 0.95, 1.00, *I*^2^ = 62.8%) [2]. However, these associations with type 2 diabetes are mainly driven by yogurt intake [40,44,45], whereas we did not observe associations between yogurt intake and prediabetes. Prior inverse associations have been attributed to beneficial effects of probiotics on weight and blood glucose regulation [41,46], although the precise impact depends on the specific probiotic strains and their dosage [47]. The dietary assessment methods used in the included studies often lack detailed information of specific yogurt types and sugar content. As a result, the inconsistent findings observed may be attributed to variations in the yogurt types consumed. Furthermore, the meta-analysis with type 2 diabetes as outcome found that the association was strongest in United States populations (*n* = 5, RR per 50 g/d 0.91; 95% CI: 0.86, 0.96) compared with Asian (0.95; 0.79, 1.14) and European (0.96; 0.92, 1.01) populations [48]. Most of these United States cohorts had limited yogurt intake with consumption being linked to healthy dietary patterns and behaviors [49,50]. In contrast, yogurt consumption is more common in European populations, as well as for the Dutch cohorts included in the current meta-analysis. A wider range of yogurt intake levels, along with greater variability in associated participant characteristics (i.e., age, diet, health factors), could weaken the observed associations.

A linear association between ice cream intake and prediabetes emerged only after adjusting for confounding effects of cardiometabolic risk factors. However, caution is needed in interpretation, given that the highest median intake level was <1 serving/wk (9 g/d), and the assessment is limited by seasonal variation. Similarly, a nonlinear inverse association between ice cream intake and type 2 diabetes risk was reported in a meta-analysis of 5 studies (19,730 cases among 258,571 participants) with the lowest risk observed at 10 g/d (RR: 0.81, 0.78–0.85, *I*^2^ = 85.7%) and no further decrease in risk found at higher intakes [48]. In 3 United States prospective cohorts, the inverse association of ice cream intake and type 2 diabetes attenuated when dietary data were no longer updated after participants reported a hypertension or hypercholesterolemia diagnosis [50]. Similarly, the potential for reverse causation, wherein dietary changes occur in response to cardiometabolic risk or a diagnosis, may explain our findings.

We observed no association between total and low-fat milk intake and prediabetes risk. For type 2 diabetes, the most comprehensive meta-analysis of observational studies similarly shows no associations for total, high-fat, or low-fat milk intake [2]. Also, no evidence for the causality of these associations was found in Mendelian randomization studies [51,52]. A recent meta-analysis (*n* = 24, 13,211 cases among 1,297,951 participants) showed that milk intake was associated with lower type 2 diabetes risk in non-White populations (RR per 245 g/d: 0.80, 95% CI: 0.66, 0.96), whereas in White populations, a modest positive association was found (RR per 245 g/d: 1.03, 95% CI: 1.01, 1.04) [53]. They suggested that variations in lactase persistence prevalence across populations could contribute to this heterogeneity and showed that this effect modification could stem from favorable alterations in gut microbiota and circulating metabolite profiles among individuals with lactase nonpersistence. The populations included in our meta-analysis were predominantly of White origin.

### Strengths and limitations

This is the first meta-analysis to investigate linear and nonlinear dose-response relationships of various dairy types, categorized by high compared with low-fat content, in association with prediabetes risk. We evaluated several factors that could explain heterogeneity between individual cohorts, including differences in confounder adjustments, baseline year, follow-up duration, and the prediabetes definition used. The results should be interpreted considering several limitations. First, the limited number of studies with low variation of intake for some dairy types may have led to overfitting, increasing the risk of spurious associations. Second, for meta-analyses of total and high-fat cheese, there were indications of publication bias. Third, the Lifelines study has a much larger sample size compared with the other cohorts and therefore received more weight in certain meta-analyses (e.g., 53.9% for fermented dairy). Fourth, given the observational nature of the included studies, the potential for residual confounding, reverse causation, and information bias cannot be dismissed. Fifth, the glycemic markers and cutoff values to define prediabetes differed across the cohort, which was a source of heterogeneity for associations of high-fat milk and high-fat yogurt with prediabetes. Finally, all studies were conducted in Western populations limiting the generalizability of findings to other ethnic backgrounds, low- or middle-income countries, and varying dietary patterns.

### Conclusion

In conclusion, the current evidence of population-based prospective cohort studies suggests that overall, dairy intake as measured at baseline, regardless of fat content, does not elevate the risk of prediabetes. Moderate beneficial associations were observed for total dairy intake, total cheese, and high-fat cheese. Milk, yogurt, and cream were not associated with prediabetes, irrespective of their fat content. The potential for reverse causation and residual confounding, especially considering our finding of an inverse linear association for ice cream intake, warrants the need for studies with comprehensive repeated measurements. Additionally, to inform more targeted preventive strategies and interventions, there is a need for RCTs validating potential underlying mechanisms and for exploring possible intra-individual variability in responses to dairy intake.

## Author contributions

The authors’ responsibilities were as follows – IALS, SSS-M: designed the research; IALS, YDV, BB: conducted research; IALS: performed statistical analysis and wrote the draft manuscript, supervised by SSS-M; IALS: has responsibility for the final content; and all authors: contributed to the study design, critically reviewed manuscript drafts, and approved the final manuscript.

## Conflict of interest

SSS-M has received unrestricted grants from the Global Dairy Platform, Dairy Research Institute, and Dairy Australia for a meta-analysis on cheese and blood lipids (2012) and a meta-analysis of dairy and mortality (2015). She received the Wiebe Visser International Dairy Nutrition Prize and has received recent research funding (2023) for epidemiological studies on dairy products and cardiometabolic diseases from the Dutch Dairy Association and the Danish Dairy Research Foundation. IALS has received recent research funding (2023) for epidemiological studies on dairy products and cardiometabolic diseases from the Dutch Dairy Association. YDV received funding for her PhD studentship from the Rank Prize Funds, the Dutch Dairy Association, and the Danish Dairy Research Foundation (2018). The other authors declare that they have no conflict of interest. Any prior sponsors had no role in the design and conduct of the study, data collection and analysis, interpretation of the data, decision to publish, or preparation of this manuscript. The current funder had no role in design and conduct of the study, data collection and analysis, interpretation of the data, or decision to publish.

## Funding

Dutch Dairy Association (NZO). The supporting source is not involved in the study design, collection, analysis, interpretation of data, writing the report, or restrictions regarding the submission of the report for publication.

## Data availability

Data described in the manuscript are available in the cited publications. The analytical code is publicly and freely available without restriction at: https://github.com/isabelslurink/MAdairyprediabetes.
