# Supplementary material for: Dairy Intake in Relation to Prediabetes and Continuous Glycemic Outcomes: A Systematic Review and Dose-Response Meta-Analysis of Prospective Cohort Studies
Source: Curr Dev Nutr. 2024 Sep 29;8(11):104470. doi: 10.1016/j.cdnut.2024.104470 (PMC11570412; doi:10.1016/j.cdnut.2024.104470)
Supplement: multimedia component 1 [file mmc1.docx]

**Dairy intake in relation to prediabetes and continuous glycemic outcomes: a systematic review and dose-response meta-analysis of prospective cohort studies**

Isabel Slurink^1^, Yakima D. Vogtschmidt^2^, Bo Brummel^1^, Tom Smeets^1^, Nina Kupper^1^, Sabita S. Soedamah-Muthu^1,3^

1. Center of Research on Psychological disorders and Somatic diseases (C*o*RPS), Department of Medical and Clinical Psychology, Tilburg University, PO Box 90153, 5000 LE, Tilburg, the Netherlands. [B.Brummel@tilburguniversity.edu](mailto:B.Brummel@tilburguniversity.edu); [T.Smeets@tilburguniversity.edu](mailto:T.Smeets@tilburguniversity.edu); [H.M.Kupper@tilburguniversity.edu](mailto:H.M.Kupper@tilburguniversity.edu); [S.S.Soedamah@tilburguniversity.edu](mailto:S.S.Soedamah@tilburguniversity.edu)
2. Hugh Sinclair Unit of Human Nutrition, Department of Food and Nutritional Sciences, University of Reading, Reading RG6 6DZ, UK; Institute for Cardiovascular and Metabolic Research, University of Reading, Reading RG6 6DZ, UK. Institute for Food, Nutrition and Health, University of Reading, Reading RG6 6EU, UK [y.d.vogtschmidt@pgr.reading.ac.uk](mailto:y.d.vogtschmidt@pgr.reading.ac.uk)
3. Institute for Food, Nutrition and Health, University of Reading, Reading RG6 6EU, UK.

# Table of contents

| **Supplemental table 1.** Search strategy. | 4 |
| --- | --- |
| **Supplemental table 2.** List of excluded studies. | 6 |
| **Supplemental table 3.** Definitions of dairy types included in each individual study included in the meta-analysis. | 7 |
| **Supplemental table 4.** Domains, signaling questions and decision criteria for each domain in ROBINS-E. | 9 |
| **Supplemental table 5.** Model fit and fit statistics for linear and nonlinear associations between dairy exposures and prediabetes risk. | 14 |
| **Supplemental table 6.** Characteristics and outcomes of separate two-stage fixed-effects dose-response meta-analyses, per dairy exposure. | 16 |
| **Supplemental table 7.** GRADE assessment for meta-analyses including prospective studies on the relation between dairy intake and prediabetes risk. | 17 |
| **Supplemental table 8.** Sensitivity analyses of associations between dairy foods and prediabetes risk based on one-stage random-effects dose-response meta-analysis. | 18 |
| **Supplemental table 9.** Goodness-of fit tests for models with potential moderators of the association between dairy foods and prediabetes risk based on one-stage linear or quadratic dose-response meta-regression | 20 |
| **Supplemental table 10.** Associations between dairy foods and prediabetes risk based on linear or quadratic two-stage random-effects dose-response meta-analysis for different confounder models. | 22 |
| **Supplemental table 11** Prospective cohort studies reporting associations between dairy foods and glycemic outcomes. | 24 |
| **Supplemental Figure 1.** Forest plot for the association between total dairy intake and prediabetes risk. | 30 |
| **Supplemental Figure 2.** Forest plot for the association between high-fat dairy intake and prediabetes risk. | 30 |
| **Supplemental Figure 3.** Forest plot for the association between low-fat dairy intake and prediabetes risk. | 31 |
| **Supplemental Figure 4.** Forest plot for the association between fermented dairy intake and prediabetes risk. | 31 |
| **Supplemental Figure 5.** Forest plot for the association between high-fat fermented dairy intake and prediabetes risk. | 32 |
| **Supplemental Figure 6.** Forest plot for the association between low-fat fermented dairy intake and prediabetes risk. | 32 |
| **Supplemental Figure 7.** Forest plot for the association between total milk intake and prediabetes risk. | 33 |
| **Supplemental Figure 8.** Forest plot for the association between high-fat milk intake and prediabetes risk. | 33 |
| **Supplemental Figure 9.** Forest plot for the association between low-fat milk intake and prediabetes risk. | 34 |
| **Supplemental Figure 10.** Forest plot for the association between total yogurt intake and prediabetes risk. | 34 |
| **Supplemental Figure 11.** Forest plot for the association between high-fat yogurt intake and prediabetes risk. | 35 |
| **Supplemental Figure 12.** Forest plot for the association between low-fat yogurt intake and prediabetes risk. | 35 |
| **Supplemental Figure 13.** Forest plot for the association between total cheese intake and prediabetes risk. | 36 |
| **Supplemental Figure 14.** Forest plot for the association between high-fat cheese intake and prediabetes risk. | 36 |
| **Supplemental Figure 15.** Forest plot for the association between low-fat cheese intake and prediabetes risk. | 37 |
| **Supplemental Figure 16.** Forest plot for the association between cream intake and prediabetes risk. | 37 |
| **Supplemental Figure 17.** Forest plot for the association between ice cream intake and prediabetes risk. | 38 |
| **Supplemental figure 18:** Risk of bias of each study for each domain and overall assessment based on the assessed with the ROBINS-E tool. | 39 |
| **Supplemental figure 19. O**verall risk of bias of the included studies assessed with the ROBINS-E tool. | 40 |
| **Supplemental figure 20.** Spaghetti plot based on dose-response meta-analysis for the associations between high-fat dairy, and low-fat dairy intake and prediabetes risk. | 41 |
| **Supplemental figure 21.** Contour-enhanced funnel plot and Doi plot for studies of the association between total dairy intake and prediabetes risk. | 42 |
| **Supplemental figure 22.** Contour-enhanced funnel plot and Doi plot for studies of the association between high-fat dairy intake and prediabetes risk. | 42 |
| **Supplemental figure 23.** Contour-enhanced funnel plot and Doi plot for studies of the association between low-fat dairy intake and prediabetes risk. | 43 |
| **Supplemental figure 24.** Spaghetti plot based on dose-response meta-analysis for the associations between **f**ermented dairy, high-fat fermented dairy, and low-fat fermented dairy intake and prediabetes risk. | 44 |
| **Supplemental figure 25.** Contour-enhanced funnel plot and Doi plot for studies of the association between fermented dairy intake and prediabetes risk. | 45 |
| **Supplemental figure 26.** Contour-enhanced funnel plot and Doi plot for studies of the association between high-fat fermented dairy intake and prediabetes risk. | 45 |
| **Supplemental figure 27.** Contour-enhanced funnel plot and Doi plot for studies of the association between low-fat fermented dairy intake and prediabetes risk. | 46 |
| **Supplemental figure 28.** Spaghetti plot based on dose-response meta-analysis for the associations between total milk, high-fat milk, and low-fat milk intake and prediabetes risk. | 47 |
| **Supplemental figure 29.** Contour-enhanced funnel plot and Doi plot for studies of the association between total milk intake and prediabetes risk. | 48 |
| **Supplemental figure 30.** Contour-enhanced funnel plot and Doi plot for studies of the association between low-fat milk intake and prediabetes risk. | 48 |
| **Supplemental figure 31.** Contour-enhanced funnel plot and Doi plot for studies of the association between high-fat milk intake and prediabetes risk. | 49 |
| **Supplemental figure 32.** Spaghetti plot based on dose-response meta-analysis for the associations between total yogurt, high-fat yogurt, and low-fat yogurt intake and prediabetes risk. | 50 |
| **Supplemental figure 33.** Contour-enhanced funnel plot and Doi plot for studies of the association between total yogurt intake and prediabetes risk. | 51 |
| **Supplemental figure 34.** Contour-enhanced funnel plot and Doi plot for studies of the association between high-fat yogurt intake and prediabetes risk. | 51 |
| **Supplemental figure 35.** Contour-enhanced funnel plot and Doi plot for studies of the association between low-fat yogurt intake and prediabetes risk. | 52 |
| **Supplemental figure 36.** Contour-enhanced funnel plot and Doi plot for studies of the association between total cheese intake and prediabetes risk. | 53 |
| **Supplemental figure 37.** Contour-enhanced funnel plot and Doi plot for studies of the association between high-fat cheese intake and prediabetes risk. | 53 |
| **Supplemental figure 38.** Spaghetti plot based on dose-response meta-analysis for the associations between low-fat cheese and prediabetes risk. | 54 |
| **Supplemental figure 39.** Contour-enhanced funnel plot and Doi plot for studies of the association between low-fat cheese intake and prediabetes risk. | 55 |
| **Supplemental figure 40.** Spaghetti plot based on dose-response meta-analysis for the associations between cream intake and prediabetes risk. | 56 |
| **Supplemental figure 41.** Contour-enhanced funnel plot and Doi plot for studies of the association between cream intake and prediabetes risk. | 57 |
| **Supplemental figure 42.** Contour-enhanced funnel plot and Doi plot for studies of the association between ice cream intake and prediabetes risk. | 57 |
| **References** | 58 |

| **Supplemental table 1.** Search strategy. | | |
| --- | --- | --- |
| **Database** | **Search terms** | **Hits** |
| PubMed | **Filters: English**  (“diabetes Mellitus“[MeSH Terms] OR “diabetes“[tiab] OR diabetic[tiab] OR NIDDM[tiab] OR “prediabetic state“[MeSH Terms] OR prediabetes[tiab] OR prediabetic OR “pre-diabetes”[tiab] OR “pre-diabetic” OR “hyperglycemia”[MeSH Terms] OR hyperglycemia[tiab] OR hyperglycaemia[tiab] OR “glucose intolerance“[MeSH Terms] OR glucose intolerance[tiab]OR metabolic syndrome[tiab] OR impaired glycemia[tiab] OR impaired glycaemia[tiab] OR impaired glucose tolerance[tiab] OR impaired fasting glucose[tiab] OR glucose[tiab] OR blood sugar[tiab] OR FPG[tiab] OR OGTT[tiab] OR “glycated hemoglobin“[MESH] OR glycated hemoglobin[tiab] OR "hemoglobin A"[tiab] OR HbA1c[tiab] OR A1c[tiab] OR “insulin resistance“[MeSH Terms] OR insulin[tiab] OR HOMA-IR[tiab] OR HOMO-IR[tiab] OR “Matsuda index”[tiab] OR ISI-M[tiab] OR “Stumvoll metabolic clearance rate”[tiab] OR “Stumvoll MCR”[tiab:~0] OR “Stumvoll insulin sensitivity index”[tiab] OR OGIS[tiab] OR “Gutt index”[tiab]) AND (dairy product[MeSH Terms] OR cultured milk product[MeSH Terms] OR cheese [MeSH Terms] OR yogurt[MeSH Terms] OR ice cream[MeSH Terms] OR milk[MeSH Terms] OR dairy[tiab] OR milk*[tiab] OR cheese*[tiab] OR yoghurt* [tiab] OR yogurt*[tiab] OR butter*[tiab] OR buttermilk*[tiab] OR cream*[tiab] OR sherbet*[tiab]OR quark*[tiab] OR custard*[tiab] OR curd*[tiab] OR pudding*[tiab] OR porridge*[tiab] OR ice cream*[tiab] OR icecream*[tiab] OR "ice-cream")  AND (longitudinal[tiab] OR cohort[tiab] OR follow-up[tiab] OR prospective[tiab]) | 1,281 |
| Scopus | **Filters: English**  (TITLE-ABS-KEY (diabetes OR diabetic OR NIDDM OR "prediabetic state" OR prediabetes OR prediabetic OR “pre-diabetes” OR “pre-diabetic” OR "insulin resistance" OR hyperglycemia OR hyperglycaemia OR "glucose intolerance" OR "metabolic syndrome" OR "impaired glycemia" OR "impaired glycaemia" OR "impaired glucose tolerance" OR "impaired fasting glucose" OR glucose OR "blood sugar" OR FPG OR OGTT OR glycated hemoglobin OR "hemoglobin A" OR hba1c OR a1c OR “insulin resistance” OR insulin OR HOMA-IR OR HOMO-IR OR “Matsuda index” OR ISI-M OR “Stumvoll metabolic clearance rate” OR “Stumvoll MCR” OR “Stumvoll insulin sensitivity index” OR OGIS OR “Gutt index”) AND TITLE-ABS-KEY (dairy OR milk* OR cheese* OR yoghurt* OR yogurt* OR butter* OR buttermilk* OR cream* OR sherbet* OR quark* OR custard* OR curd* OR pudding* OR porridge* OR "ice cream*" OR icecream* OR “ice-cream*”) AND TITLE-ABS-KEY (longitudinal OR cohort OR follow-up OR prospective)) | 1,242 |
| Web of Science | **Filters: English**  TS=(diabetes OR diabetic OR NIDDM OR “prediabetic state“ OR prediabetes OR prediabetic OR “pre-diabetes” OR “pre-diabetic” OR ”insulin resistance” OR hyperglycemia OR hyperglycaemia OR ”glucose intolerance” OR ”metabolic syndrome” OR ”impaired glycemia” OR ”impaired glycaemia” OR ”impaired glucose tolerance” OR ”impaired fasting glucose” OR glucose OR “blood sugar” OR FPG OR OGTT OR “glycated hemoglobin” OR ”hemoglobin A” OR HbA1c OR A1c OR “insulin resistance” OR insulin OR HOMA-IR OR HOMO-IR OR “Matsuda index” OR ISI-M OR “Stumvoll metabolic clearance rate” OR “Stumvoll MCR” OR “Stumvoll insulin sensitivity index” OR OGIS OR “Gutt index”) AND TS=(dairy OR milk* OR cheese* OR yoghurt* OR yogurt* OR butter* OR buttermilk* OR cream* OR sherbet* OR quark* OR custard* OR curd* OR pudding* OR porridge* OR ”ice cream*” OR icecream* OR “ice-cream”)  AND TS=(longitudinal OR cohort OR follow-up OR prospective) | 1,867 |
| Cochrane Library | #1 MeSH descriptor: [Diabetes Mellitus, Type 2] explode all trees  #2 MeSH descriptor: [Prediabetic State] explode all trees  #3 MeSH descriptor: [Insulin Resistance] explode all trees  #4 MeSH descriptor: [Hyperglycemia] explode all trees  #5 MeSH descriptor: [Glucose Intolerance] explode all trees  #6 MeSH descriptor: [Glycated Hemoglobin] explode all trees  #7 (diabetes OR diabetic OR NIDDM OR prediabetes OR prediabetic OR “pre-diabetes” OR “pre-diabetic” OR hyperglycemia OR hyperglycaemia OR “glucose intolerance” OR “metabolic syndrome“ OR “impaired glycemia” OR “impaired glycaemia” OR “impaired glucose tolerance” OR “impaired fasting glucose” OR glucose OR “blood sugar” OR FPG OR OGTT OR “glycated hemoglobin” OR “hemoglobin A” OR HbA1c OR A1c OR insulin OR HOMA-IR OR HOMO-IR OR “Matsuda index” OR ISI-M OR “Stumvoll metabolic clearance rate” OR “Stumvoll MCR” OR “Stumvoll insulin sensitivity index” OR OGIS OR “Gutt index”):ti,ab,kw  #8 MeSH descriptor: [Dairy Products] explode all trees  #9 MeSH descriptor: [Cultured Milk Products] explode all trees  #10 MeSH descriptor: [Cheese] explode all trees  #11 MeSH descriptor: [Yogurt] explode all trees  #12 MeSH descriptor: [Ice Cream] explode all trees  #13 MeSH descriptor: [Milk] explode all trees  #14 (dairy OR milk* OR cheese* OR yoghurt* OR yogurt* OR butter* OR buttermilk* OR cream* OR sherbet*OR quark* OR custard* OR curd* OR pudding* OR porridge* OR “ice cream*” OR icecream* OR “ice-cream*”):ti,ab,kw  #15 (longitudinal OR cohort OR follow-up OR prospective):ti,ab,kw (Word variations have been searched)  #16 #1 OR #2 OR #3 OR #4 OR #5 OR #6 OR #7  #17 #8 OR #9 OR #10 OR #11 OR #12 OR #13 OR #14  #18 #16 AND #17 AND #15 | 662 |
| Google Scholar | (prediabetes OR insulin OR glycemia OR glucose OR hemoglobin OR HbA1c) AND (dairy OR milk OR cheese OR yogurt OR yoghurt OR ice OR cream) AND (longitudinal OR cohort OR follow-up OR prospective) | ~1.310.000 |

| **Supplemental table 2.** List of excluded studies | |
| --- | --- |
| Reason for exclusion | References |
| No prediabetes diagnosis or continuous glycemic markers as outcome measures | (1-96) |
| No dairy food as exposure measure | (1, 8, 12, 14, 16, 18, 22, 28, 29, 31, 32, 41-43, 46-50, 58, 62-64, 69, 70, 72, 76, 78, 80, 83, 84, 86-91, 93-95, 97-131) |
| Not an observational, prospective study | (6, 9, 12, 14, 18, 19, 38, 39, 47, 63, 67, 77, 87, 89, 91, 97, 101, 104, 121, 122, 126-128, 132-142) |
| Participants with hyperglycemia | (47, 143, 144) |
| No full-text available | (27) (44, 145) |
| Non-English language | (3, 67) |
| Pediatric sample | (109) |

| **Supplemental table 3.** Definitions of dairy types included in each individual study included in the meta-analysis. | |
| --- | --- |
| **Dairy type** | **Definition** |
| **Hruby, 2017 (146). Framingham Heart Study Offspring Cohort, United States.** | |
| Total dairy | Foods made from milk that retain their calcium content, including milk, sherbet and ice milk, ice cream, yogurt, cottage and ricotta cheese, and other cheese |
| High-fat dairy | Skim milk, sherbet and ice milk, and yogurt |
| Low-fat dairy | Whole milk, ice cream, cottage and ricotta cheese, and other cheese |
| Total milk | Full-fat and skim and low-fat milk |
| High-fat milk | Full-fat milk |
| Low-fat milk | Skim and low-fat milk |
| Total yogurt | Yogurt |
| Total cheese | Cottage and ricotta cheese and other cheese |
| Cream | Foods made from milk that do not retain calcium, including cream, sour cream, cream cheese, and butter |
| **Slurink, 2022 (147). Hoorn Studies, The Netherlands** | |
| Total dairy | All dairy products |
| High-fat dairy | All high-fat dairy products |
| Low-fat dairy | All low-fat dairy products |
| Fermented dairy | All fermented products |
| High-fat fermented dairy | HS1 and HS2: full fat yogurt, full fat fruit yogurt, full fat curd, high fat cheese, full fat luxury cheese; HS2: oatmeal porridge, rice porridge and full fat custard |
| Low-fat fermented dairy | HS1 and HS2: Semi-skimmed yogurt, skimmed yogurt, skimmed fruit yogurt, semi-skimmed curd, skimmed curd, semi-skimmed fruit curd, skimmed fruit curd, low fat cheese, low fat luxury cheese, buttermilk. HS1: buttermilk; HS2: buttermilk porridge and skimmed custard. |
| Total milk | All milk and milk products |
| High-fat milk | HS1 and HS2: Full fat milk, full fat chocolate milk, milk powder, full fat milk added to the coffee; HS2: drinking yogurt, fruit flavoured milk. |
| Low-fat milk | Semi-skimmed milk, skimmed milk, buttermilk, semi-skimmed chocolate milk, skimmed chocolate milk, semi-skimmed milk added to the coffee, skimmed milk added to the coffee, semi-skimmed fruit milk |
| Total yogurt | All yogurts |
| High-fat yogurt | Full fat yogurt, full fat fruit yogurt |
| Low-fat yogurt | Semi-skimmed yogurt, skimmed yogurt, skimmed fruit yogurt |
| Total cheese | All cheeses |
| High-fat cheese | HS1: regular cheese, cheese cubes; HS2: 40 + cheese (e.g. Edam), 48 + cheese (e.g. Gouda, cheddar, cheese spread, goat  cheese), full fat luxury cheese (e.g. cream brie, cream cheese, mon chou), cheese cubes, grated cheese, feta, cheese fondue |
| Low-fat cheese | HS1: skimmed cheese; HS2: 20 + and 30 + cheese (e.g. cheese spread, cottage cheese), low fat luxury cheese (e.g. brie, goat cheese) |
| Cream | Whipped cream, coffee cream, semi-skimmed coffee cream, sour cream, crème fraiche, cooking cream |
| Ice cream | Ice cream |
| **Slurink, 2023 (148). AusDiab, Australia** | |
| Total dairy | All dairy products |
| High-fat dairy | High-fat milk, high-fat cheese, ice cream, yogurt (50% of intake) |
| Low-fat dairy | Low-fat milk, low-fat cheese, yogurt (50% of intake) |
| Fermented dairy | Yogurt, hard cheese, firm cheese, soft cheese, low-fat cheese |
| Total milk | High-fat milk, low-fat milk, flavored milk |
| High-fat milk | Full-fat milk (full cream milk) |
| Low-fat milk | Reduced fat milk, skimmed milk |
| Yogurt | Yogurt |
| Total cheese | All cheeses |
| High-fat cheese | Hard cheese, firm cheese, soft cheese, cream cheese |
| Low-fat cheese | Low-fat cheese, ricotta, or cottage cheese |
| Ice cream | Ice cream |
| **Slurink, 2022 (149). Rotterdam Study, The Netherlands.** | |
| Total dairy | All dairy products |
| High-fat dairy | All sub cohorts: all high-fat dairy products; RS-I and RS-III: full fat custard. |
| Low-fat dairy | All sub cohorts: all low-fat dairy products; RS-I: skimmed custard. |
| Fermented dairy | All fermented products |
| High-fat fermented dairy | All sub cohorts: high-fat yogurt, high-fat cheese, full fat curd, full fat fruit curd; RS-III: mousse and chipolata pudding. |
| Low-fat fermented dairy | Low-fat yogurt, low-fat cheese, semi-skimmed curd, skimmed curd, semi-skimmed fruit curd, skimmed fruit curd |
| Total milk | All milk and milk products |
| High-fat milk | Full fat milk, full fat chocolate milk, milk powder, full fat milk added to the coffee |
| Low-fat milk | Semi-skimmed milk, skimmed milk, buttermilk, semi-skimmed chocolate milk, skimmed chocolate milk, semi-skimmed milk added to the coffee, skimmed milk, added to the coffee |
| Total yogurt | All yogurts |
| High-fat yogurt | Full fat yogurt, full fat fruit yogurt |
| Low-fat yogurt | All sub-cohorts: Semi-skimmed yogurt, skimmed yogurt, semi-skimmed fruit yogurt skimmed fruit yogurt; RS-III: skimmed custard, semi-skimmed curd, skimmed curd, semi-skimmed fruit curd and skimmed fruit curd. |
| Total cheese | All cheeses |
| High-fat cheese | 40+ cheese (e.g., Edam), 48+ cheese (e.g. Gouda, cheddar, cheese spread, goat cheese), full fat luxury cheese (e.g. cream brie, cream cheese, mon chou), cheese cubes, grated cheese, feta, cheese fondue |
| Low-fat cheese | 20+ and 30+ cheese (e.g., cheese spread, cottage cheese), low-fat luxury cheese (e.g. brie, goat cheese) |
| Cream | Whipped cream, coffee cream, semi-skimmed coffee cream, sour cream, crème fraiche, cooking cream |
| Ice cream | Ice cream |
| **Slurink, 2023 (150). Lifelines Study, The Netherlands.** | |
| Total dairy | All dairy products |
| High-fat dairy | All high-fat dairy products |
| Low-fat dairy | All low-fat dairy products |
| Fermented dairy | All fermented dairy products |
| High-fat fermented dairy | High-fat yogurt, high-fat cheese, curd cheese |
| Low-fat fermented dairy | Low-fat yogurt, low-fat cheese, buttermilk |
| Total milk | Full fat milk, skimmed milk, semi skimmed milk, buttermilk, chocolate milk, coffee milk, plain milk in coffee |
| High-fat milk | Full fat milk |
| Low-fat milk | Skimmed milk, semi skimmed milk |
| Total yogurt | All yogurts |
| High-fat yogurt | Full fat natural yogurt |
| Low-fat yogurt | Skimmed yogurt, skimmed fruit yogurt |
| Total cheese | All cheeses |
| High-fat cheese | 40+ (spreadable) cheese, 48+ (spreadable) cheese, cream cheese, foreign cheeses, cheese cubes, (cream) cheese on baguette and on pieces of toast, grated cheese, diced cheese, feta cheese, cheese fondue |
| Low-fat cheese | 20+/30+ (spreadable) cheese |
| Cream | Whip cream, coffee cream |
| Ice cream | Milk-based ice cream |
| **Slurink, 2024 (151). Fenland Study, United Kingdom.** | |
| Total dairy | All dairy products |
| High-fat dairy | High-fat milk; high-fat yogurt; high-fat cheese; total cream; ice cream; milk puddings, e.g. rice, custard, trifle; dairy desserts |
| Low-fat dairy | Low-fat milk; low-fat yogurt; low-fat cheese |
| Fermented dairy | High-fat fermented dairy; low-fat fermented dairy |
| High-fat fermented dairy | High-fat yogurt; high-fat cheese |
| Low-fat fermented dairy | Low-fat yogurt; low-fat cheese |
| Total milk | High-fat milk; low-fat milk |
| High-fat milk | Full cream, silver; Channel Islands, gold, non-specific milk (50%) of intake |
| Low-fat milk | Semi-skimmed, red/white; dried milk, non-specific milk (50%) of intake |
| Total yogurt | High-fat yogurt; low-fat yogurt |
| High-fat yogurt | Full fat or Greek yogurt |
| Low-fat yogurt | Low-fat yogurt, fromage frais |
| Total cheese | High-fat cheese; low-fat cheese |
| High-fat cheese | Cheese, e.g. Cheddar, Brie, Edam |
| Low-fat cheese | Cottage cheese, low-fat soft cheese |
| Cream | Double or clotted cream, individual or sour cream |
| Ice cream | Ice cream, choc ices |
| Abbreviations: HS, Hoorn Studies (1^st^ and 2^nd^ enrolment wave); RS, Rotterdam Studies (1^st^, 2^nd^ and 3^rd^ enrolment wave). | |

| **Supplemental table 4.** Domains, signalling questions and decision criteria for each domain in ROBINS-E. | | |
| --- | --- | --- |
| **Domain** | **Signaling questions^1^** | **Decision criteria^1^** |
| **Bias due to confounding^2^** | - Did the authors control for all the important confounding factors for which this was necessary?   - Age, sex, total energy intake, and two or more additional domains, i.e., sociodemographic factors (e.g., SES/education, ethnicity), health factors (e.g. physical activity, alcohol intake, smoking, family history of diabetes), dietary factors and cardiovascular risk factors (e.g. body mass index). - Were confounding factors that were controlled for (and for which control was necessary) measured validly and reliably by the variables available in this study? - Did the authors control for any variables after the start of the exposure period being studied that could have been affected by the exposure? - Did the use of negative controls, or other considerations, suggest serious uncontrolled confounding?   Notes: **Confounding is expected in all observational studies, low risk of bias was not assigned to any study.** Time-varying confounding was expected to be unlikely and is not expected to cause risk of bias in the present study. | Low risk of bias: No bias expected due to confounding, including time-varying confounding.  Some concerns: All the important confounding factors for which it is was deemed necessary to control for were assessed using valid and reliable measures and included in a multivariable adjusted analysis.  High risk of bias: At least one known important confounding factor was not measured or appropriately controlled for, or the study controlled for post-exposure variables, or the use of negative controls suggest serious uncontrolled confounding. |
| **Bias arising from measurement of**  **exposure^2^** | - Does the measured exposure well-characterize the exposure metric specified to be of interest in this study?   - Baseline intake per serving or gram   - Change in intake per serving or gram between baseline and follow-up - Was the exposure likely to be measured with error, or misclassified? - Could mismeasurement or misclassification of exposure have been differential (i.e. related to the outcome or risk of the outcome)? - Is non-differential measurement error likely to bias the estimated effect of exposure on outcome?   Note: The start of follow-up is considered to coincide with the baseline exposure assessment. **Any dietary assessment method involves measurement error, thus, no study was assigned low risk of bias.** | Low risk of bias: Exposure status was well defined; and no measurement error is expected in its assessment.  Some concerns: Exposure status was well defined; and exposure was measured using a validated tool (structured interview/≥ 2 dietary recalls/ diet history/ food frequency questionnaire including at least 50 items, validated for dairy components).  High risk of bias: Exposure status is not well defined, e.g., lack of standardization, differential misclassification or measurement error, or exposure was measured using not validated tools (written self-report (e.g. <2 dietary recalls/non-validated food frequency questionnaire or not reported whether food frequency questionnaire was validated)) |
| **Bias due to selection of participants into**  **the study (or into the analysis)^2^** | - Did follow-up begin at (or close to) the start of the exposure window for most participants? - Was selection of participants into the study based on participants characteristics observed after start of the study/exposure assessment? - Were methods used that are likely to correct for the presence of selection biases?   Notes: In observational studies, it is unlikely that post-exposure variables influenced selection of participants into the study. Exclusion of participants may be mostly based on missing data, which will be considered in the domain referring to missing data (see below). The start of follow-up is considered to coincide with the baseline exposure assessment. However, participants are already exposed at start of the study, which might have influenced outcome measured that occurred shortly after start of the study. | Low risk of bias: All participants who would have been eligible for the target study were included in the study.  Some concerns: Selection into the study may have been related to exposure and outcome (e.g. inclusion of participants with a history of diabetes); and the authors used appropriate methods to correct for the selection bias.  High risk of bias: Selection into the study was related to exposure and outcome (e.g. only participants with any level of dairy intake were included); and this could not be corrected for in the analyses; or start of follow up and start of exposure do not coincide and the rate ratio is  not constant over time. |
| **Bias due to post-exposure interventions^2^** | - Were there deviations from the exposure beyond what would be expected in usual practice? - Were these deviations unbalanced between groups and likely to have affected the outcome?   Notes: Repeated measurements of the exposure are mostly not available in observational studies. It is not expected that there are significant changes in diet in healthy participants. It is likely that any changes in diet over time are comparable between different studies and similar across participant groups, reducing the likelihood of differential misclassification. Recent studies have shown that diet is constant or change only slightly over time (152, 153). Thus, if repeated measures are not available, some concerns could be assigned to a study. | Low risk of bias: **Repeated measurements of the exposure status during follow-up are available**. No or only slight changes were observed, and the changes were considered in the analysis.  Some concerns: Repeated measurements of the exposure are not available, but substantial changes are not expected during follow-up, or repeated measurements of the exposure status during follow-up are available and  no or only slight changes were observed which were not considered in analysis, or small changes were observed and the analysis appropriately accounted for these changes, estimating the effect of deviations in exposure that could potentially impact the outcome.  High risk of bias: Exposure status is measured during follow-up and substantial changes in the exposure have been observed, and the analysis was not appropriate to estimate the effect of changes, allowing for deviations that were likely to impact on the outcome. |
| **Bias due to missing data^2^** | - Were complete data on the outcome available for all, or nearly all, participants? - Were complete data on confounding variables available for all, or nearly all, participants? - Is the result based on a complete case analysis? - Were participants excluded due to missing data on other variables needed for analysis? - Was exclusion from the analysis because of missing data (in exposure, confounders or the outcome) likely to be related to the true value of the outcome? - Were all or most predictors of missingness (in exposure, confounders or the outcome) included in the analysis model? - Was the analysis based on imputing missing values? - Was imputation performed appropriately? - Was an appropriate alternative method used to correct for bias due to missing data? - Is there evidence that the result was not biased by missing data? | Low risk of bias: Little loss-to-follow-up (<20%) of missing data; and data on exposure and other variables were reasonably complete (<10% missing data) and was unlikely to introduce bias; or the analysis addressed missing data and is likely to have removed any risk of bias.  Some concerns: There is a proportion of missing data in the original cohort or a high proportion of loss-to-follow-up (>20%); and the analysis is unlikely to have removed the risk of bias arising from the missing data.  High risk of bias: High proportions (>50%) of missing data; and the analysis is unlikely to have removed the risk of bias arising from the missing data; or missing data were addressed inappropriately in the analysis; or the nature of the missing data means that the risk of bias cannot be removed through appropriate analysis. |
| **Bias arising from measurement of the outcome^2^** | - Could measurement or ascertainment of the outcome have differed between exposure groups or levels of exposure? - Were outcome assessors aware of study participants’ exposure history? - Could assessment of the outcome have been influenced by knowledge of participants’ exposure history?   Notes: In observational studies, it is not expected that outcome assessors were aware of exposure status of the participants | Low risk of bias: The methods of outcome assessment were comparable across exposure groups; and the outcome measure was unlikely to be influenced by knowledge of the exposure status of study participants; and any error in measuring the outcome is unrelated to exposure status (i.e. objective measures).   - Prediabetes: independent blind assessment/FPG/2hPG/HbA1c. - Glycemic outcomes: blood sampling or OGTT after 12h or overnight fast🟊  1. non-validated self-report 2. no description   Some concerns: The methods of outcome assessment were comparable across exposure groups; and any error in measuring the outcome may be minimally related to exposure status or if the outcome measure was not reliable measured (i.e. record linkage/medical record or validated self-report, confirmed records are not available for the whole study population).  High risk of bias: The methods of outcome assessment were not comparable across exposure groups; or the outcome measure was subjective (i.e. non-validated self-report); and error in measuring the outcome was related to exposure status. |
| **Bias in the selection of the result^2^** | - Is the reported effect estimate likely to be selected from multiple analyses of exposure-outcome relationship? - Is the reported effect estimate likely to be selected from different subgroups? | Low risk of bias: There is a clear description of all analysis, and the analyses are consistent, and all reported results correspond to all intended outcomes, analyses and sub-cohorts.  Some concerns: The analyses are clearly defined; and there is indication of selection of the reported analysis from among multiple analyses; and there is indication of selection of the cohort or subgroups for analysis and reporting on basis of the results (e.g. estimates not shown for all analyses).  High risk of bias: There is a high risk of selective reporting from among multiple analyses; or the cohort or subgroup is selected from a larger study for analysis and appears to be reported based on the results |
| **Overall judgement** | **Low risk of bias** | The study is judged to be at low risk of bias for all domains. |
|  | **Some risk of bias** | The study is judged to be at low or some concerns of bias for all domains. |
|  | **High risk of bias** | The study is judged to be at high risk of bias in at least one domain. |
|  | **Very high risk of bias** | The study is judged to be at high risk of bias in several domains. |
| ^1^ Signaling questions derived from the Risk Of Bias In Non-randomized Studies - of Exposure (ROBINS-E) tool (154), with decision criteria adapted from Kiesswetter *et al. (*2024) (155).  ^2^ Due to the nature of the included prospective observational studies; studies were not considered to be at very high risk of bias in single bias domains. | | |

| **Supplemental table 5.** Model fit and fit statistics for linear and nonlinear associations between dairy exposures and prediabetes risk.^1,2^ | | | | | |
| --- | --- | --- | --- | --- | --- |
| **Exposure** | **Model fit** | **p_nonlinearity_** | **Log likelihood** | **AIC** | **BIC** |
| **Total dairy** | Linear |  | 8.179 | -12.358 | -12.199 |
|  | Quadratic | <0.0001 | 23.752 | -37.504 | -33.641 |
|  | RCS | NS | 12.389; 19.053 | -15.129; 2.215 | -9.239; 23.106 |
| **High-fat dairy** | Linear |  | 8.440 | -12.880 | -12.721 |
|  | Quadratic | 0.006 | 12.267 | -14.533 | -10.670 |
|  | RCS | 0.001; NS | 11.926; 17.850 | -13.851; 13.302 | -7.757; 35.128 |
| **Low-fat dairy** | Linear |  | 9.827 | -15.654 | -15.496 |
|  | Quadratic | <0.0001 | 20.459 | -30.918 | -27.055 |
|  | RCS | NS | 15.874; 21.220 | -21.764; -1.954 | -15.670; 19.867 |
| **Fermented dairy** | Linear |  | 9.502 | -15.004 | -15.112 |
|  | Quadratic | <0.0001 | 18.490 | -26.979 | -23.784 |
|  | RCS | NS | 15.407; 20.287 | -20.961; 1.058 | -15.738; 18.865 |
| **High-fat fermented dairy** | Linear |  | 9.115 | -14.231 | -14.647 |
|  | Quadratic | 0.001 | 14.510 | -19.021 | -16.596 |
|  | RCS | 0.001; 0.3 | 15.013; 19.986 | -20.307; 0.370 | -15.585; 15.822 |
| **Low-fat fermented dairy** | Linear |  | 8.120 | -12.241 | -12.657 |
|  | Quadratic | 0.08 | 9.627 | -9.254 | -6.829 |
|  | RCS | 0.0002; 0.02 | 12.577; 19.093 | -15.350; -4.125 | -10.432; 7.540 |
| **Total milk** | Linear |  | 10.995 | -17.991 | -17.832 |
|  | Quadratic | <0.0001 | 20.919 | -31.839 | -27.976 |
|  | RCS | NS | 10.695; 18.293 | -11.617; 4.906 | -5.522; 26.726 |
| **High-fat milk** | Linear |  | 3.654 | -3.307 | -1.125 |
|  | Quadratic |  | 3.584 | 2.831 | 8.054 |
|  | RCS^3^ | <0.0001; 0.0003 | 5.718; 12.160 | -6.319; -1.437 | 2.642; 4.1 |
| **Low-fat milk** | Linear |  | 11.084 | -18.169 | -18.010 |
|  | Quadratic | <0.0001 | 19.955 | -29.910 | -26.046 |
|  | RCS | NS | 11.265; 13.941 | -12.722; 0.628 | -6.627; 16.525 |
| **Total yogurt** | Linear |  | 10.457 | -16.913 | -14.476 |
|  | Quadratic | <0.0001 | 11.945 | -13.890 | -8.000 |
|  | RCS | <0.0001 | 12.291; 24.846 | -21.692; -14.581 | -10.203; -5.848 |
| **High-fat yogurt** | Linear |  | 1.254 | 1.492 | 3.484 |
|  | Quadratic | 0.002 | 1.750 | 6.499 | 11.222 |
|  | RCS | <0.0001 | 2.613; 11.988 | 2.212; 6.991 | 9.496; 18.656 |
| **Low-fat yogurt** | Linear |  | 13.836 | -23.672 | -21.681 |
|  | Quadratic | <0.0001 | 12.984 | -15.968 | -11.246 |
|  | RCS | <0.0001 | 13.660; 22.136 | 17.520; -14.571 | -12.798; -3.763 |
| **Total cheese** | Linear |  | 9.409 | -14.818 | -14.659 |
|  | Quadratic | <0.0001 | 22.666 | -35.333 | -31.470 |
|  | RCS |  | 19.597; 24.455 | -29.195; -5.891 | -23.517; 14.024 |
| **High-fat cheese** | Linear |  | 7.386 | -10.772 | -10.881 |
|  | Quadratic | <0.0001 | 15.323 | -20.647 | -17.451 |
|  | RCS | 0.004; NS | 11.552; 19.510 | -13.522; 4.779 | -8.300; 22.586 |
| **Low-fat cheese** | Linear |  | 1.142 | -18.283 | -16.101 |
|  | Quadratic | 0.002 | 8.837 | -7.675 | -2.452 |
|  | RCS | <0.0001 | 10.775; 16.421 | -11.550; -4.841 | -6.327; 8.799 |
| **Cream** | Linear |  | 13.301 | -22.601 | -20.330 |
|  | Quadratic | <0.0001 | 12.219 | -14.438 | -8.983 |
|  | RCS | <0.0001 | 15.824; 38.478 | -36.955; -21.175 | -18.066; -8.537 |
| **Ice cream** | Linear |  | 17.993 | -31.986 | -29.804 |
|  | Quadratic | <0.0001 | 20.635 | -31.270 | -26.048 |
|  | RCS | <0.0001 | 19.766; 29.761 | -31.522; -27.352 | -24.310; -14.129 |
| **^1^** Eight RCS models with 3-6 knots were tested. The range of fit statistics are shown. ^2^ The final model was selected based on the AIC and BIC and is highlighted in grey. ^3^ Four RCS models were tested with three or four knots as five or six knots failed to converge. ^4^ A four-knot RCS model with knots placed at the 0.05^th^, 0.35^th^, 0.65^th^ and 0.95^th^ percentile. Abbreviations: Akaike information criterion; BIC, Bayesian information criterion; RCS, Restricted cubic spline. | | | | | |

| **Supplemental table 6.** Characteristics and outcomes of separate two-stage fixed-effects dose-response meta-analyses, per dairy exposure. | | | | | | | | | | | |
| --- | --- | --- | --- | --- | --- | --- | --- | --- | --- | --- | --- |
| **Exposure** | **No. cohorts (articles)** | **Total N** | **N cases** | **Mean follow-up (years)** | **Range median intake (servings/d)^1^** | **Model fit (p_nonlinearity_)** | **RR (95%CI) at 1 serving/d** | **Lowest or highest RR of quadratic fit (95%CI)** | **Heterogeneity** | | |
|  |  |  |  |  |  |  |  |  | **I^2^** | **Q test** | **p-value** |
| **Total dairy** | 9 (6) | 95,844 | 6,653 | 9.6 | 1.1-3.7 | Quadratic (p<0.0001) | 0.87 (0.80-0.94) | 0.75 (0.64-0.88) at 3.4 servings /d | 18.0% | 19.5 | 0.24 |
| **High-fat dairy** | 9 (6) | 95,844 | 6,653 | 9.6 | 0.3-2.0 | Linear | 0.99 (0.96-1.02) |  | 36.8% | 12.7 | 0.12 |
| **Low-fat dairy** | 9 (6) | 95,844 | 6,653 | 9.6 | 0.8-2.6 | Quadratic (p<0.0001) | 1.00 (0.95-1.05) | 0.91 (0.72-1.14) at 5.2 servings/d | 27.8% | 22.1 | 0.14 |
| **Fermented dairy** | 8 (5) | 93,975 | 5,751 | 9.5 | 0.7-2.4 | Quadratic (p<0.0001) | 0.93 (0.86-1.01) | 0.91 (0.81-1.02) at 2 servings/d | 0% | 9.4 | 0.81 |
| **High-fat fermented dairy** | 7 (4) | 89,089 | 4,986 | 9.1 | 0.8-1.7 | Quadratic (p=0.001) | 0.95 (0.88-1.02) | 0.94 (0.86-1.03) at 1.7 servings/d | 0% | 7.9 | 0.79 |
| **Low-fat fermented dairy** | 7 (4) | 89,089 | 4,986 | 9.1 | 0.2-0.7 | Linear | 0.98 (0.94-1.03) |  | 0% | 1.8 | 0.94 |
| **Total milk** | 9 (6) | 95,844 | 6,653 | 9.6 | 0.9-2.4 | Quadratic (p<0.0001) | 1.00 (0.95-1.06) | 0.98 (0.86-1.12) at 4.1 servings/d | 36.7% | 25.3 | 0.07 |
| **High-fat milk** | 9 (6) | 95,844 | 6,653 | 9.6 | 0.03-1.3 | Linear | 0.97 (0.92-1.03) |  | 58.5% | 19.3 | 0.01 |
| **Low-fat milk** | 9 (6) | 95,844 | 6,653 | 9.6 | 0.6-1.5 | Quadratic (p<0.0001) | 1.04 (0.99-1.09) | 0.91 (0.77-1.08) at 4.1 servings/d | 30.0% | 22.8 | 0.12 |
| **Total yogurt** | 9 (6) | 95,844 | 6,653 | 9.6 | 0.02-0.5 | Linear | 0.99 (0.91-1.07) |  | 7.9% | 8.7 | 0.37 |
| **High-fat yogurt** | 7 (4) | 89,086 | 4,986 | 9.6 | 0.2-0.4 | Linear | 1.06 (0.90-1.24) |  | 21.0% | 7.6 | 0.27 |
| **Low-fat yogurt** | 7 (4) | 89,086 | 4,986 | 9.6 | 0.09-0.4 | Linear | 1.01 (0.93-1.10) |  | 0.0% | 2.9 | 0.83 |
| **Total cheese** | 9 (6) | 95,844 | 6,653 | 9.6 | 0.6-2.9 | Quadratic (p<0.0001) | 0.92 (0.88-0.97) | 0.90 (0.83-0.97) at 2.1 servings/d | 0.0% | 12.6 | 0.70 |
| **High-fat cheese** | 8 (5) | 93,977 | 5,751 | 9.5 | 0.4-2.4 | Quadratic (p<0.0001) | 0.94 (0.89-0.99) | 0.92 (0.85-0.99) at 2.0 servings/d | 11.6% | 15.8 | 0.32 |
| **Low-fat cheese** | 8 (5) | 93,977 | 5,751 | 9.5 | 0.2-1.0 | Linear | 1.05 (1.00-1.10) |  | 48.0% | 13.5 | 0.06 |
| **Cream** | 8 (5) | 90,953 | 5,888 | 9,3 | 0.02-0.8 | Linear | 0.92 (0.85-0.99) |  | 0.0% | 6.2 | 0.51 |
| **Ice cream** | 8 (5) | 96,239 | 6,562 | 9,5 | 0.02-0.06 | Linear | 0.50 (0.26-0.94) |  | 0.0% | 2.4 | 0.93 |
| ^1^ For composite dairy types, serving sizes were 200 g for liquid dairy foods and 20 g for solid dairy foods. For individual dairy types, serving sizes were 150 g for milk, yogurt, and ice cream; 20 g for cheese, and 15 g for cream. | | | | | | | | | | | |

| **Supplemental table 7.** GRADE assessment for meta-analyses including prospective studies on the relation between dairy intake and prediabetes risk. | | | | | | | | | | | |  |
| --- | --- | --- | --- | --- | --- | --- | --- | --- | --- | --- | --- | --- |
|  | No. cohorts (articles) | **Downgrades** | | | | |  | **Upgrades** | |  | Certainty of evidence | |
| Exposure |  | Risk of bias | Inconsistency | Indirectness | Imprecision | Publication bias |  | Dose-response | Magnitude |  |  |  |
| Total dairy | 9 (6) | Some concerns^1^ | Not serious | Not serious | Not serious | Undetected |  | **Yes^6^** |  |  | ⊕⊕OO Low | |
| High-fat dairy | 9 (6) | Some concerns^1^ | Not serious | Not serious | Not serious | Undetected |  |  |  |  | ⊕⊕OO Low | |
| Low-fat dairy | 9 (6) | Some concerns^1^ | Not serious | Not serious | **Serious^3^** | Undetected |  |  |  |  | ⊕OOO Very low | |
| Fermented dairy | 8 (5) | Some concerns^1^ | Not serious | Not serious | Not serious | Undetected |  |  |  |  | ⊕⊕OO Low | |
| High-fat fermented dairy | 7 (4) | Some concerns^1^ | Not serious | Not serious | Not serious | Undetected |  |  |  |  | ⊕⊕OO Low | |
| Low-fat fermented dairy | 7 (4) | Some concerns^1^ | Not serious | Not serious | Not serious | Undetected |  |  |  |  | ⊕⊕OO Low | |
| Total milk | 9 (6) | Some concerns^1^ | Not serious | Not serious | **Serious^3^** | Undetected |  |  |  |  | ⊕OOO Very low | |
| High-fat milk | 9 (6) | Some concerns^1^ | **Serious^2^** | Not serious | **Serious^3^** | **Inconclusive^4^** |  |  |  |  | ⊕OOO Very low | |
| Low-fat milk | 9 (6) | Some concerns^1^ | Not serious | Not serious | Not serious | Undetected |  |  |  |  | ⊕⊕OO Low | |
| Total yogurt | 9 (6) | Some concerns^1^ | Not serious | Not serious | **Serious^3^** | Undetected |  |  |  |  | ⊕OOO Very low | |
| High-fat yogurt | 7 (4) | Some concerns^1^ | Not serious | Not serious | **Serious^3^** | Undetected |  |  |  |  | ⊕OOO Very low | |
| Low-fat yogurt | 7 (4) | Some concerns^1^ | Not serious | Not serious | **Serious^3^** | **Inconclusive^4^** |  |  |  |  | ⊕OOO Very low | |
| Total cheese | 9 (6) | Some concerns^1^ | Not serious | Not serious | Not serious | **Detected^5^** |  | **Yes^6^** |  |  | ⊕⊕OO Low | |
| High-fat cheese | 8 (5) | Some concerns^1^ | Not serious | Not serious | Not serious | **Detected^5^** |  | **Yes^6^** |  |  | ⊕⊕OO Low | |
| Low-fat cheese | 8 (5) | Some concerns^1^ | **Serious^2^** | Not serious | Not serious | Undetected |  |  |  |  | ⊕OOO Very low | |
| Cream | 8 (5) | Some concerns^1^ | Not serious | Not serious | **Serious^3^** | **Detected^5^** |  |  |  |  | ⊕OOO Very low | |
| Ice cream | 8 (5) | Some concerns^1^ | Not serious | Not serious | Not serious | **Detected^5^** |  | **Yes^6^** | **Yes^7^** |  | ⊕⊕OO Low | |
| **Explanations**  **^1^** Downgraded by one level for risk of bias, since all studies were judged to have some concerns regarding the risk of bias based on ROBINS-E (**Supplemental figure 1**).  ^2^ Downgraded by one level for inconsistency, due to substantial unexplained heterogeneity based on I² > 50% and a p-value for heterogeneity < 0.10  ^3^ Downgraded by one level for imprecision, as the 95%CIs include the minimally important difference (MID) of 5%, RR 0.95–1.05).  ^4^ Not downgraded by on level for publication bias, as evidence of small study effects was inconclusive.  ^5^ Downgraded by on level for publication bias, due to significant evidence of small study effects.  ^6^ Upgraded by one level for a dose-response gradient, as a statistically significant linear or non-linear associations was found per serving/day.  ^7^ Upgraded by one level for magnitude, due to a large risk estimate (RR < 0.5 or > 2). | | | | | | | | | | | |  |

| **Supplemental table 8.** Sensitivity analyses of associations between dairy foods and prediabetes risk based on one-stage random-effects dose-response meta-analysis.^1^ | | | |
| --- | --- | --- | --- |
| **Dairy type** | **Study excluded** | **Relative risk (95%CI) at 1 serving/d^2^** | **Lowest or highest relative risk (95%CI) for quadratic fit** |
|  |  |  |  |
| **Total dairy** |  |  |  |
| Lowest RR excluding: | HS-I | 0.84 (0.76-0.93) | 0.71 (0.57-0.88) at 3.4 servings/d |
| Highest RR excluding: | FHS-OC | 0.90 (0.83-0.98) | 0.82 (0.68-0.97) at 3.4 servings/d |
| **High-fat dairy** | |  |  |
| Lowest RR excluding: | Lifelines | 0.98 (0.94-1.02) |  |
| Highest RR excluding: | FHS-OC | 0.99 (0.96-1.02) |  |
| **Low-fat dairy** | |  |  |
| Lowest RR excluding: | Lifelines | 1.00 (0.91-1.11) | 0.84 (0.59-1.19) at 5.2 servings/d |
| Highest RR excluding: | RS-III | 0.99 (0.88-1.10) | 0.98 (0.87-1.11) at 2.6 servings/d |
| **Fermented dairy** | |  |  |
| Lowest RR excluding: | Fenland | 0.92 (0.85-1.00) | 0.89 (0.79-1.00) at 2.0 servings/d |
| Highest RR excluding: | Lifelines | 0.95 (0.86-1.05) | 0.93 (0.80-1.08) at 1.9 servings/d |
| **High-fat fermented dairy** | |  |  |
| Lowest RR excluding: | HS-II | 0.94 (0.87-1.02) | 0.93 (0.84-1.03) at 1.7 servings/d |
| Highest RR excluding: | HS-I | 0.96 (0.89-1.04) | 0.95 (0.87-1.05) at 1.6 servings/d |
| **Low-fat fermented dairy** | |  |  |
| Lowest RR excluding: | RS-I | 0.98 (0.94-1.03) |  |
| Highest RR excluding: | RS-III | 0.99 (0.95-1.04) |  |
| **Total milk** |  |  |  |
| Lowest RR excluding: | RS-I | 0.95 (0.87-1.05) | 0.92 (0.81-1.05) at 2.9 servings/d |
| Highest RR excluding: | Fenland | 0.99 (0.89-1.10) | 0.98 (0.85-1.13) at 2.6 servings/d |
| **High-fat milk** |  |  |  |
| Lowest RR excluding: | HS-I | 0.95 (0.84-1.08) |  |
| Highest RR excluding: | RS-II | 1.00 (0.92-1.09) |  |
| **Low-fat milk** |  |  |  |
| Lowest RR excluding: | RS-I | 1.01 (0.94-1.09) | 1.01 (0.94-1.09) at 1.0 servings/d |
| Highest RR excluding: | FHS-OC | 1.07 (1.01-1.13) | 1.07 (1.01-1.14) at 1.5 servings/d |
| **Total yogurt** |  |  |  |
| Lowest RR excluding: | HS-I | 0.95 (0.87-1.04) |  |
| Highest RR excluding: | RS-I | 1.01 (0.93-1.09) |  |
| **High-fat yogurt** | |  |  |
| Lowest RR excluding: | HS-I | 0.97 (0.75-1.28) |  |
| Highest RR excluding: | RS-I | 1.11 (0.94-1.31) |  |
| **Low-fat yogurt** | |  |  |
| Lowest RR excluding: | Lifelines | 0.98 (0.89-1.09) |  |
| Highest RR excluding: | Fenland | 1.02 (0.93-1.11) |  |
| **Total cheese** |  |  |  |
| Lowest RR excluding: | Lifelines | 0.88 (0.82-0.95) | 0.83 (0.74-0.93) at 2.3 servings/d |
| Highest RR excluding: | FHS-OC | 0.90 (0.85-0.97) | 0.87 (0.79-0.97) at 2.1 servings/d |
| **High-fat cheese** | |  |  |
| Lowest RR excluding: | Lifelines | 0.90 (0.84-0.97) | 0.86 (0.76-0.97) at 2.3 servings/d |
| Highest RR excluding: | HS-I | 0.95 (0.90-1.00) | 0.93 (0.87-1.01) at 1.8 servings/d |
| **Low-fat cheese** | |  |  |
| Lowest RR excluding: | HS-I | 1.02 (0.97-1.07) |  |
| Highest RR excluding: | HS-II | 1.07 (0.99-1.16) |  |
| **Cream** |  |  |  |
| Lowest RR excluding: | FHS-OC | 0.73 (0.52-1.02) |  |
| Highest RR excluding: | HS-II | 0.92 (0.85-1.00) |  |
| **Ice cream** |  |  |  |
| Lowest RR excluding: | RS-II | 0.46 (0.23-0.88) |  |
| Highest RR excluding: | HS-II | 0.53 (0.27-1.02) |  |
| ^1^ A one-stage meta-analysis was performed as the two-stage required at least two non-referent observations per individual study, thus limiting the assessment of study heterogeneity. ^2^ For composite dairy types, serving sizes were 200 g for liquid dairy foods and 20 g for solid dairy foods. For individual dairy types, serving sizes were 150 g for milk, yogurt, and ice cream; 20 g for cheese, and 15 g for cream. FHS-OC, Framingham Heart Study-Offspring Cohort; HS, Hoorn Study; RS, Rotterdam Study. | | | |

| **Supplemental table 9.** Goodness-of fit tests for models with potential moderators of the association between dairy foods and prediabetes risk based on one-stage linear or quadratic dose-response meta-regression.^1^ | | | | | | | |
| --- | --- | --- | --- | --- | --- | --- | --- |
| **Dairy type** | **Linear fit** | | |  | **Quadratic fit** | | |
|  | **Deviance** | **P-value** | **R^2^** |  | **Deviance** | **P-value** | **R^2^** |
| **Total dairy** |  |  |  |  |  |  |  |
| Model 4 | 37.8 | 0.048 | 0.04 |  | 26.8 | 0.31 | 0.32 |
| + follow-up duration | 35.1 | 0.067 | 1.06 |  | 25.4 | 0.33 | 0.35 |
| + year of dairy intake assessment | 37.1 | 0.043 | 0.06 |  | 26.7 | 0.27 | 0.32 |
| + prediabetes definition | 28.0 | 0.139 | 0.29 |  | 23.3 | 0.28 | 0.41 |
| **High-fat dairy** |  |  |  |  |  |  |  |
| Model 4 | 33.9 | 0.14 | 0.02 |  |  |  |  |
| + follow-up duration | 33.1 | 0.13 | 0.04 |  |  |  |  |
| + year of dairy intake assessment | 31.1 | 0.19 | 0.10 |  |  |  |  |
| + prediabetes definition | 25.6 | 0.27 | 0.26 |  |  |  |  |
| **Low-fat dairy** |  |  |  |  |  |  |  |
| Model 4 | 28.1 | 0.36 | 0.02 |  | 27.8 | 0.32 | 0.03 |
| + follow-up duration | 25.8 | 0.42 | 0.10 |  | 26.0 | 0.35 | 0.09 |
| + year of dairy intake assessment | 28.0 | 0.31 | 0.02 |  | 27.8 | 0.27 | 0.03 |
| + prediabetes definition | 18.8 | 0.67 | 0.34 |  | 21.9 | 0.41 | 0.23 |
| **Fermented dairy** |  |  |  |  |  |  |  |
| Model 4 | 19.2 | 0.63 | 0.01 |  | 15.0 | 0.82 | 0.22 |
| + follow-up duration | 19.1 | 0.58 | 0.01 |  | 15.0 | 0.78 | 0.22 |
| + year of dairy intake assessment | 18.1 | 0.64 | 0.06 |  | 14.9 | 0.78 | 0.23 |
| + prediabetes definition | 17.0 | 0.59 | 0.12 |  | 14.1 | 0.72 | 0.27 |
| **High-fat fermented dairy** |  |  |  |  |  |  |  |
| Model 4 | 10.8 | 0.95 | 0.03 |  | 9.4 | 0.97 | 0.15 |
| + follow-up duration | 10.8 | 0.93 | 0.03 |  | 9.3 | 0.95 | 0.16 |
| + year of dairy intake assessment | 10.7 | 0.93 | 0.04 |  | 9.0 | 0.96 | 0.19 |
| + prediabetes definition | 10.6 | 0.91 | 0.05 |  | 8.1 | 0.96 | 0.27 |
| **Low-fat fermented dairy** |  |  |  |  |  |  |  |
| Model 4 | 17.1 | 0.65 | 0.03 |  |  |  |  |
| + follow-up duration | 16.9 | 0.60 | 0.04 |  |  |  |  |
| + year of dairy intake assessment | 16.8 | 0.60 | 0.04 |  |  |  |  |
| + prediabetes definition | 17.1 | 0.52 | 0.03 |  |  |  |  |
| **Total milk** |  |  |  |  |  |  |  |
| Model 4 | 40.4 | 0.04 | 0.001 |  | 40.4 | 0.03 | 0.002 |
| + follow-up duration | 40.4 | 0.03 | 0.001 |  | 40.3 | 0.02 | 0.004 |
| + year of dairy intake assessment | 40.1 | 0.03 | 0.008 |  | 40.3 | 0.02 | 0.002 |
| + prediabetes definition | 33.1 | 0.08 | 0.18 |  | 33.5 | 0.04 | 0.17 |
| **High-fat milk** |  |  |  |  |  |  |  |
| Model 4 | 38.5 | 0.02 | 0.03 |  |  |  |  |
| + follow-up duration | 36.3 | 0.02 | 0.08 |  |  |  |  |
| + year of dairy intake assessment | 37.4 | 0.02 | 0.05 |  |  |  |  |
| + prediabetes definition | 26.6 | 0.09 | 0.33 |  |  |  |  |
| **Low-fat milk** |  |  |  |  |  |  |  |
| Model 4 | 39.5 | 0.04 | 0.003 |  | 36.9 | 0.06 | 0.07 |
| + follow-up duration | 39.3 | 0.04 | 0.01 |  | 36.5 | 0.05 | 0.08 |
| + year of dairy intake assessment | 39.5 | 0.03 | 0.004 |  | 36.0 | 0.06 | 0.09 |
| + prediabetes definition | 34.2 | 0.05 | 0.14 |  | 35.8 | 0.03 | 0.11 |
| **Total yogurt** |  |  |  |  |  |  |  |
| Model 4 | 38.1 | 0.05 | 0.002 |  |  |  |  |
| + follow-up duration | 36.2 | 0.05 | 0.05 |  |  |  |  |
| + year of dairy intake assessment | 37.9 | 0.04 | 0.008 |  |  |  |  |
| + prediabetes definition | 33.4 | 0.04 | 0.13 |  |  |  |  |
| **High-fat yogurt** |  |  |  |  |  |  |  |
| Model 4 | 23.9 | 0.25 | 0.02 |  |  |  |  |
| + follow-up duration | 20.7 | 0.35 | 0.15 |  |  |  |  |
| + year of dairy intake assessment | 23.9 | 0.20 | 0.02 |  |  |  |  |
| + prediabetes definition | 17.2 | 0.51 | 0.29 |  |  |  |  |
| **Low-fat yogurt** |  |  |  |  |  |  |  |
| Model 4 | 15.4 | 0.75 | 0.002 |  |  |  |  |
| + follow-up duration | 15.0 | 0.72 | 0.03 |  |  |  |  |
| + year of dairy intake assessment | 15.4 | 0.70 | 0.002 |  |  |  |  |
| + prediabetes definition | 14.5 | 0.69 | 0.06 |  |  |  |  |
| **Total cheese** |  |  |  |  |  |  |  |
| Model 4 | 27.8 | 0.27 | 0.004 |  | 15.2 | 0.90 | 0.46 |
| + follow-up duration | 27.7 | 0.23 | 0.01 |  | 14.8 | 0.87 | 0.47 |
| + year of dairy intake assessment | 26.6 | 0.27 | 0.05 |  | 15.0 | 0.98 | 0.46 |
| + prediabetes definition | 22.9 | 0.29 | 0.18 |  | 8.9 | 0.97 | 0.68 |
| **High-fat cheese** |  |  |  |  |  |  |  |
| Model 4 | 29.0 | 0.15 | 0.04 |  | 23.9 | 0.30 | 0.21 |
| + follow-up duration | 28.2 | 0.13 | 0.06 |  | 23.2 | 0.28 | 0.23 |
| + year of dairy intake assessment | 27.9 | 0.14 | 0.07 |  | 23.8 | 0.25 | 0.21 |
| + prediabetes definition | 25.1 | 0.16 | 0.17 |  | 19.0 | 0.40 | 0.37 |
| **Low-fat cheese** |  |  |  |  |  |  |  |
| Model 4 | 17.1 | 0.76 | 0.17 |  |  |  |  |
| + follow-up duration | 16.7 | 0.73 | 0.20 |  |  |  |  |
| + year of dairy intake assessment | 10.3 | 0.98 | 0.50 |  |  |  |  |
| + prediabetes definition | 14.5 | 0.75 | 0.30 |  |  |  |  |
| **Cream** |  |  |  |  |  |  |  |
| Model 4 | 23.7 | 0.42 | 0.16 |  |  |  |  |
| + follow-up duration | 19.4 | 0.62 | 0.31 |  |  |  |  |
| + year of dairy intake assessment | 21.2 | 0.51 | 0.25 |  |  |  |  |
| + prediabetes definition | 18.4 | 0.56 | 0.35 |  |  |  |  |
| **Ice cream** |  |  |  |  |  |  |  |
| Model 4 | 18.0 | 0.20 | 0.71 |  |  |  |  |
| + follow-up duration | 18.0 | 0.20 | 0.65 |  |  |  |  |
| + year of dairy intake assessment | 17.7 | 0.22 | 0.67 |  |  |  |  |
| + prediabetes definition | 17.8 | 0.21 | 0.54 |  |  |  |  |
| ^1^ A one-stage meta-analysis was performed as the two-stage required at least two non-referent observations per moderator. | | | | | | | |

| **Supplemental table 10.** Associations between dairy foods and prediabetes risk based on linear or quadratic two-stage random-effects dose-response meta-analysis for different confounder models.^1^ | | | | | |
| --- | --- | --- | --- | --- | --- |
| **Dairy type** | **Relative risk (95%CI) at 1 serving/d^2^** | **Lowest or highest relative risk (95%CI) for quadratic fit^2^** | **Heterogeneity** | | |
|  |  |  | **I^2^** | **Q test** | **p-value** |
| **Total dairy** |  |  |  |  |  |
| Model 1 | 0.84 (0.77-0.92) | 0.69 (0.55-0.85) at 3.8 servings/d | 24.9% | 21.3 | 0.17 |
| Model 2 | 0.86 (0.78-0.95) | 0.74 (0.60-0.90) at 3.5 servings/d | 17.4% | 19.4 | 0.25 |
| Model 3 | 0.88 (0.81-0.96) | 0.77 (0.65-0.93) at 3.4 servings/d | 12.3% | 18.3 | 0.31 |
| Model 4 | 0.87 (0.78-0.96) | 0.75 (0.60-0.93) at 3.4 servings/d | 18.0% | 19.5 | 0.24 |
| **High-fat dairy** |  |  |  |  |  |
| Model 1 | 0.97 (0.94-0.99) |  | 17.4% | 9.7 | 0.29 |
| Model 2 | 0.97 (0.95-1.01) |  | 40.7% | 13.5 | 0.10 |
| Model 3 | 0.98 (0.95-1.01) |  | 36.4% | 12.6 | 0.13 |
| Model 4 | 0.99 (0.96-1.02) |  | 36.8% | 12.7 | 0.12 |
| **Low-fat dairy** |  |  |  |  |  |
| Model 1 | 0.99 (0.91-1.09) | 0.99 (0.71-1.37) at 5.2 servings/d | 31.7% | 23.4 | 0.10 |
| Model 2 | 1.01 (0.94-1.09) | 1.01 (0.75-1.35) at 5.2 servings/d | 25.4% | 21.4 | 0.16 |
| Model 3 | 1.02 (0.95-1.10) | 1.02 (0.79-1.33) at 5.2 servings/d | 20.6% | 20.2 | 0.21 |
| Model 4 | 1.00 (0.91-1.09) | 0.93 (0.66-1.32) at 5.2 servings/d | 27.8% | 22.1 | 0.14 |
| **Fermented dairy** |  |  |  |  |  |
| Model 1 | 0.89 (0.82-0.96) | 0.84 (0.75-0.94) at 2.4 servings/d | 0% | 10.9 | 0.69 |
| Model 2 | 0.92 (0.85-0.99) | 0.88 (0.79-0.99) at 2.1 servings/d | 0% | 9.9 | 0.77 |
| Model 3 | 0.93 (0.86-1.01) | 0.91 (0.82-1.02) at 1.9 servings/d | 0% | 8.5 | 0.86 |
| Model 4 | 0.93 (0.86-1.01) | 0.91 (0.81-1.02) at 2.0 servings/d | 0% | 9.4 | 0.81 |
| **High-fat fermented dairy** | |  |  |  |  |
| Model 1 | 0.92 (0.86-0.99) | 0.90 (0.83-0.98) at 2.0 servings/d | 0% | 8.0 | 0.78 |
| Model 2 | 0.95 (0.89-1.02) | 0.94 (0.86-1.02) at 1.9 servings/d | 0% | 9.5 | 0.66 |
| Model 3 | 0.95 (0.89-1.02) | 0.94 (0.86-1.02) at 1.9 servings/d | 0% | 9.1 | 0.69 |
| Model 4 | 0.95 (0.88-1.02) | 0.94 (0.86-1.03) at 1.7 servings/d | 0% | 7.9 | 0.79 |
| **Low-fat fermented dairy** | |  |  |  |  |
| Model 1 | 0.98 (0.94-1.02) |  | 0% | 2.8 | 0.94 |
| Model 2 | 1.00 (0.96-1.04) |  | 0% | 2.2 | 0.90 |
| Model 3 | 1.01 (0.97-1.05) |  | 0% | 1.9 | 0.93 |
| Model 4 | 0.98 (0.94-1.03) |  | 0% | 1.8 | 0.94 |
| **Total milk** |  |  |  |  |  |
| Model 1 | 0.96 (0.87-1.07) | 0.94 (0.82-1.10) at 2.4 servings/d | 45.9% | 29.5 | 0.02 |
| Model 2 | 0.98 (0.88-1.08) | 0.97 (0.83-1.12) at 2.4 servings/d | 45.5% | 29.4 | 0.02 |
| Model 3 | 0.99 (0.91-1.09) | 0.98 (0.85-1.13) at 4.1 servings/d | 35.5% | 24.8 | 0.07 |
| Model 4 | 0.98 (0.88-1.08) | 0.96 (0.84-1.09) at 3.5 servings/d | 36.7% | 25.3 | 0.07 |
| **High-fat milk** |  |  |  |  |  |
| Model 1 | 0.98 (0.86-1.10) |  | 65.8% | 23.4 | 0.003 |
| Model 2 | 0.96 (0.85-1.08) |  | 62.2% | 21.2 | 0.007 |
| Model 3 | 0.94 (0.85-1.05) |  | 56.7% | 18.5 | 0.02 |
| Model 4 | 0.97 (0.88-1.08) |  | 58.5% | 19.3 | 0.01 |
| **Low-fat milk** |  |  |  |  |  |
| Model 1 | 1.03 (0.95-1.13) | 0.89 (0.75-1.04) at 4.1 servings/d | 46.3% | 29.8 | 0.02 |
| Model 2 | 1.05 (0.97-1.14) | 0.91 (0.77-1.07) at 4.1 servings/d | 43.6% | 28.3 | 0.03 |
| Model 3 | 1.06 (0.98-1.14) | 0.91 (0.77-1.08) at 4.1 servings/d | 38.8% | 26.1 | 0.05 |
| Model 4 | 1.03 (0.95-1.12) | 0.87 (0.73-1.04) at 4.1 servings/d | 30.0% | 22.8 | 0.12 |
| **Total yogurt** |  |  |  |  |  |
| Model 1 | 0.90 (0.81-0.99) |  | 31.4% | 11.7 | 0.17 |
| Model 2 | 0.93 (0.85-1.02) |  | 21.9% | 10.2 | 0.25 |
| Model 3 | 0.96 (0.88-1.05) |  | 5.5% | 8.5 | 0.39 |
| Model 4 | 0.98 (0.90-1.07) |  | 7.9% | 8.7 | 0.37 |
| **High-fat yogurt** |  |  |  |  |  |
| Model 1 | 0.86 (0.66-1.11) |  | 51.6% | 12.4 | 0.05 |
| Model 2 | 0.91 (0.70-1.18) |  | 48.5% | 11.6 | 0.07 |
| Model 3 | 0.92 (0.72-1.18) |  | 45.7% | 11.1 | 0.09 |
| Model 4 | 1.04 (0.87-1.25) |  | 21.0% | 7.6 | 0.27 |
| **Low-fat yogurt** |  |  |  |  |  |
| Model 1 | 0.96 (0.89-1.05) |  | 0% | 4.6 | 0.60 |
| Model 2 | 0.99 (0.91-1.08) |  | 0% | 5.3 | 0.50 |
| Model 3 | 1.01 (0.93-1.10) |  | 0% | 4.3 | 0.64 |
| Model 4 | 0.97 (0.82-1.15) |  | 0% | 2.9 | 0.83 |
| **Total cheese** |  |  |  |  |  |
| Model 1 | 0.89 (0.84-0.95) | 0.86 (0.78-0.94) at 2.1 servings/d | 0.0% | 13.0 | 0.67 |
| Model 2 | 0.91 (0.85-0.96) | 0.88 (0.80-0.96) at 2.1 servings/d | 0.0% | 12.3 | 0.72 |
| Model 3 | 0.92 (0.86-0.97) | 0.89 (0.82-0.98) at 1.9 servings/d | 0.0% | 11.6 | 0.77 |
| Model 4 | 0.89 (0.84-0.95) | 0.86 (0.78-0.94) at 2.1 servings/d | 0.0% | 12.6 | 0.70 |
| **High-fat cheese** |  |  |  |  |  |
| Model 1 | 0.92 (0.86-0.97) | 0.88 (0.81-0.97) at 2.2 servings/d | 21.2% | 17.8 | 0.22 |
| Model 2 | 0.92 (0.86-0.98) | 0.89 (0.80-0.99) at 2.2 servings/d | 24.1% | 18.4 | 0.19 |
| Model 3 | 0.93 (0.88-0.99) | 0.91 (0.83-1.00) at 2.1 servings/d | 9.2% | 15.4 | 0.35 |
| Model 4 | 0.92 (0.87-0.98) | 0.90 (0.81-0.99) at 2.1 servings/d | 11.6% | 15.8 | 0.32 |
| **Low-fat cheese** |  |  |  |  |  |
| Model 1 | 1.07 (0.99-1.17) |  | 57.0% | 16.3 | 0.03 |
| Model 2 | 1.08 (1.00-1.17) |  | 51.0% | 14.3 | 0.05 |
| Model 3 | 1.10 (1.02-1.19) |  | 51.6% | 14.5 | 0.04 |
| Model 4 | 1.05 (0.98-1.14) |  | 48.0% | 13.5 | 0.06 |
| **Cream** |  |  |  |  |  |
| Model 1 | 0.64 (0.40-1.02) |  | 69.9% | 23.2 | 0.002 |
| Model 2 | 0.83 (0.63-1.08) |  | 5.8% | 7.4 | 0.39 |
| Model 3 | 0.78 (0.58-1.03) |  | 16.6% | 8.4 | 0.30 |
| Model 4 | 0.85 (0.69-1.05) |  | 0.0% | 6.2 | 0.51 |
| **Ice cream** |  |  |  |  |  |
| Model 1 | 0.51 (0.27-0.96) |  | 0.0% | 1.99 | 0.96 |
| Model 2 | 0.63 (0.34-1.19) |  | 0.0% | 2.6 | 0.92 |
| Model 3 | 0.57 (0.30-1.08) |  | 0.0% | 2.1 | 0.95 |
| Model 4 | 0.50 (0.26-0.94) |  | 0.0% | 2.4 | 0.93 |
| ^1^ Relative risks (95%CIs) were adjusted as follows: Model 1 included age, sex, and energy intake. Model 2 additionally adjusted for educational level, smoking behaviour, physical activity, alcohol intake and family history of diabetes. Model 3 additionally adjusted for intake of food groups. Model 4 additionally adjusted for waist circumference or BMI, hypertension and dyslipidaemia. ^2^ For composite dairy types, serving sizes were 200 g for liquid dairy foods and 20 g for solid dairy foods. For individual dairy types, serving sizes were 150 g for milk, yogurt, and ice cream; 20 g for cheese, and 15 g for cream. | | | | | |

| **Supplemental table 11.** Prospective cohort studies reporting associations between dairy foods and glycemic outcomes | | | | | | | | | | | |  |
| --- | --- | --- | --- | --- | --- | --- | --- | --- | --- | --- | --- | --- |
| **Author, year** | **Cohort (follow-up), baseline and location** | **N, female %** | **Mean age, y** | **Mean BMI, kg/m2** | | **Dietary assessment** | **Analysis** | **Adjustments** | **Dairy type** | **Glycemic markers** | **Effect estimates, β (95%CI or ±SD)** | |
| Feskens, 1995 (156) | Seven Countries Study including East Finland, West Finland, and Zutphen cohort (20 y), 1958-1964, Finland and The Netherlands. | 338, 0% | 75.5^2^ | 26.0^2^ | | Cross-check dietary history method by experienced dieticians and nutritionist. | Baseline intake and change in intake in relation to outcome at follow-up. | Cohort, age, past BMI, and past energy intake. Change estimates were additionally adjusted for past consumption. | Baseline milk and milk products, g/1,000 kcal | 2hPG, mmol/l | 0.0002 ± 0.002 | |
|  |  |  |  |  |  |  |  |  | Change in milk and milk products, g/1,000 kcal | 2hPG, mmol/l | 0.001 ± 0.001 | |
| Ma, 2006 (157) | Insulin Resistance Atherosclerosis Study (IRAS) (5 y), 1992-1994, United States. | 1,087, 56.4% | 54.8 | 28.5 | | 114-item FFQ validated by comparison with 8 24-hour dietary recalls. | Repeated measures of intake in relation to repeated measures of outcome. | Age, sex, ethnicity, clinical center, energy intake, family history of diabetes, smoking, alcohol intake, PA, protein, fat, fiber, calcium, magnesium, grains, fruit, vegetables, fish, meat, and BMI. | Total dairy, servings/d | Log insulin sensitivity index | No statistically significantly associations (effect estimates reported in figure, p = 0.41). | |
| Snijder, 2008 (158) | Hoorn Study (6.4 y), 1989, The Netherlands. | 1,124, 46.1% | 60.0^2^ | 26.3 | | 92-item FFQ. Validated against dietary history, controlled feeding trials, and 24-h recalls. | Baseline intake in relation to change in outcome. | Age, sex, energy intake, baseline outcome value, alcohol intake, smoking, and PA. | Total dairy, servings/d | FPG, mmol/L | **0.026 ± 0.011** | |
|  |  |  |  |  |  |  |  |  | Total dairy, servings/d | 2hPG, mmol/L | 0.001 ± 0.028 | |
|  |  |  |  |  |  |  |  |  | High-fat-, low-fat dairy, plain milk, dairy desserts, yogurt and cheese, servings/d | FPG and 2hPG, mmol/L | No associations found, results not shown. | |
| Fumeron, 2011 (27) | DESIR (9 y), 2007-2010, France. | 3,417, 50.1% | 30-65 | 24.4^2^ | | 23-item FFQ. Validated by comparison with dietary history method. | Repeated measures of intake in relation to repeated measures of outcome. | Age, sex, alcohol, smoking, PA, fat intake and BMI. | Total dairy, servings/d | Insulin, pmol/l | **Intake, p = 0.02.**  Intake * time, p = 0.90. | |
|  |  |  |  |  |  |  |  |  | Total dairy (milk and other dairy products except cheese), servings/d | Insulin, pmol/l | Intake, p = 0.14.  Intake * time, p = 0.98. | |
|  |  |  |  |  |  |  |  |  | Cheese, servings/d | Insulin, pmol/l | Intake, p = 0.05.  Intake * time, p = 0.31. | |
| Struijk, 2012 (159) | Inter99 (5 y), 1999-2001, Denmark | 5,953, 47.5% | 45.8 | 26.1 | | 198-item FFQ. Validated against a 28-day dietary history with *r =* .55 for men and .60 for women, and for protein of .47 and .55, respectively. | Baseline intake in relation to outcome at follow-up. | Age, sex, intervention group, educational level, diabetes family history, PA, smoking, alcohol intake, wholegrain cereal, meat, fish, coffee, tea, fruit, vegetables, energy intake, change in diet quality score and waist circumference. | Total dairy, serving/d | FPG, mmol/L | -0.006 (-0.016; 0.004) | |
|  |  |  |  |  |  |  |  |  |  | 2hPG, mmol/L | 0.002 (-0.029; 0.032) | |
|  |  |  |  |  |  |  |  |  |  | HbA1c, % | -0.005 (-0.012; 0.002) | |
|  |  |  |  |  |  |  |  |  |  | HOMA2-IR, % | 0.002 (-0.008; 0.013) | |
|  |  |  |  |  |  |  |  |  |  | HOMA2-B % | 0.005 (-0.002; 0.012) | |
|  |  |  |  |  |  |  |  |  | High-fat dairy, serving/d | FPG, mmol/L | 0.002 (-0.024; 0.027) | |
|  |  |  |  |  |  |  |  |  |  | 2hPG, mmol/L | -0.027 (-0.108; 0.053) | |
|  |  |  |  |  |  |  |  |  |  | HbA1c, % | -0.014 (-0.033; 0.004) | |
|  |  |  |  |  |  |  |  |  |  | HOMA2-IR, % | 0.017 (-0.010; 0.043) | |
|  |  |  |  |  |  |  |  |  |  | HOMA2-B % | 0.012 (-0.006; 0.029) | |
|  |  |  |  |  |  |  |  |  | Low-fat dairy, serving/d | FPG, mmol/L | -0.006 (-0.016; 0.004) | |
|  |  |  |  |  |  |  |  |  |  | 2hPG, mmol/L | 0.006 (-0.025; 0.037) | |
|  |  |  |  |  |  |  |  |  |  | HbA1c, % | -0.003 (-0.01; 0.004) | |
|  |  |  |  |  |  |  |  |  |  | HOMA2-IR, % | -0.000 (-0.010; 0.010) | |
|  |  |  |  |  |  |  |  |  |  | HOMA2-B % | 0.004 (-0.003; 0.010) | |
|  |  |  |  |  |  |  |  |  | Milk and milk products, serving/d | FPG, mmol/L | -0.005 (-0.015; 0.005) | |
|  |  |  |  |  |  |  |  |  |  | 2hPG, mmol/L | 0.005 (-0.026; 0.035) | |
|  |  |  |  |  |  |  |  |  |  | HbA1c, % | -0.005 (-0.012; 0.002) | |
|  |  |  |  |  |  |  |  |  |  | HOMA2-IR, % | 0.003 (-0.008; 0.013) | |
|  |  |  |  |  |  |  |  |  |  | HOMA2-B % | 0.005 (-0.002; 0.012) | |
|  |  |  |  |  |  |  |  |  | Cheese, serving/d | FPG, mmol/L | -0.009 (-0.024; 0.006) | |
|  |  |  |  |  |  |  |  |  |  | 2hPG, mmol/L | **-0.048 (-0.095; -0.001)** | |
|  |  |  |  |  |  |  |  |  |  | HbA1c, % | 0.005 (-0.006; 0.016) | |
|  |  |  |  |  |  |  |  |  |  | HOMA2-IR, % | -0.005 (-0.021; 0.010) | |
|  |  |  |  |  |  |  |  |  |  | HOMA2-B % | 0.000 (-0.010; 0.010) | |
|  |  |  |  |  |  |  |  |  | Fermented dairy, serving/d | FPG, mmol/L | **-0.028 (-0.048; -0.008)** | |
|  |  |  |  |  |  |  |  |  |  | 2hPG, mmol/L | -0.053 (-0.115; 0.010) | |
|  |  |  |  |  |  |  |  |  |  | HbA1c, % | **-0.016 (-0.030; -0.001)** | |
|  |  |  |  |  |  |  |  |  |  | HOMA2-IR, % | -0.010 (-0.03; 0.011) | |
|  |  |  |  |  |  |  |  |  |  | HOMA2-B % | 0.006 (-0.007; 0.020) | |
| Samara, 2013 (160) | STANISLAS study (5 y), 1994-1995, France | 288 men, 300 women | 42.8; 40.9 | 25.4; 23.5 | | 3-day dietary records. | Baseline intake in relation to change in outcome. | Age, PA, alcohol, smoking, energy intake, education level, mean adequacy ratio index, and baseline outcome value. | *Men* |  |  | |
|  |  |  |  |  |  |  |  |  | Milk, yogurt, cottage cheese, servings/MJ | Fasting glucose, mmol/L | **-0.199 ± 0.070** | |
|  |  |  |  |  |  |  |  |  | Cheese, servings/MJ |  | -0.064 ± 0.064 | |
|  |  |  |  |  |  |  |  |  | *Women* |  |  | |
|  |  |  |  |  |  |  |  |  | Milk, yogurt, cottage cheese, servings/MJ | Fasting glucose, mmol/L | -0.010 ± 0.034 | |
|  |  |  |  |  |  |  |  |  | Cheese, servings/MJ |  | -0.061 ± 0.037 | |
| Panahi, 2018 (161) | Quebec Family Study (6 y), 1978, Canada. | 248, 53.7 | 40.5^2^ | 27.9^2^ | | 3-day dietary records. | Repeated measures of intake in relation to repeated measures of outcome. | Age, diet quality, physical activity, and % body fat. | Total yogurt, consumers or non-consumers | *Men* |  | |
|  |  |  |  |  | |  |  |  |  | FPG, mmol/L | **Group, p = 0.07**  **Time, p = 0.0004**  Interaction, p = 0.26 | |
|  |  |  |  |  | |  |  |  |  | Fasting insulin, pmol/L | **Group, p = 0.005**  Time, p = 0.13  Interaction, p = 0.53 | |
|  |  |  |  |  | |  |  |  |  | Fasting C-peptide, mmol/L | **Group, p = 0.004**  **Time, p < 0.0001**  Interaction, p = 0.83 | |
|  |  |  |  |  | |  |  |  |  | Glucose AUC, min mmol/L | Group, p = 0.13  **Time, p = 0.0008**  Interaction, p = 0.78 | |
|  |  |  |  |  | |  |  |  |  | Insulin AUC, min pmol/L | **Group, p = 0.008**  Time, p = 0.19  Interaction, p = 0.43 | |
|  |  |  |  |  | |  |  |  |  | *Women* |  | |
|  |  |  |  |  | |  |  |  |  | FPG, mmol/L | Group, p = 0.07  **Time, p < 0.0001**  Interaction, p = 0.21 | |
|  |  |  |  |  | |  |  |  |  | Fasting insulin, pmol/L | **Group, p = 0.03**  **Time, p = 0.12**  Interaction, p = 0.83 | |
|  |  |  |  |  | |  |  |  |  | Fasting C-peptide, mmol/L | Group, p = 0.14  **Time, p < 0.0001**  Interaction, p = 0.73 | |
|  |  |  |  |  | |  |  |  |  | Glucose AUC, min mmol/L | **Group, p = 0.003**  **Time, p = 0.02**  Interaction, p = 0.34 | |
|  |  |  |  |  | |  |  |  |  | Insulin AUC, min pmol/L | Group, p = 0.12  Time, p = 0.11  Interaction, p = 0.50 | |
| Huang, 2019 (162) | Meta-analysis of 18 studies (1 to 26 years, 6 studies without follow-up), United States, Denmark, Spain, Australia and Finland. | 182,041, 48.8%-64% | 19.9-70.9 | 24.3-30.1 | | FFQ in 14 studies. General questionnaire in 2 studies. 66-item interviewer administered in 1 study. | Baseline intake in relation to outcome at follow-up. | Sex, ethnicity, region, years of follow-up, and other baseline covariates if available (age, smoking status, physical activity, total energy intake, and alcohol intake). | Total dairy, serving/d | FPG, mmol/L | 0.004 ± 0.002, I^2^ = 16% | |
|  |  |  |  |  |  |  |  |  |  | HbA1c, % | **0.009 ± 0.002**, I^2^ = 9% | |
|  |  |  |  |  |  |  |  |  |  | Log fasting insulin, mIU/L | 0.001 ± 0.006, I^2^ = 8% | |
|  |  |  |  |  |  |  |  |  |  | Log HOMA-IR | 0.006 ± 0.007, I^2^ = 3% | |
|  |  |  |  |  |  |  |  |  |  | Log HOMA-β | 0.007 ± 0.010, I^2^ = 2% | |
| Trichia, 2020 (163) | EPIC-Norfolk study (3.7 y), 1993-1997, United Kingdom. | 6,224, 56.2% | 58.6 | 26.1 | | 130-item FFQ. Validated against 7-d food diaries, *r* = 0.56 for milk, 0.57 for yogurt, 0.33 for cheese, and 0.54 for butter. | Change intake in relation to change in outcome, and repeated measures of intake in relation to repeated measures of outcome. | Age, sex, educational level, age at completion of full-time education, marital status, occupation, follow-up time, PA, smoking, medication, energy intake, fruit, vegetables, potatoes, legumes, nuts, processed cereals, wholegrain cereals, poultry and eggs, red meat, processed meat, fish, sauces, margarine, sweet snacks, SSBs, ASBs, fruit juice, coffee, tea and alcoholic beverages, supplement use and BMI. | *Change in intake* |  |  | |
|  |  |  |  |  |  |  |  |  | Total dairy, serving/d | HbA1c, mmol/mol | 0.03 (-0.12, 0.17) | |
|  |  |  |  |  |  |  |  |  | High-fat dairy, serving/d |  | 0.04 (-0.17, 0.25) | |
|  |  |  |  |  |  |  |  |  | Low-fat dairy, serving/d |  | 0.06 (-0.12, 0.25) | |
|  |  |  |  |  |  |  |  |  | Fermented dairy, serving/d |  | 0.04 (-0.32, 0.39) | |
|  |  |  |  |  |  |  |  |  | Milk, serving/d |  | 0.10 (-0.14, 0.34) | |
|  |  |  |  |  |  |  |  |  | Full-fat milk, serving/d |  | **0.52 (0.06, 0.97)** | |
|  |  |  |  |  |  |  |  |  | Low-fat milk, serving/d |  | 0.04 (-0.23, 0.31) | |
|  |  |  |  |  |  |  |  |  | Yogurt, serving/d |  | 0.21 (-0.29, 0.71) | |
|  |  |  |  |  |  |  |  |  | Full-fat yogurt, serving/d |  | -0.01 (-1.68, 1.66) | |
|  |  |  |  |  |  |  |  |  | Low-fat yogurt, serving/d |  | 0.33 (-0.23, 0.89) | |
|  |  |  |  |  |  |  |  |  | Cheese, serving/d |  | -0.04 (-0.60, 0.52) | |
|  |  |  |  |  |  |  |  |  | High-fat cheese, serving/d |  | -0.11 (-0.81, 0.60) | |
|  |  |  |  |  | |  |  |  | Low-fat cheese, serving/d |  | 0.02 (-0.86, 0.90) | |
|  |  |  |  |  | |  |  |  | Ice-cream, serving/d |  | 0.14 (-0.55, 0.84) | |
|  |  |  |  |  | |  |  |  | *Repeated measurements* |  |  | |
|  |  |  |  |  | |  |  |  | Total dairy, serving/d | HbA1c, mmol/mol | -0.05 (-0.15; 0.05) | |
|  |  |  |  |  | |  |  |  | High-fat dairy, serving/d |  | -0.09 (-0.20; 0.03) | |
|  |  |  |  |  | |  |  |  | Low-fat dairy, serving/d |  | -0.04 (-0.16; 0.07) | |
|  |  |  |  |  | |  |  |  | Fermented dairy, serving/d |  | -0.12 (-0.31; 0.06) | |
|  |  |  |  |  | |  |  |  | Milk, serving/d |  | -0.05 (-0.19; 0.09) | |
|  |  |  |  |  | |  |  |  | Full-fat milk, serving/d |  | **-0.21 (-0.41; -0.01)** | |
|  |  |  |  |  | |  |  |  | Low-fat milk, serving/d |  | -0.04 (-0.18; 0.10) | |
|  |  |  |  |  | |  |  |  | Yogurt, serving/d |  | 0.00 (-0.24; 0.24) | |
|  |  |  |  |  | |  |  |  | Full-fat yogurt, serving/d |  | -0.03 (-0.77; 0.71) | |
|  |  |  |  |  | |  |  |  | Low-fat yogurt, serving/d |  | 0.00 (-0.25; 0.25) | |
|  |  |  |  |  | |  |  |  | Cheese, serving/d |  | **-0.31 (-0.59; -0.03)** | |
|  |  |  |  |  | |  |  |  | High-fat cheese, serving/d |  | **-0.63 (-1.01; -0.25)** | |
|  |  |  |  |  | |  |  |  | Low-fat cheese, serving/d |  | -0.02 (-0.40; 0.36) | |
|  |  |  |  |  | |  |  |  | Ice-cream, serving/d |  | -0.14 (-0.51; 0.23) | |
| Riseberg, 2022 (164) | Boston Puerto Rican Health Study (2 y), 2004-2007, United States. |  |  |  | | FFQ validated against vitamins E and B12 and plasma carotenoids. | Baseline intake in relation to outcome at follow-up. | Energy intake, sex, age, education, baseline outcome, smoking, alcohol intake, PA, psychological acculturation, fruit and vegetable intake score, omega-3 fatty acid intake, wholegrain intake, and medication. | Milk and yogurt, serving/d | FPG, mg/dL | 0.91 (−1.27, 3.09) | |
|  |  |  |  |  | |  |  |  | Cheese, serving/d |  | −0.45 (−5.51, 4.62) | |
| Slurink, 2022 (149) | Rotterdam Study, I (20.9 y), 2000  Rotterdam Study, II (12.6 y), 2006  Rotterdam Study, III (7 y), 2006-2013, the Netherlands. |  |  |  | | *See table 1.* | Baseline intake in relation to repeated measures of outcome. | Age, sex, energy intake, education, smoking, physical activity, alcohol intake, family history of diabetes (not in RS-III), fruit, vegetables, whole grains, legumes, nuts, tea, coffee, red meat and SSBs. Longitudinal waist circumference in sensitivity analyses. | Total dairy, serving/d | Log HOMA-IR | 0.00 (0.00, 0.01) | |
|  |  |  |  |  | |  |  |  | High-fat dairy, serving/d |  | -0.01 (-0.02, 0.00) | |
|  |  |  |  |  | |  |  |  | Low-fat dairy, serving/d |  | **0.02 (0.01, 0.03)** | |
|  |  |  |  |  | |  |  |  | Fermented dairy, serving/d |  | 0.00 (-0.01, 0.00) | |
|  |  |  |  |  | |  |  |  | High-fat fermented dairy, serving/d |  | 0.00 (-0.01, 0.01) | |
|  |  |  |  |  | |  |  |  | Low-fat fermented dairy, serving/d |  | 0.00 (-0.01, 0.01) | |
|  |  |  |  |  | |  |  |  | Milk, serving/d |  | 0.01 (0.00, 0.02) | |
|  |  |  |  |  | |  |  |  | High-fat milk, serving/d |  | -0.02 (-0.04, 0.00) | |
|  |  |  |  |  | |  |  |  | Low-fat milk, serving/d |  | **0.02 (0.01, 0.04)** | |
|  |  |  |  |  | |  |  |  | Yogurt, serving/d |  | -0.02 (-0.04, 0.00) | |
|  |  |  |  |  | |  |  |  | High-fat yogurt, serving/d |  | **-0.08 (-0.13, -0.03)** | |
|  |  |  |  |  | |  |  |  | Low-fat yogurt, serving/d |  | 0.00 (-0.03, 0.02) | |
|  |  |  |  |  | |  |  |  | Cheese, serving/d |  | 0.00 (-0.01, 0.01) | |
|  |  |  |  |  | |  |  |  | High-fat cheese, serving/d |  | 0.00 (-0.01, 0.01) | |
|  |  |  |  |  | |  |  |  | Low-fat cheese, serving/d |  | 0.01 (-0.01, 0.03) | |
|  |  |  |  |  | |  |  |  | Cream, serving/ |  | -0.02 (-0.05, 0.01) | |
|  |  |  |  |  | |  |  |  | Ice-cream, serving/d |  | 0.04 (-0.01, 0.08) | |
| Yun, 2022 (165) | NHAPC (Nutrition and Health of Aging Population in China) (6 y), 2005, China | 2,140, 59.4% | 58.4 | 24.4 | | 74-item FFQ. Validated against dietary records, *r* ranging from .68 to .72. | Baseline intake in relation to changes in outcome. | Age, sex, region, residence, educational attainment, smoking, alcohol intake, PA, family history of chronic disease, lipid-lowering medication, energy intake, red meat, egg, fish, soy milk, vegetables, fruit, fiber, baseline outcome value. | Total dairy | Change in FPG, mmol/L | **-0.14 (-0.21, -0.07)** | |
|  |  |  |  |  | |  |  |  | Milk |  | **-0.12 (-0.18, -0.05)** | |
|  |  |  |  |  | |  |  |  | Non-milk dairy products |  | -0.18 (-0.40, 0.03) | |
| Chatzidiakou, 2023 (166) (abstract only) | Caerphilly prospective cohort study (5 y), United Kingdom | 1,350, 0% | 45-59 | NR | | 50-item FFQ. Validated against 7-day weighted dietary intake, *r =* .3 to .5 for all food items. | Baseline intake in relation to changes in outcome. | Age, BMI, total energy intake and baseline outcome value. | Total dairy Milk Cheese Cream | Changes in FPG, insulin and IR estimates. | No associations were observed (effect estimates not reported). | |
| Slurink, 2024 (151) | Fenland Study (6.7 y), 2005-2015, United Kingdom | 7,410, 51.9% | 48.7 | 26.4 | | *See table 1.* | Change intake in relation to change in outcome, and repeated measures of intake in relation to repeated measures of outcome. | Age, sex, study site, (change in) energy intake, educational level, age at completion of education, ethnic origin, (change in) alcohol intake, smoking, (change in) PA, family history of diabetes, (change in) fruit, vegetables, whole grains, refined grains, potatoes, legumes, nuts, red and processed meat, fatty fish, coffee, tea, SSBs, hypertension, dyslipidemia and (change in) waist circumference and baseline outcome value. | *Change in intake* |  |  | |
|  |  |  |  |  | |  |  |  | Total dairy, serving/d | FPG, mmol/L | 0.01 (-0.01, 0.03) | |
|  |  |  |  |  | |  |  |  |  | 2hPG, mmol/L | 0.02 (-0.01, 0.06) | |
|  |  |  |  |  | |  |  |  |  | HbA1c, % | 0.01 (0.00, 0.02) | |
|  |  |  |  |  | |  |  |  | High-fat dairy, serving/d | FPG, mmol/L | -0.01 (-0.03, 0.01) | |
|  |  |  |  |  | |  |  |  |  | 2hPG, mmol/L | -0.03 (-0.08, 0.03) | |
|  |  |  |  |  | |  |  |  |  | HbA1c, % | 0.00 (-0.01, 0.01) | |
|  |  |  |  |  | |  |  |  | Low-fat dairy, serving/d | FPG, mmol/L | **0.02 (0.00, 0.04)** | |
|  |  |  |  |  | |  |  |  |  | 2hPG, mmol/L | **0.04 (0.00, 0.08)** | |
|  |  |  |  |  | |  |  |  |  | HbA1c, % | 0.01 (0.00, 0.02) | |
|  |  |  |  |  | |  |  |  | Fermented dairy, serving/d | FPG, mmol/L | 0.00 (-0.03, 0.02) | |
|  |  |  |  |  | |  |  |  |  | 2hPG, mmol/L | 0.00 (-0.05, 0.06) | |
|  |  |  |  |  | |  |  |  |  | HbA1c, % | 0.00 (-0.01, 0.01) | |
|  |  |  |  |  | |  |  |  | High-fat fermented dairy, serving/d | FPG, mmol/L | 0.00 (-0.03, 0.04) | |
|  |  |  |  |  | |  |  |  |  | 2hPG, mmol/L | -0.01 (-0.09, 0.07) | |
|  |  |  |  |  | |  |  |  |  | HbA1c, % | 0.01 (-0.01, 0.03) | |
|  |  |  |  |  | |  |  |  | Low-fat fermented dairy, serving/d | FPG, mmol/L | -0.01 (-0.03, 0.02) | |
|  |  |  |  | |  | |  |  |  | 2hPG, mmol/L | 0.00 (-0.06, 0.07) | |
|  |  |  |  |  | |  |  |  |  | HbA1c, % | 0.00 (-0.02, 0.01) | |
|  |  |  |  |  | |  |  |  | Milk, serving/d | FPG, mmol/L | 0.02 (0.00, 0.04) | |
|  |  |  |  |  | |  |  |  |  | 2hPG, mmol/L | 0.04 (0.00, 0.09) | |
|  |  |  |  |  | |  |  |  |  | HbA1c, % | 0.01 (0.00, 0.02) | |
|  |  |  |  |  | |  |  |  | High-fat milk, serving/d | FPG, mmol/L | **-0.03 (-0.05, 0.00** | |
|  |  |  |  |  | |  |  |  |  | 2hPG, mmol/L | -0.04 (-0.11, 0.03) | |
|  |  |  |  |  | |  |  |  |  | HbA1c, % | -0.01 (-0.03, 0.01) | |
|  |  |  |  |  | |  |  |  | Low-fat milk, serving/d | FPG, mmol/L | **0.03 (0.01, 0.05)** | |
|  |  |  |  |  | |  |  |  |  | 2hPG, mmol/L | **0.06 (0.01, 0.11)** | |
|  |  |  |  |  | |  |  |  |  | HbA1c, % | 0.01 (0.00, 0.02) | |
|  |  |  |  |  | |  |  |  | Yogurt, serving/d | FPG, mmol/L | 0.00 (-0.03, 0.04) | |
|  |  |  |  |  | |  |  |  |  | 2hPG, mmol/L | 0.01 (-0.07, 0.09) | |
|  |  |  |  |  | |  |  |  |  | HbA1c, % | 0.00 (-0.02, 0.02) | |
|  |  |  |  |  | |  |  |  | High-fat yogurt, serving/d | FPG, mmol/L | 0.01 (-0.04, 0.06) | |
|  |  |  |  |  | |  |  |  |  | 2hPG, mmol/L | 0.01 (-0.12, 0.14) | |
|  |  |  |  |  | |  |  |  |  | HbA1c, % | 0.00 (-0.03, 0.03) | |
|  |  |  |  |  | |  |  |  | Low-fat yogurt, serving/d | FPG, mmol/L | 0.00 (-0.03, 0.03) | |
|  |  |  |  |  | |  |  |  |  | 2hPG, mmol/L | 0.03 (-0.05, 0.11) | |
|  |  |  |  |  | |  |  |  |  | HbA1c, % | 0.00 (-0.02, 0.02) | |
|  |  |  |  |  | |  |  |  | Cheese, serving/d | FPG, mmol/L | -0.01 (-0.04, 0.02) | |
|  |  |  |  |  | |  |  |  |  | 2hPG, mmol/L | -0.01 (-0.08, 0.06) | |
|  |  |  |  |  | |  |  |  |  | HbA1c, % | 0.00 (-0.02, 0.01) | |
|  |  |  |  |  | |  |  |  | High-fat cheese, serving/d | FPG, mmol/L | 0.00 (-0.03, 0.04) | |
|  |  |  |  |  | |  |  |  |  | 2hPG, mmol/L | -0.02 (-0.11, 0.07) | |
|  |  |  |  |  | |  |  |  |  | HbA1c, % | 0.01 (-0.01, 0.03) | |
|  |  |  |  |  | |  |  |  | Low-fat cheese, serving/d | FPG, mmol/L | -0.02 (-0.08, 0.04) | |
|  |  |  |  |  | |  |  |  |  | 2hPG, mmol/L | 0.00 (-0.11, 0.10) | |
|  |  |  |  |  | |  |  |  |  | HbA1c, % | -0.01 (-0.04-0.01) | |
|  |  |  |  |  | |  |  |  | Cream, serving/ | FPG, mmol/L | 0.06 (-0.01, 0.12) | |
|  |  |  |  |  | |  |  |  |  | 2hPG, mmol/L | 0.00 (-0.13, 0.13) | |
|  |  |  |  |  | |  |  |  |  | HbA1c, % | 0.03 (-0.01, 0.06) | |
|  |  |  |  |  | |  |  |  | Ice-cream, serving/d | FPG, mmol/L | 0.08 (-0.10, 0.26) | |
|  |  |  |  |  | |  |  |  |  | 2hPG, mmol/L | 0.13 (-0.26, 0.53) | |
|  |  |  |  |  | |  |  |  |  | HbA1c, % | 0.01 (-0.08, 0.09) | |
|  |  |  |  |  | |  |  |  | *Repeated measurements* |  |  | |
|  |  |  |  |  | |  |  |  | Total dairy, serving/d | FPG, mmol/L | **0.002 (0.0, 0.004)** | |
|  |  |  |  |  | |  |  |  |  | 2hPG, mmol/L | 0.003 (-0.002, 0.008) | |
|  |  |  |  |  | |  |  |  |  | HbA1c, % | 0.0006 (-0.0004, 0.002) | |
|  |  |  |  |  | |  |  |  | High-fat dairy, serving/d | FPG, mmol/L | 0.002 (-0.0003, 0.005) | |
|  |  |  |  |  | |  |  |  |  | 2hPG, mmol/L | 0.006 (-0.002, 0.01) | |
|  |  |  |  |  | |  |  |  |  | HbA1c, % | **-0.002 (-0.003, -0.0001)** | |
|  |  |  |  |  | |  |  |  | Low-fat dairy, serving/d | FPG, mmol/L | 0.001 (-0.001, 0.003) | |
|  |  |  |  |  | |  |  |  |  | 2hPG, mmol/L | 0.002 (-0.004, 0.007) | |
|  |  |  |  |  | |  |  |  |  | HbA1c, % | **0.002 (0.0005, 0.003)** | |
|  |  |  |  |  | |  |  |  | Fermented dairy, serving/d | FPG, mmol/L | -0.0003 (-0.003, 0.003) | |
|  |  |  |  |  | |  |  |  |  | 2hPG, mmol/L | **0.01 (0.001, 0.02)** | |
|  |  |  |  |  | |  |  |  |  | HbA1c, % | 0.0 (-0.002, 0.002) | |
|  |  |  |  |  | |  |  |  | High-fat fermented dairy, serving/d | FPG, mmol/L | 0.003 (-0.002, 0.007) | |
|  |  |  |  |  | |  |  |  |  | 2hPG, mmol/L | **0.01 (0.001, 0.03)** | |
|  |  |  |  |  | |  |  |  |  | HbA1c, % | -0.002 (-0.004, 0.0009) | |
|  |  |  |  |  | |  |  |  | Low-fat fermented dairy, serving/d | FPG, mmol/L | -0.002 (-0.006, 0.002) | |
|  |  |  |  |  | |  |  |  |  | 2hPG, mmol/L | 0.006 (-0.004, 0.02) | |
|  |  |  |  |  | |  |  |  |  | HbA1c, % | 0.001 (-0.001, 0.003) | |
|  |  |  |  |  | |  |  |  | Milk, serving/d | FPG, mmol/L | **0.003 (0.001, 0.005)** | |
|  |  |  |  |  | |  |  |  |  | 2hPG, mmol/L | 0.0001 (-0.007, 0.007) | |
|  |  |  |  |  | |  |  |  |  | HbA1c, % | 0.0007 (-0.0006, 0.002) | |
|  |  |  |  |  | |  |  |  | High-fat milk, serving/d | FPG, mmol/L | 0.001 (-0.003, 0.005) | |
|  |  |  |  |  | |  |  |  |  | 2hPG, mmol/L | 0.003 (-0.009, 0.014) | |
|  |  |  |  |  | |  |  |  |  | HbA1c, % | **-0.004 (-0.006, -0.001)** | |
|  |  |  |  |  | |  |  |  | Low-fat milk, serving/d | FPG, mmol/L | **0.003 (0.0003, 0.005)** | |
|  |  |  |  |  | |  |  |  |  | 2hPG, mmol/L | -0.0003 (-0.007, 0.007) | |
|  |  |  |  |  | |  |  |  |  | HbA1c, % | **0.002 (0.001, 0.003)** | |
|  |  |  |  |  | |  |  |  | Yogurt, serving/d | FPG, mmol/L | -0.003 (-0.007, 0.001) | |
|  |  |  |  |  | |  |  |  |  | 2hPG, mmol/L | 0.01 (-0.002, 0.021) | |
|  |  |  |  |  | |  |  |  |  | HbA1c, % | -0.0005 (-0.003, 0.002) | |
|  |  |  |  |  | |  |  |  | High-fat yogurt, serving/d | FPG, mmol/L | -0.002 (-0.01, 0.008) | |
|  |  |  |  |  | |  |  |  |  | 2hPG, mmol/L | 0.01 (-0.02, 0.04) | |
|  |  |  |  |  | |  |  |  |  | HbA1c, % | -0.005 (-0.01, 0.0007) | |
|  |  |  |  |  | |  |  |  | Low-fat yogurt, serving/d | FPG, mmol/L | -0.003 (-0.007, 0.002) | |
|  |  |  |  |  | |  |  |  |  | 2hPG, mmol/L | 0.008 (-0.004, 0.02) | |
|  |  |  |  |  | |  |  |  |  | HbA1c, % | 0.0007 (-0.002, 0.003) | |
|  |  |  |  |  | |  |  |  | Cheese, serving/d | FPG, mmol/L | 0.003 (-0.002, 0.008) | |
|  |  |  |  |  | |  |  |  |  | 2hPG, mmol/L | 0.01 (-0.002, 0.02) | |
|  |  |  |  |  | |  |  |  |  | HbA1c, % | 0.0006 (-0.002, 0.003) | |
|  |  |  |  |  | |  |  |  | High-fat cheese, serving/d | FPG, mmol/L | 0.005 (-0.001, 0.01) | |
|  |  |  |  |  | |  |  |  |  | 2hPG, mmol/L | 0.016 (-0.0004, 0.03) | |
|  |  |  |  |  | |  |  |  |  | HbA1c, % | -0.0004 (-0.004, 0.003) | |
|  |  |  |  |  | |  |  |  | Low-fat cheese, serving/d | FPG, mmol/L | 0-0.001 (-0.009, 0.007) | |
|  |  |  |  |  | |  |  |  |  | 2hPG, mmol/L | 0.003 (-0.02, 0.03) | |
|  |  |  |  |  | |  |  |  |  | HbA1c, % | 0.003 (-0.002, 0.007) | |
|  |  |  |  |  | |  |  |  | Cream, serving/ | FPG, mmol/L | 0.007 (-0.004, 0.018) | |
|  |  |  |  |  | |  |  |  |  | 2hPG, mmol/L | 0.006 (-0.02, 0.04) | |
|  |  |  |  |  | |  |  |  |  | HbA1c, % | 0.002 (-0.004, 0.008) | |
|  |  |  |  |  | |  |  |  | Ice-cream, serving/d | FPG, mmol/L | 0.02 (-0.007, 0.04) | |
|  |  |  |  |  | |  |  |  |  | 2hPG, mmol/L | -0.009 (-0.07, 0.06) | |
|  |  |  |  |  | |  |  |  |  | HbA1c, % | **0.02 (0.003, 0.03)** | |
| ^1^ The dairy types are shown according to definitions used by the original articles. ^2^ Pooled across categories. Abbreviations: 2hPG, 2-hour Postprandial Glucose; ASBs: Artificially Sweetened Beverages; AUC: Area Under the Curve; BMI: Body Mass Index; EPIC, European Prospective Investigation into Cancer and Nutrition; FFQ, Food Frequency Questionnaire; FPG, Fasting Plasma Glucose; HbA1c, Hemoglobin A1c; HOMA-2B, Homeostatic Model Assessment of Beta-cell Function; HOMA-β, Homeostatic Model Assessment of Beta-cell Function; HOMA-IR, Homeostatic Model Assessment of Insulin Resistance; HOMA2-IR, Homeostatic Model Assessment of Insulin Resistance; IR, Insulin Resistance; METS, Metabolic Equivalents; NR, Not Reported; PA, Physical Activity; SFA, saturated fatty acids; SSBs, Sugar-Sweetened Beverages. | | | | | | | | | | | |  |


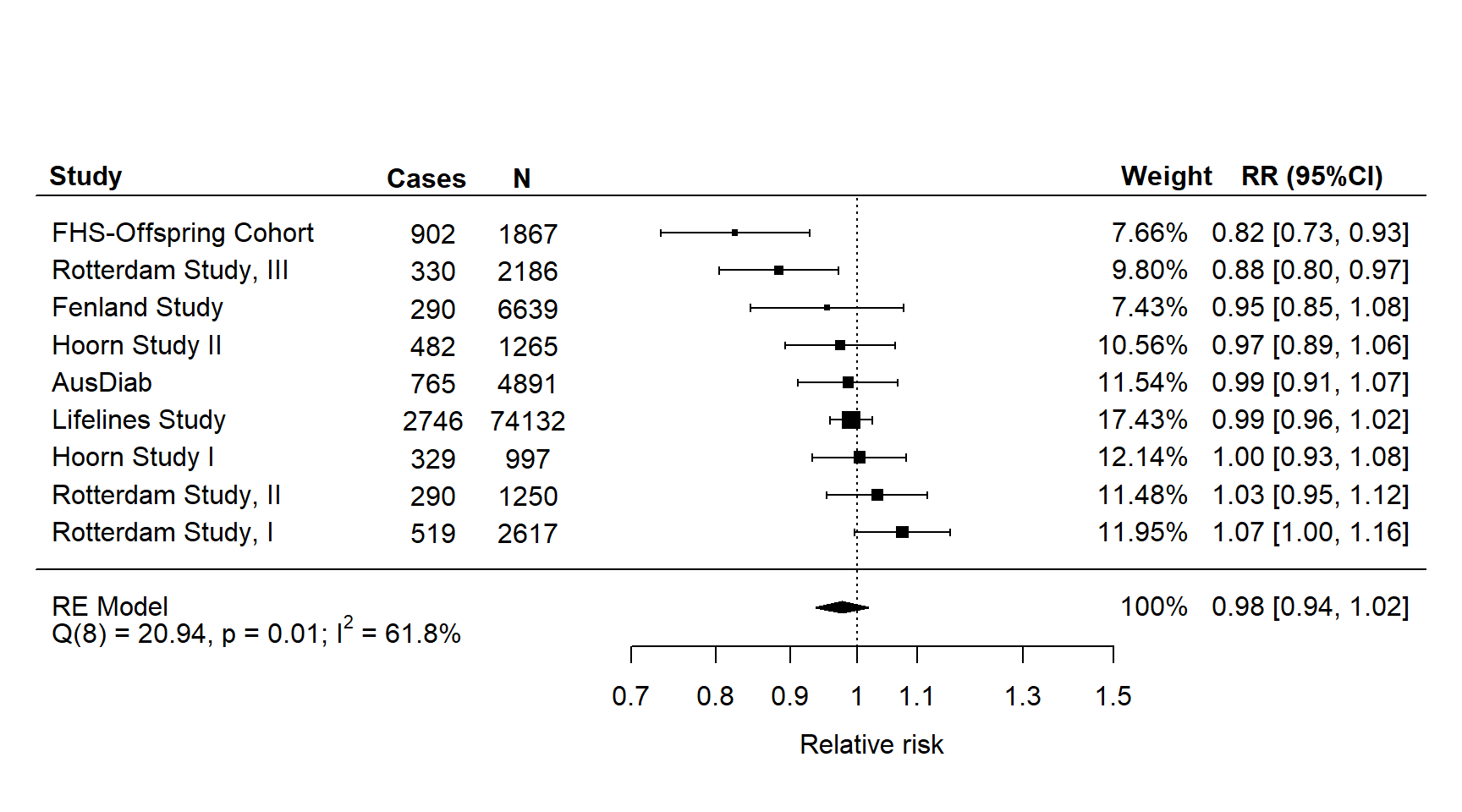


**Supplemental Figure 1.** Forest plot for the association between total dairy intake (per increment of 1 serving/day, with serving sizes defined as 200 g for liquid dairy foods and 20 g for solid dairy foods) and prediabetes risk and study variation as shown by the I^2^ and p-value for the Q test based on two-stage linear meta-analysis. The study-specific RRs and 95% CIs are visualized in squares. The area of the squares is proportional to the specific study weight of the overall meta-analysis. The diamond represents the pooled RR and 95% CI.


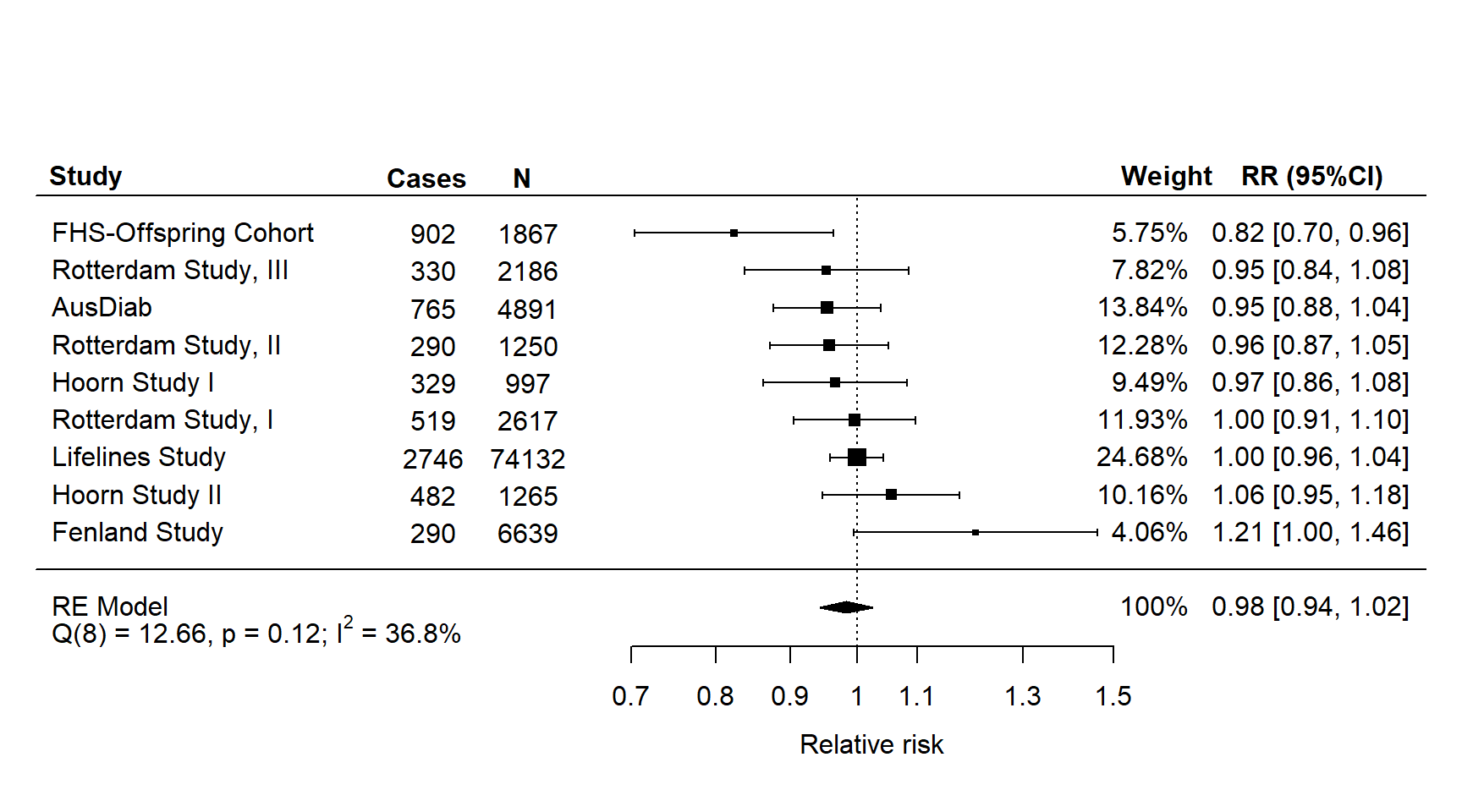


**Supplemental Figure 2.** Forest plot for the association between high-fat dairy intake (per increment of 1 serving/day, with serving sizes defined as 200 g for liquid dairy foods and 20 g for solid dairy foods) and prediabetes risk and study variation as shown by the I^2^ and p-value for the Q test based on two-stage linear meta-analysis. The study-specific RRs and 95% CIs are visualized in squares. The area of the squares is proportional to the specific study weight of the overall meta-analysis. The diamond represents the pooled RR and 95% CI.


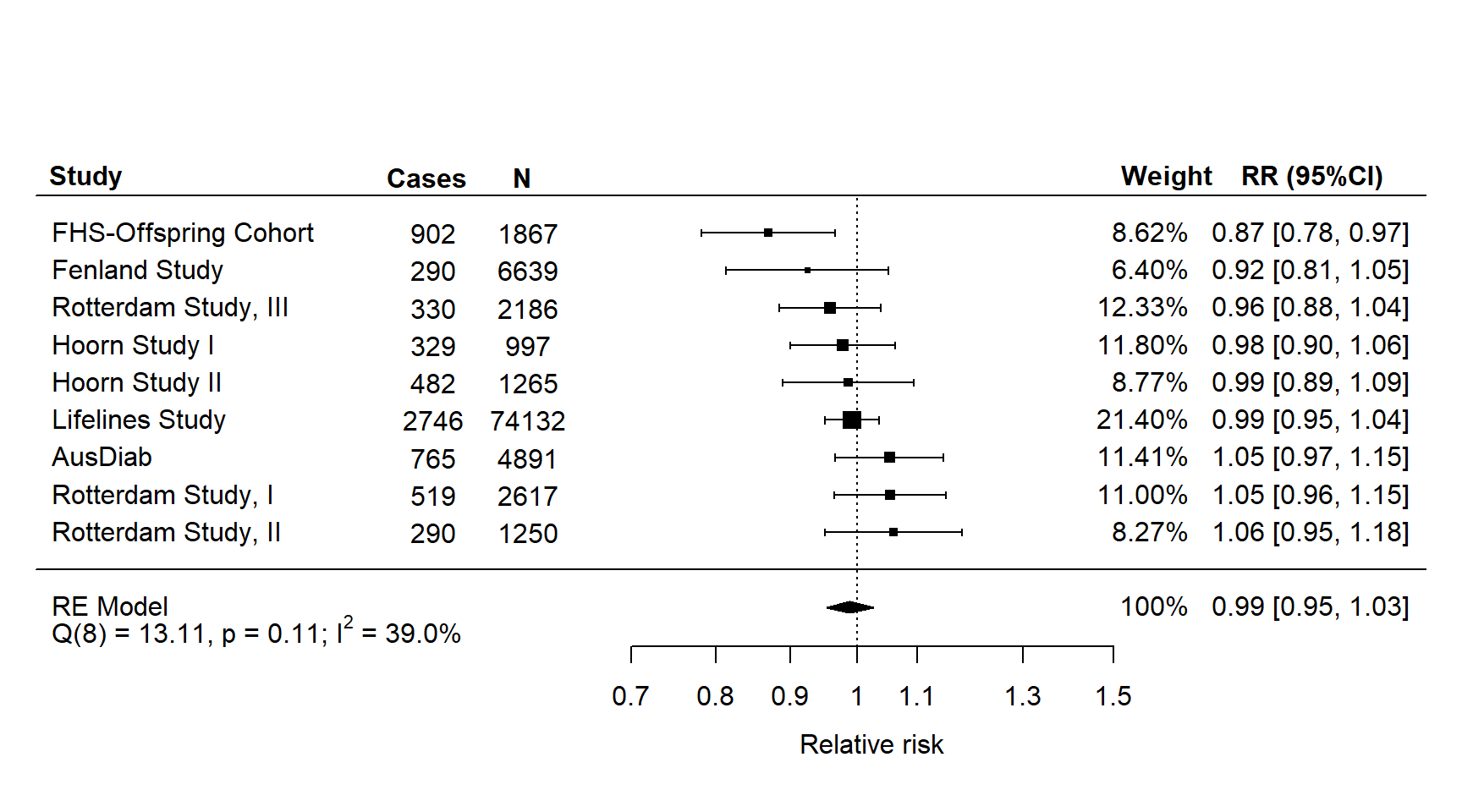


**Supplemental Figure 3.** Forest plot for the association between low-fat dairy intake (per increment of 1 serving/day, with serving sizes defined as 200 g for liquid dairy foods and 20 g for solid dairy foods) and prediabetes risk and study variation as shown by the I^2^ and p-value for the Q test based on two-stage linear meta-analysis. The study-specific RRs and 95% CIs are visualized in squares. The area of the squares is proportional to the specific study weight of the overall meta-analysis. The diamond represents the pooled RR and 95% CI.


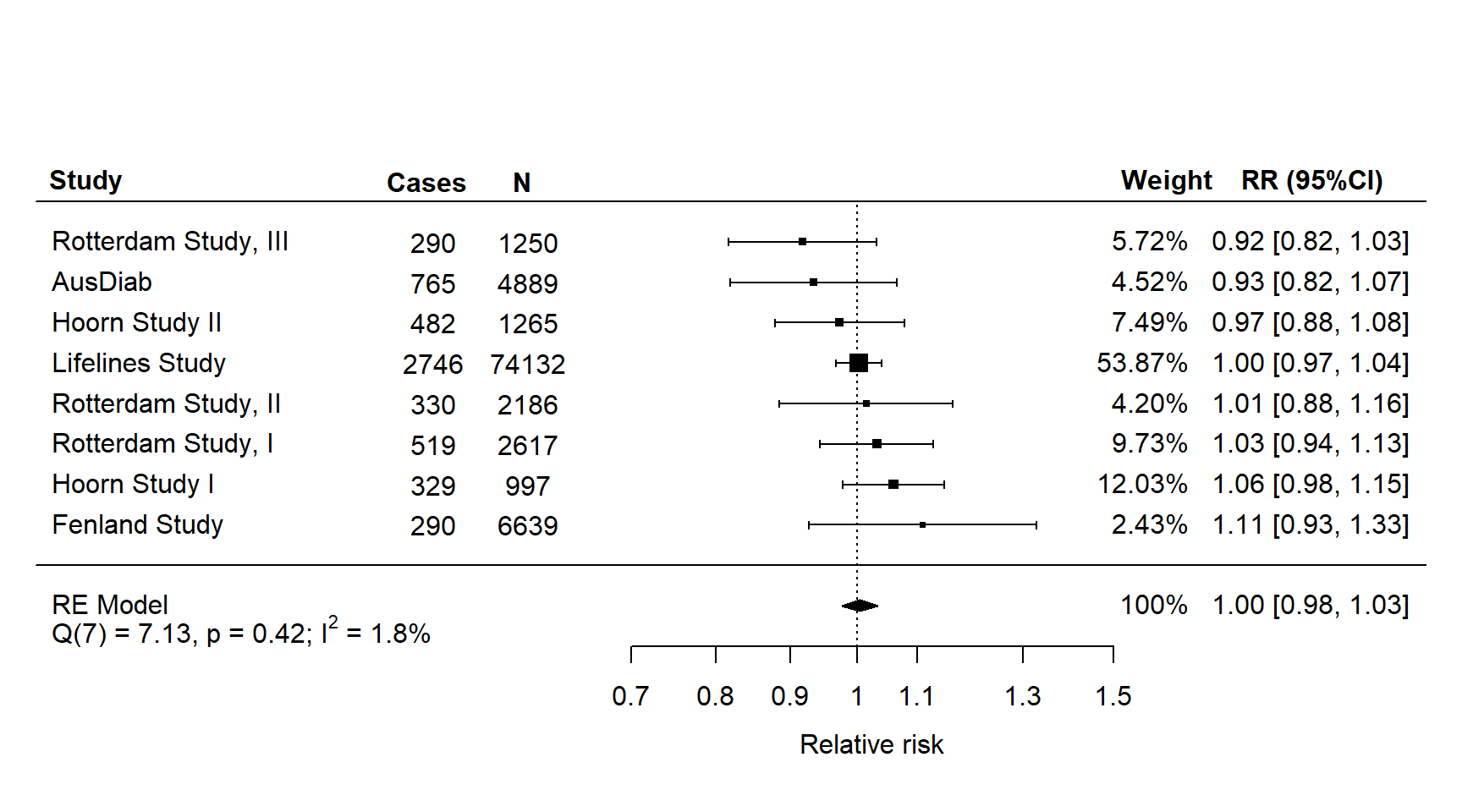


**Supplemental Figure 4.** Forest plot for the association between fermented dairy intake (per increment of 1 serving/day, with serving sizes defined as 200 g for liquid dairy foods and 20 g for solid dairy foods) and prediabetes risk and study variation as shown by the I^2^ and p-value for the Q test based on two-stage linear meta-analysis. The study-specific RRs and 95% CIs are visualized in squares. The area of the squares is proportional to the specific study weight of the overall meta-analysis. The diamond represents the pooled RR and 95% CI.


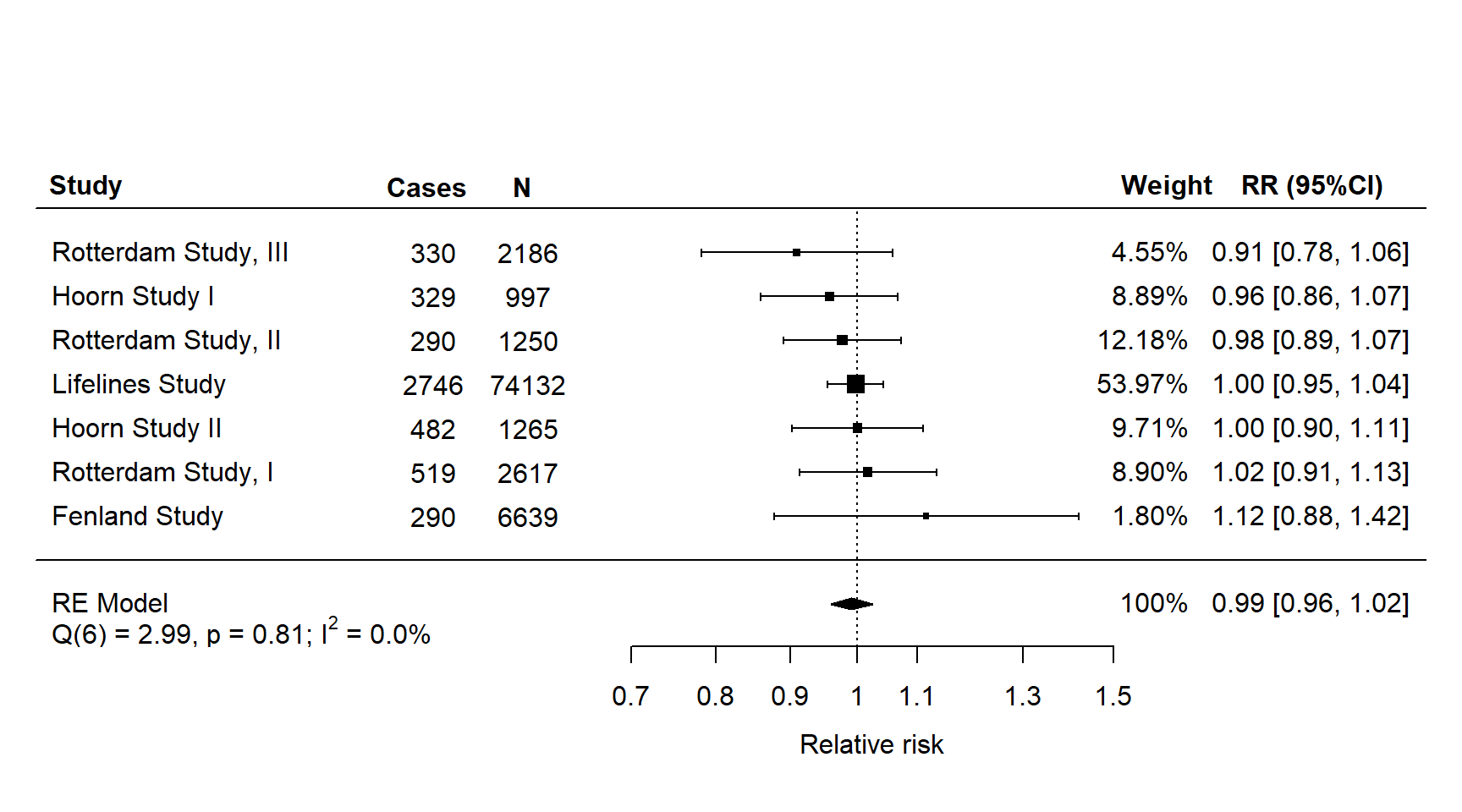
**Supplemental Figure 5.** Forest plot for the association between high-fat fermented dairy intake (per increment of 1 serving/day, with serving sizes defined as 200 g for liquid dairy foods and 20 g for solid dairy foods) and prediabetes risk and study variation as shown by the I^2^ and p-value for the Q test based on two-stage linear meta-analysis. The study-specific RRs and 95% CIs are visualized in squares. The area of the squares is proportional to the specific study weight of the overall meta-analysis. The diamond represents the pooled RR and 95% CI.


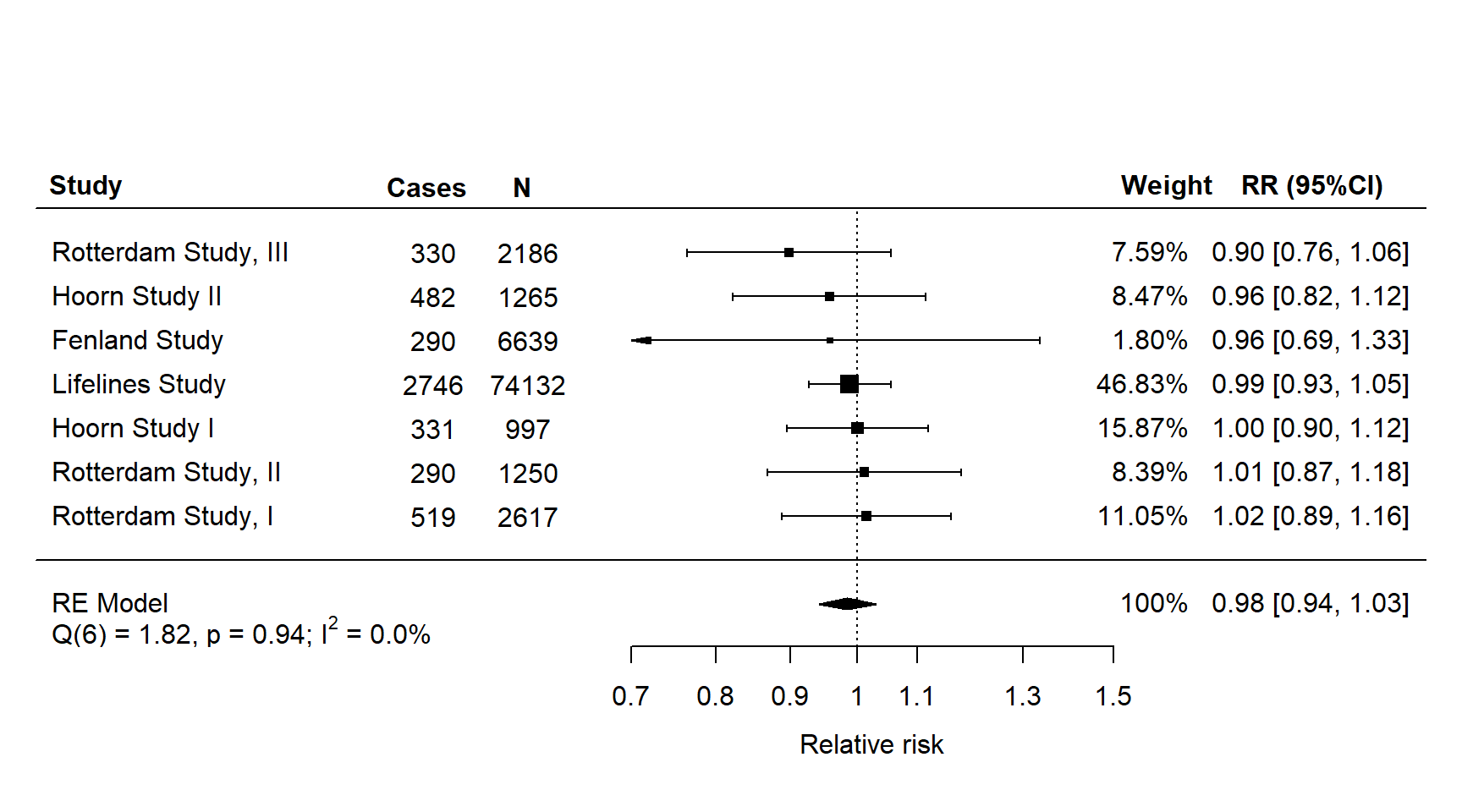


**Supplemental Figure 6.** Forest plot for the association between low-fat fermented dairy intake (per increment of 1 serving/day, with serving sizes defined as 200 g for liquid dairy foods and 20 g for solid dairy foods) and prediabetes risk and study variation as shown by the I^2^ and p-value for the Q test based on two-stage linear meta-analysis. The study-specific RRs and 95% CIs are visualized in squares. The area of the squares is proportional to the specific study weight of the overall meta-analysis. The diamond represents the pooled RR and 95% CI.


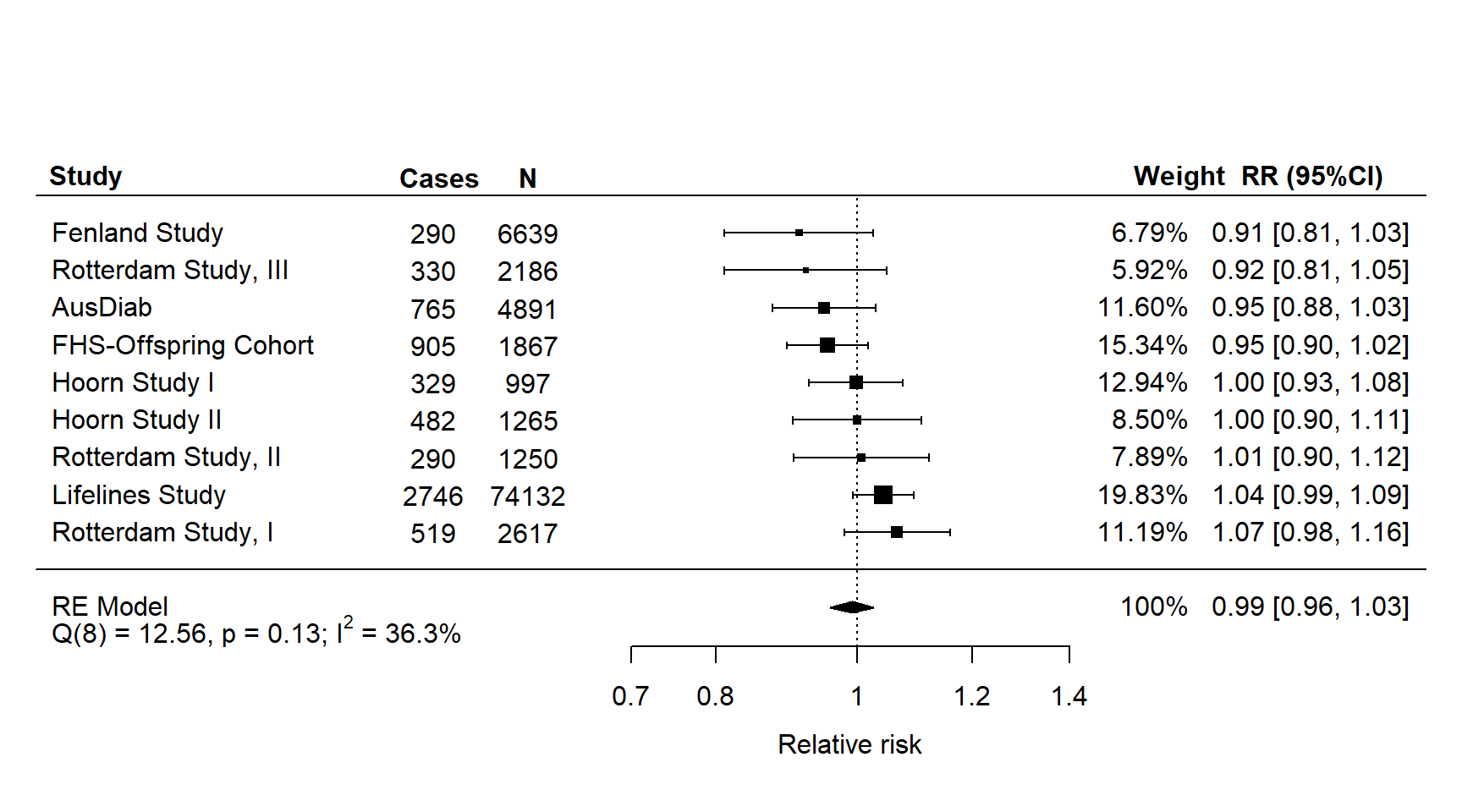


**Supplemental Figure 7.** Forest plot for the association between total milk intake (per increment of 1 serving/day, with a serving size defined as 150 g) and prediabetes risk and study variation as shown by the I^2^ and p-value for the Q test based on two-stage linear meta-analysis. The study-specific RRs and 95% CIs are visualized in squares. The area of the squares is proportional to the specific study weight of the overall meta-analysis. The diamond represents the pooled RR and 95% CI.


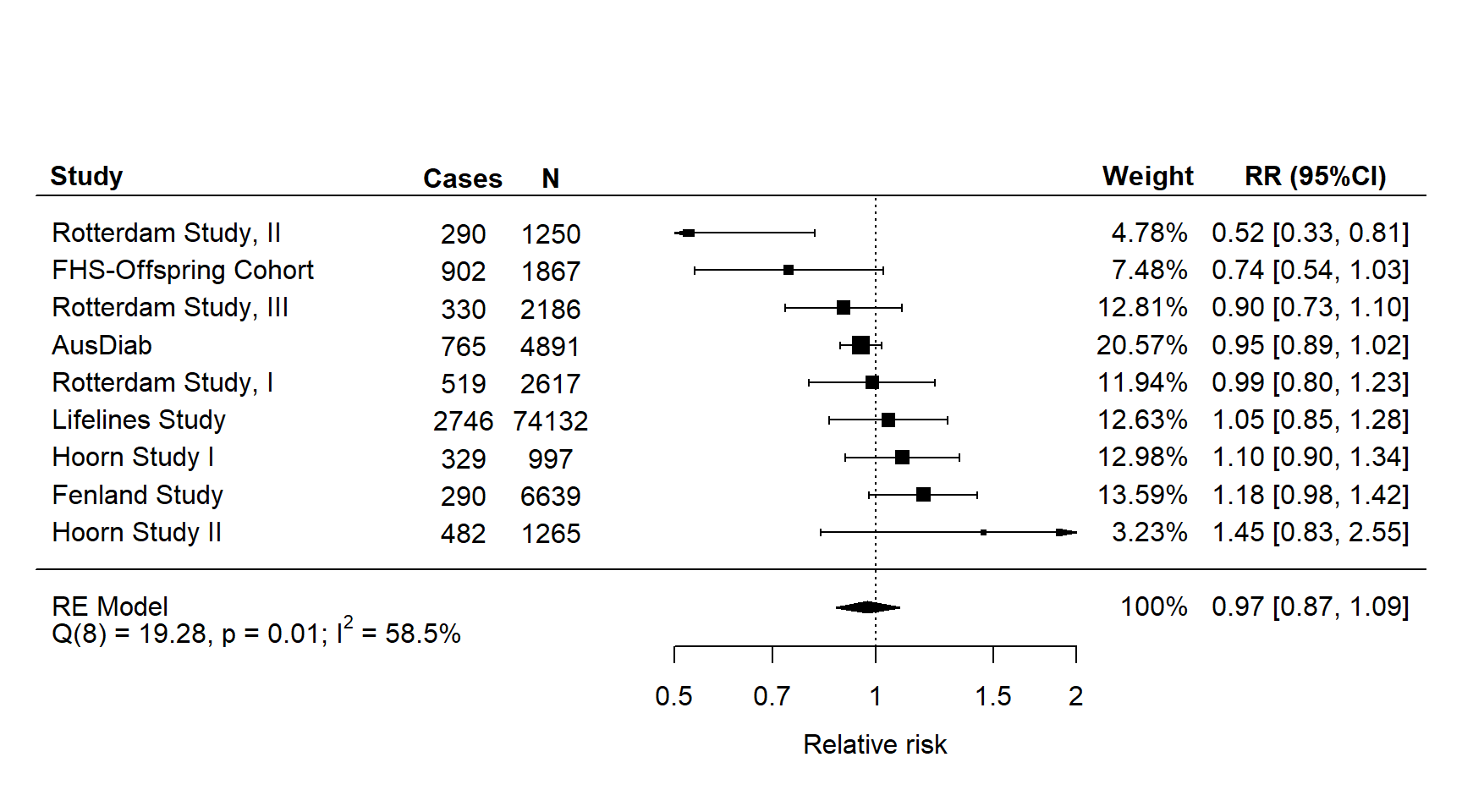


**Supplemental Figure 8.** Forest plot for the association between high-fat milk intake (per increment of 1 serving/day, with a serving size defined as 150 g) and prediabetes risk and study variation as shown by the I^2^ and p-value for the Q test based on two-stage linear meta-analysis. The study-specific RRs and 95% CIs are visualized in squares. The area of the squares is proportional to the specific study weight of the overall meta-analysis. The diamond represents the pooled RR and 95% CI.


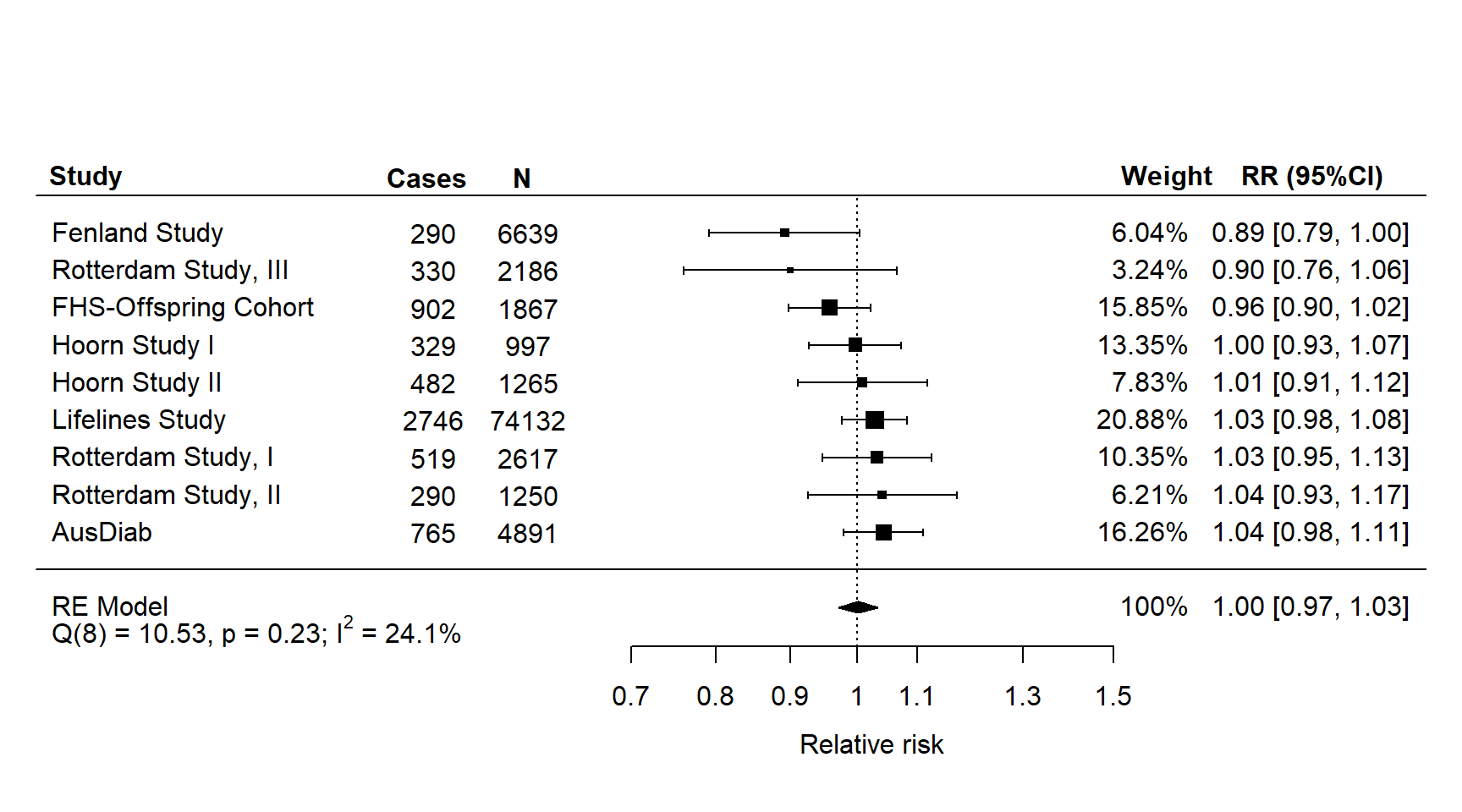


**Supplemental Figure 9.** Forest plot for the association between low-fat milk intake (per increment of 1 serving/day, with a serving size defined as 150 g) and prediabetes risk and study variation as shown by the I^2^ and p-value for the Q test based on two-stage linear meta-analysis. The study-specific RRs and 95% CIs are visualized in squares. The area of the squares is proportional to the specific study weight of the overall meta-analysis. The diamond represents the pooled RR and 95% CI.


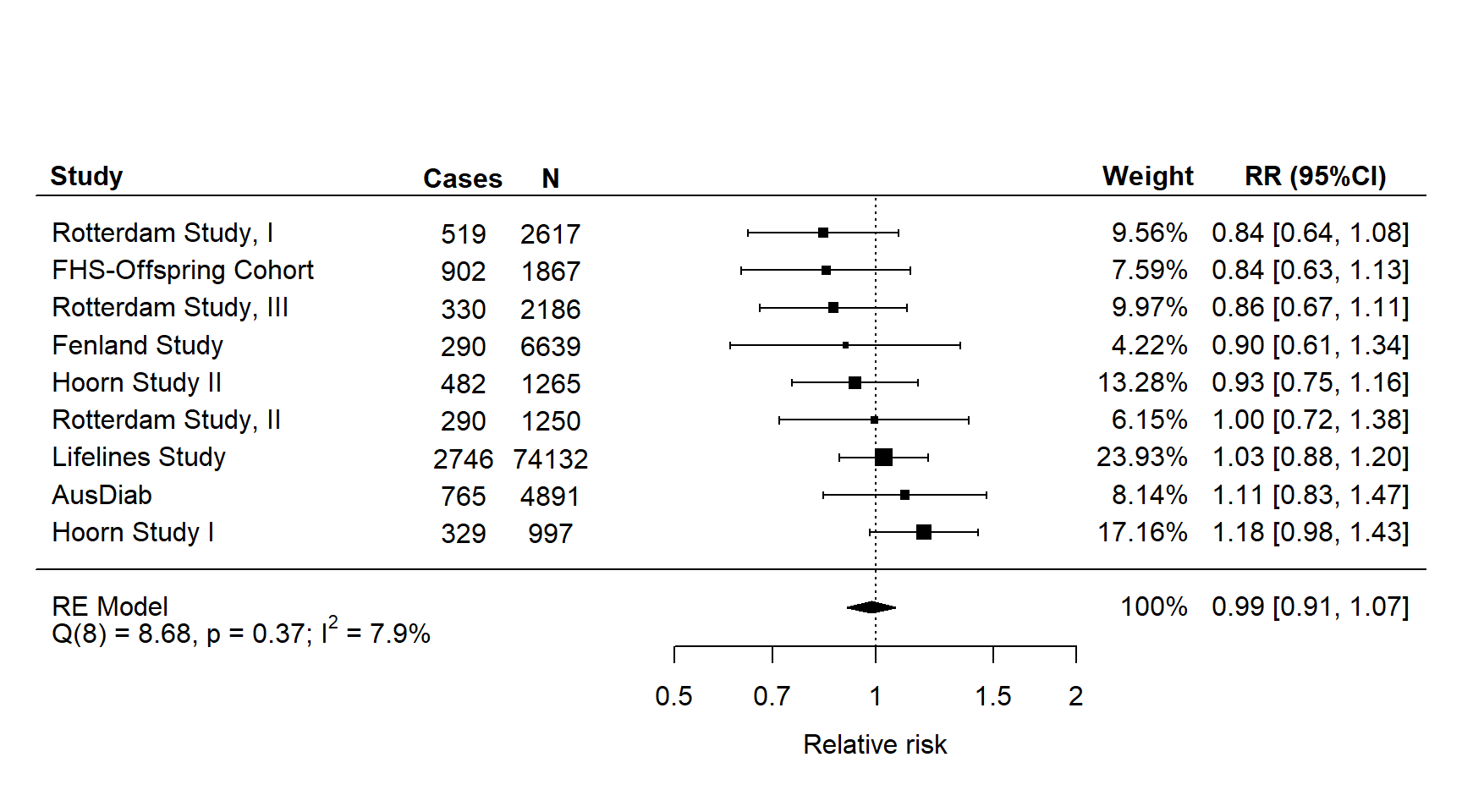


**Supplemental Figure 10.** Forest plot for the association between total yogurt intake (per increment of 1 serving/day, with a serving size defined as 150 g) and prediabetes risk and study variation as shown by the I^2^ and p-value for the Q test based on two-stage linear meta-analysis. The study-specific RRs and 95% CIs are visualized in squares. The area of the squares is proportional to the specific study weight of the overall meta-analysis. The diamond represents the pooled RR and 95% CI.


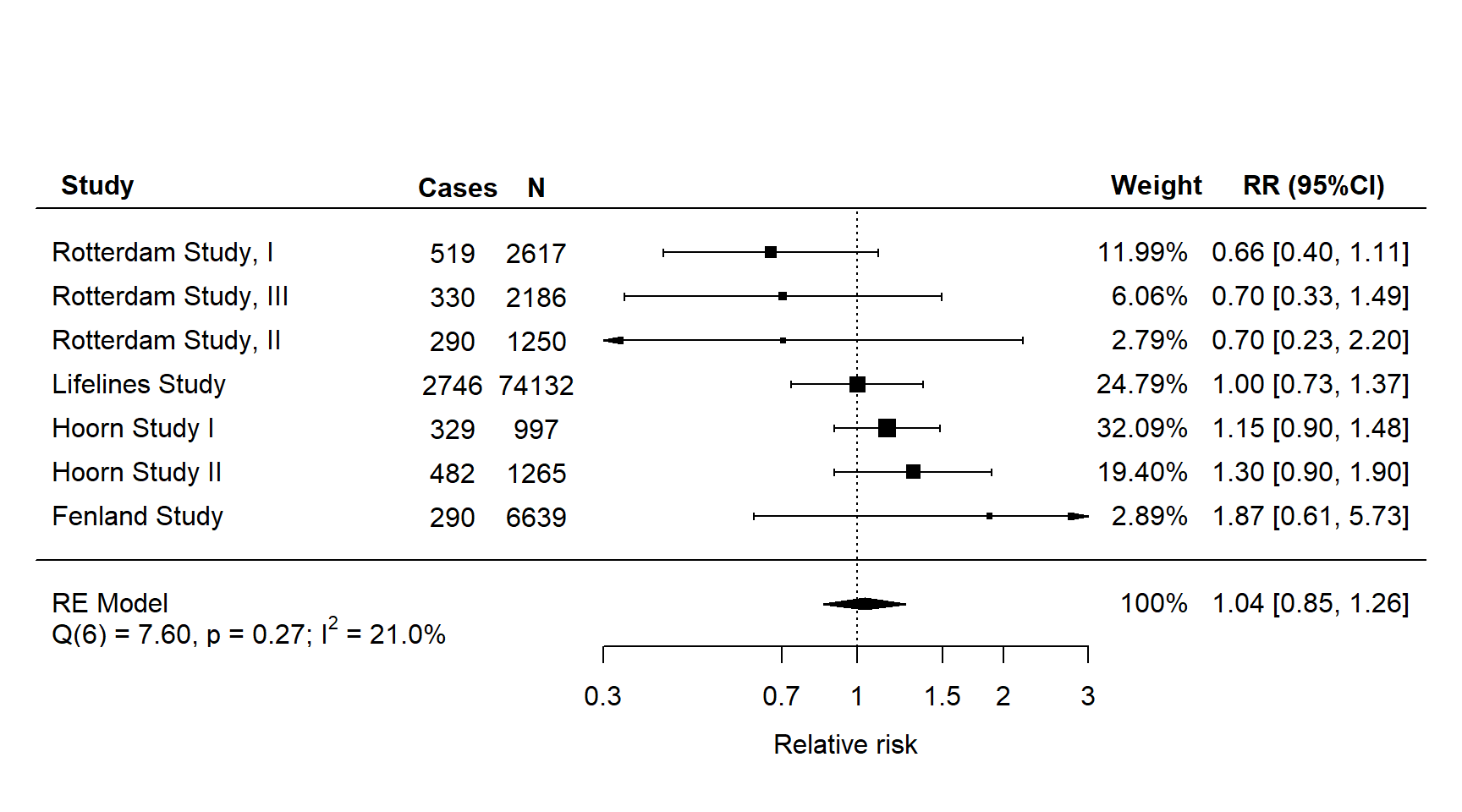


**Supplemental Figure 11.** Forest plot for the association between high-fat yogurt intake (per increment of 1 serving/day, with a serving size defined as 150 g) and prediabetes risk and study variation as shown by the I^2^ and p-value for the Q test based on two-stage linear meta-analysis. The study-specific RRs and 95% CIs are visualized in squares. The area of the squares is proportional to the specific study weight of the overall meta-analysis. The diamond represents the pooled RR and 95% CI.


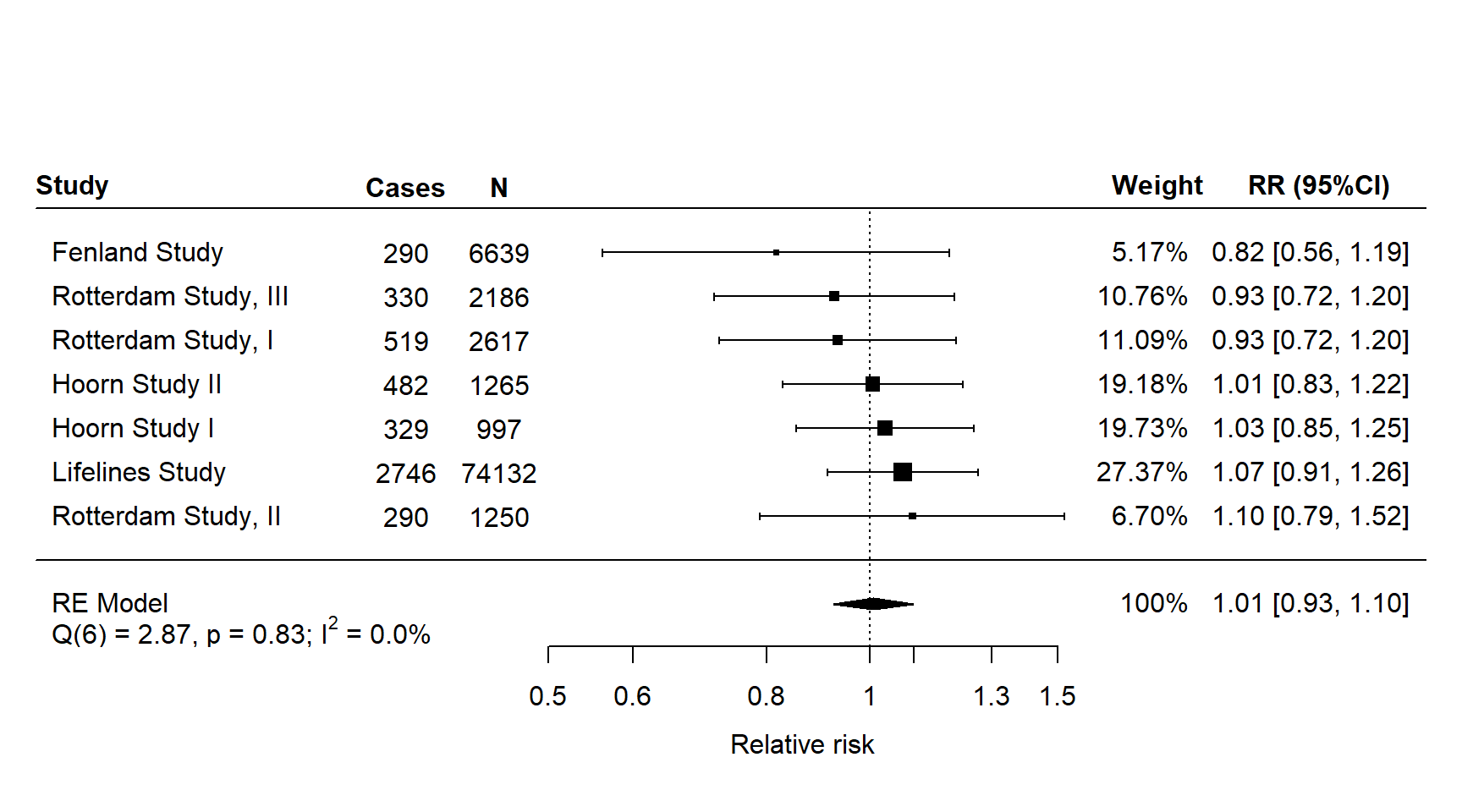


**Supplemental Figure 12.** Forest plot for the association between low-fat yogurt intake (per increment of 1 serving/day, with a serving size defined as 150 g) and prediabetes risk and study variation as shown by the I^2^ and p-value for the Q test based on two-stage linear meta-analysis. The study-specific RRs and 95% CIs are visualized in squares. The area of the squares is proportional to the specific study weight of the overall meta-analysis. The diamond represents the pooled RR and 95% CI.


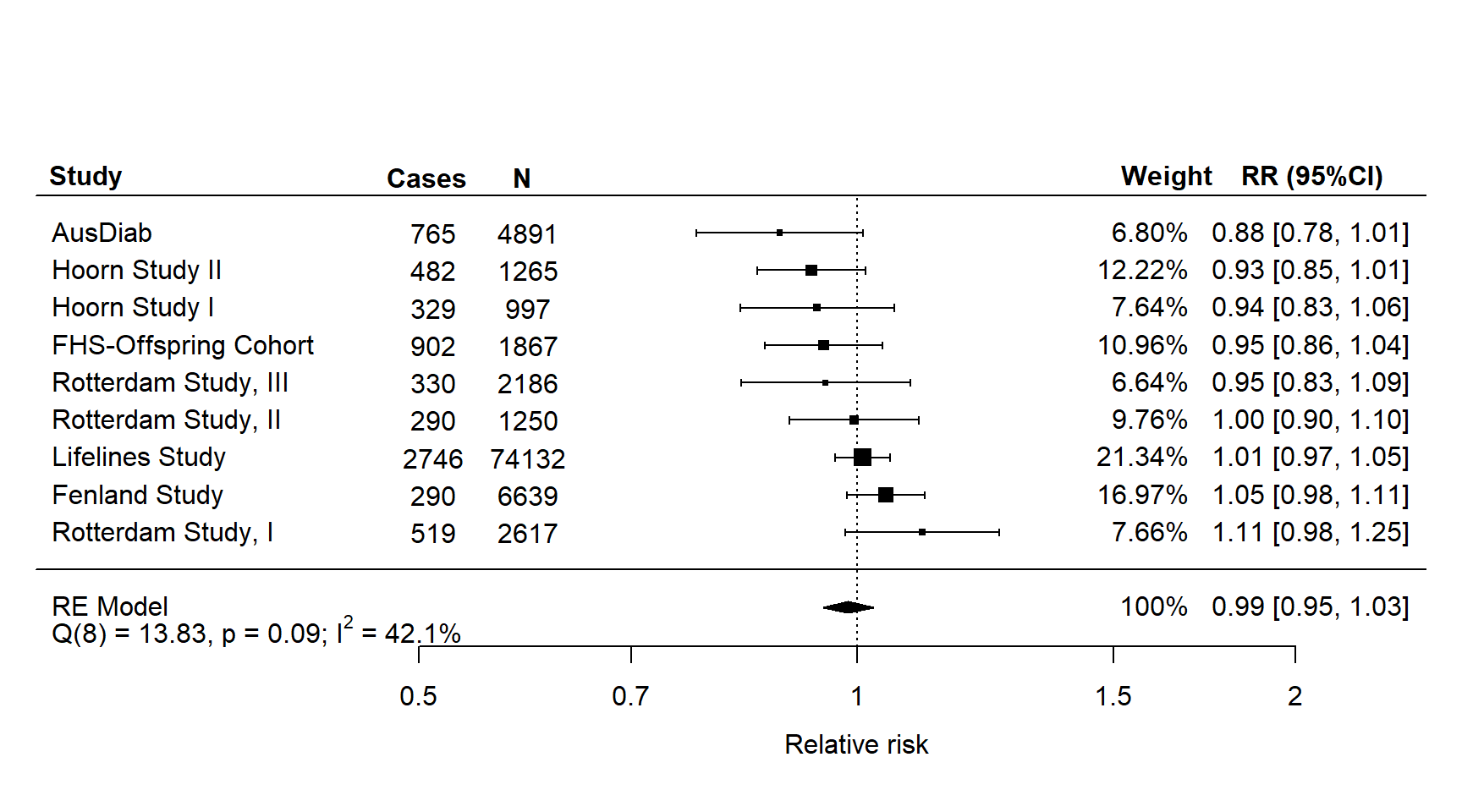


**Supplemental Figure 13.** Forest plot for the association between total cheese intake (per increment of 1 serving/day, with a serving size defined as 20 g) and prediabetes risk and study variation as shown by the I^2^ and p-value for the Q test based on two-stage linear meta-analysis. The study-specific RRs and 95% CIs are visualized in squares. The area of the squares is proportional to the specific study weight of the overall meta-analysis. The diamond represents the pooled RR and 95% CI.


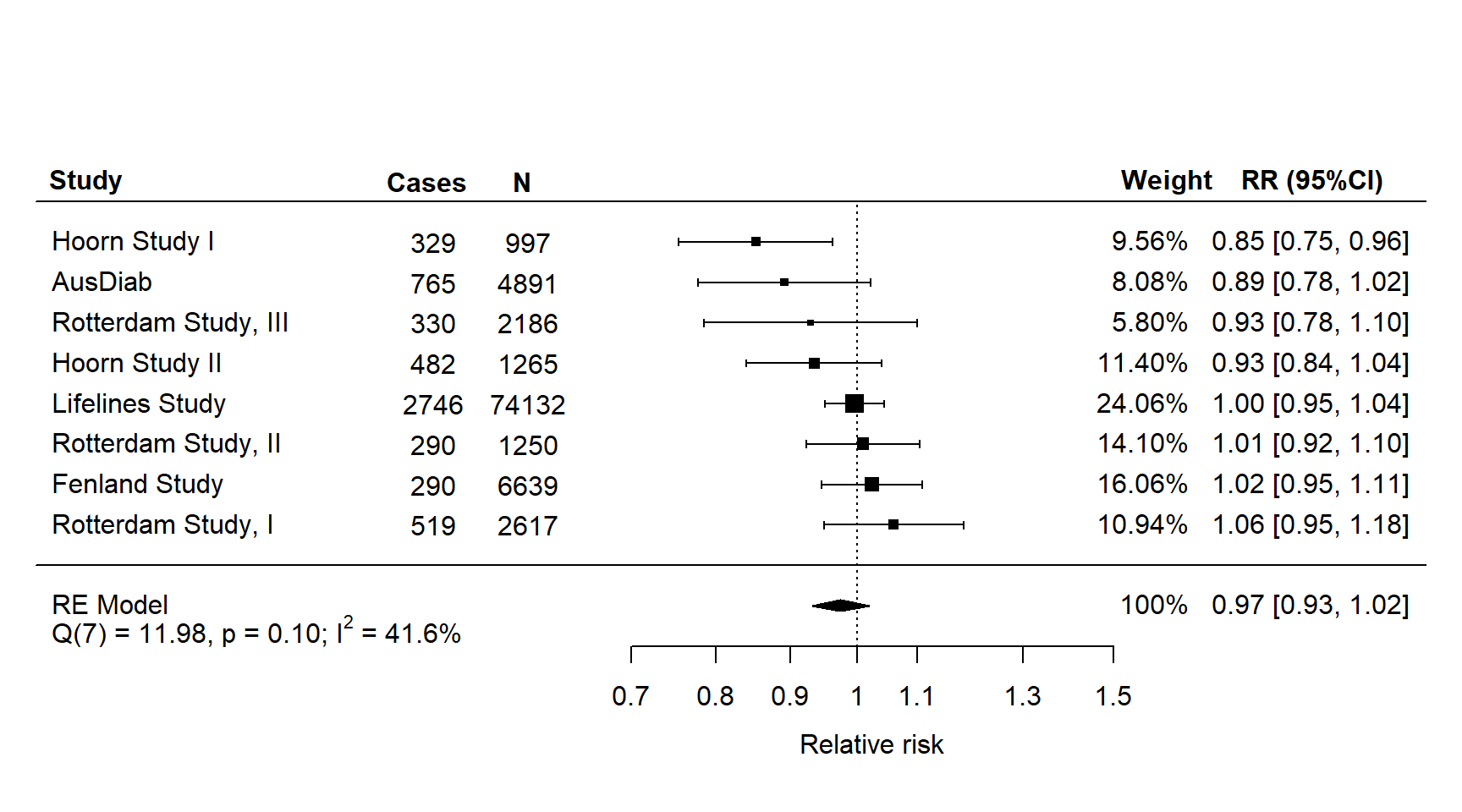


**Supplemental Figure 14.** Forest plot for the association between high-fat cheese intake (per increment of 1 serving/day, with a serving size defined as 20 g) and prediabetes risk and study variation as shown by the I^2^ and p-value for the Q test based on two-stage linear meta-analysis. The study-specific RRs and 95% CIs are visualized in squares. The area of the squares is proportional to the specific study weight of the overall meta-analysis. The diamond represents the pooled RR and 95% CI.


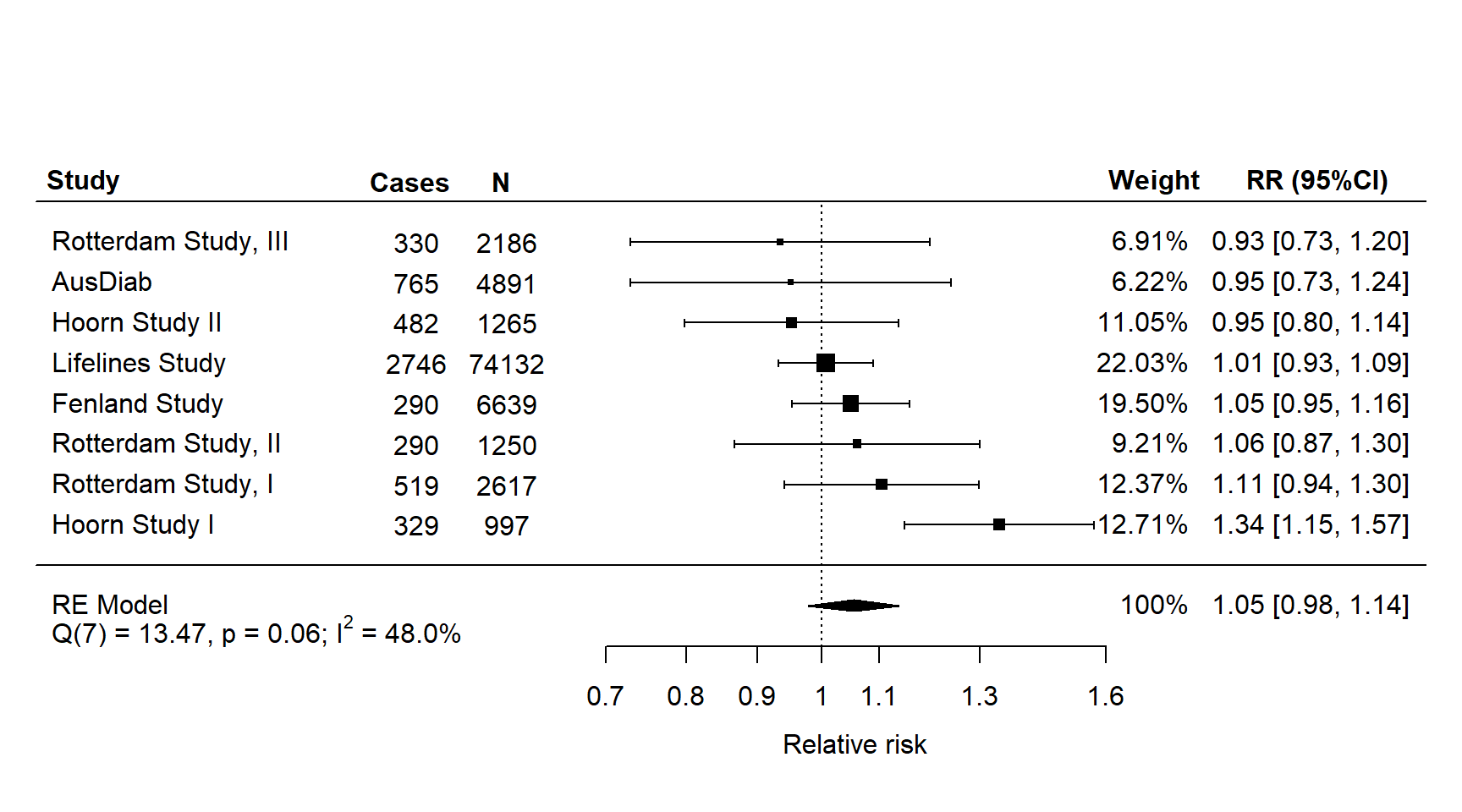


**Supplemental Figure 15.** Forest plot for the association between low-fat cheese intake (per increment of 1 serving/day, with a serving size defined as 20 g) and prediabetes risk and study variation as shown by the I^2^ and p-value for the Q test based on two-stage linear meta-analysis. The study-specific RRs and 95% CIs are visualized in squares. The area of the squares is proportional to the specific study weight of the overall meta-analysis. The diamond represents the pooled RR and 95% CI.


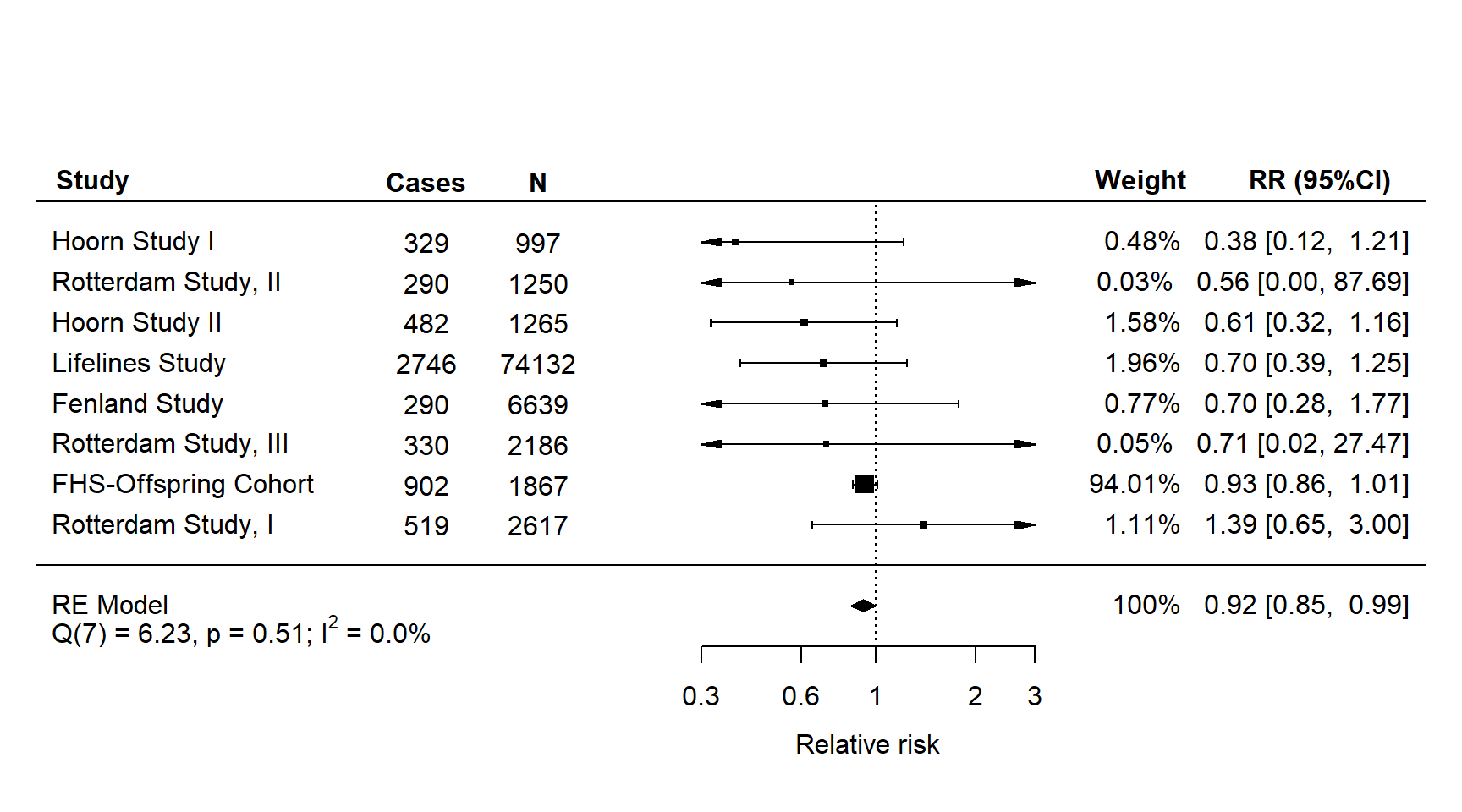


**Supplemental Figure 16.** Forest plot for the association between cream intake (per increment of 1 serving/day, with a serving size defined as 15 g) and prediabetes risk and study variation as shown by the I^2^ and p-value for the Q test based on two-stage linear meta-analysis. The study-specific RRs and 95% CIs are visualized in squares. The area of the squares is proportional to the specific study weight of the overall meta-analysis. The diamond represents the pooled RR and 95% CI.


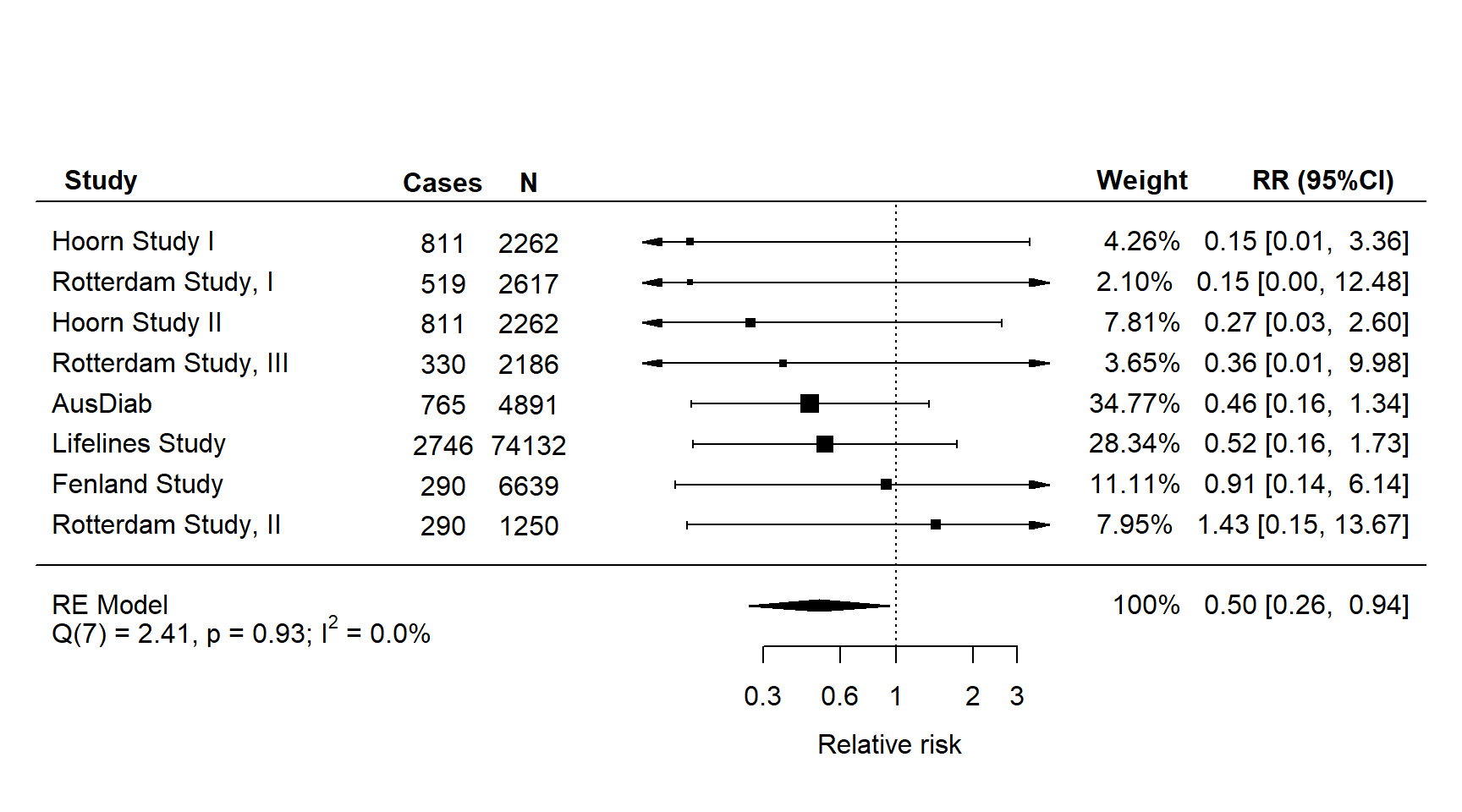


**Supplemental Figure 17.** Forest plot for the association between ice cream intake (per increment of 1 serving/day, with a serving size defined as 150 g) and prediabetes risk and study variation as shown by the I^2^ and p-value for the Q test based on two-stage linear meta-analysis. The study-specific RRs and 95% CIs are visualized in squares. The area of the squares is proportional to the specific study weight of the overall meta-analysis. The diamond represents the pooled RR and 95% CI.

**
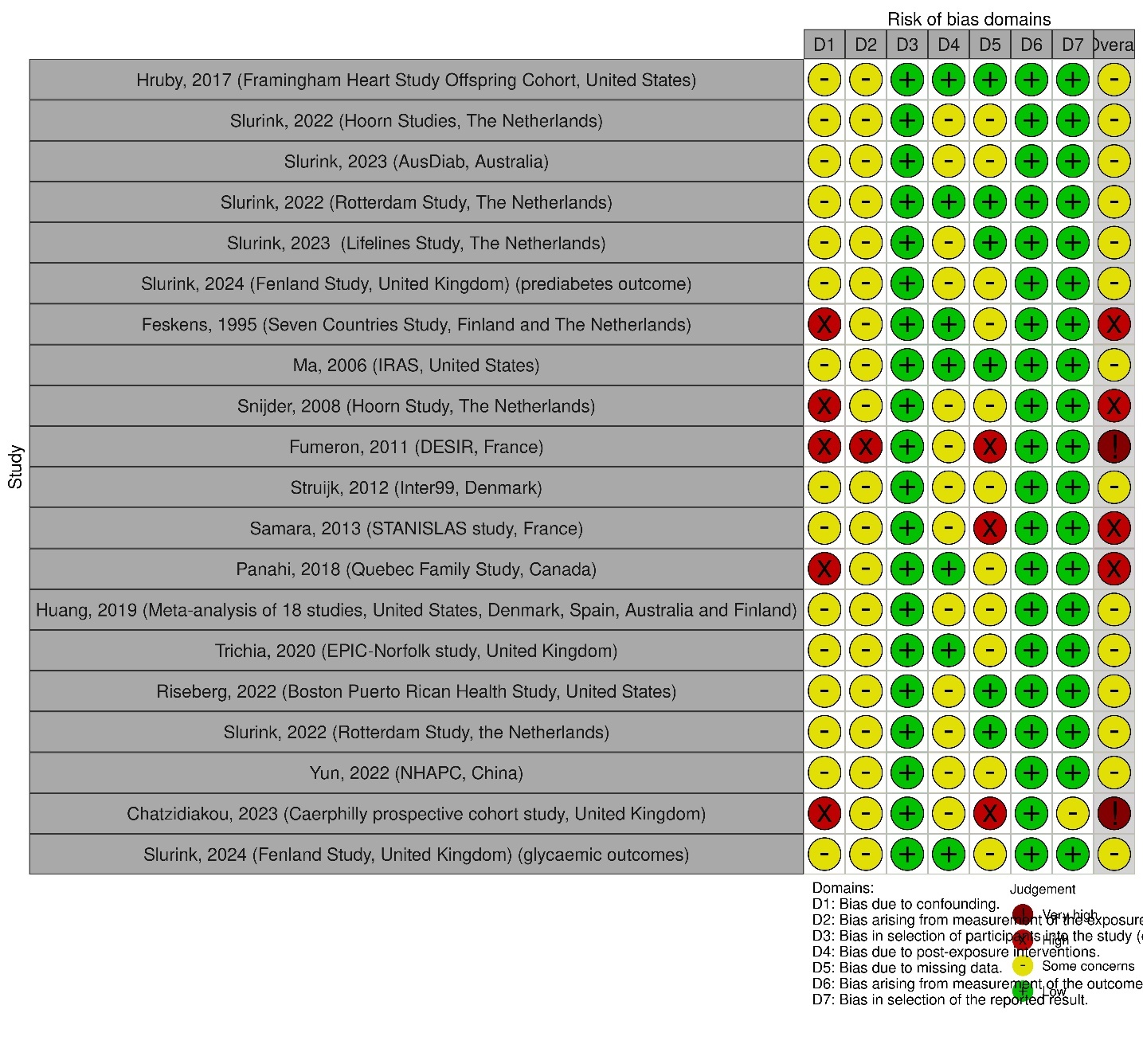
**

**
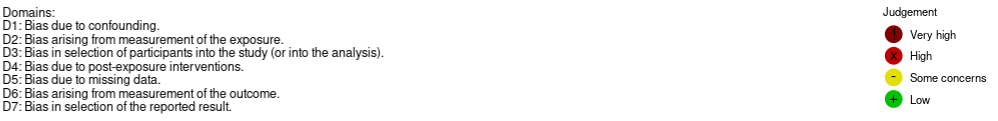

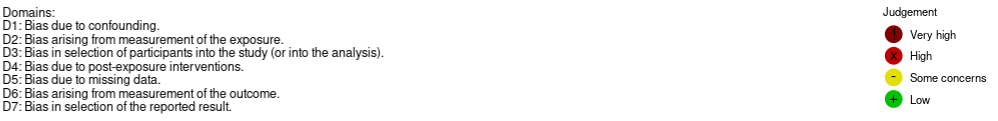
**

**Supplemental figure 18:** Risk of bias of each study for each domain and overall assessment based on the assessed with the ROBINS-E (Risk Of Bias In Non-randomized Studies - of Exposure) tool.

**a**
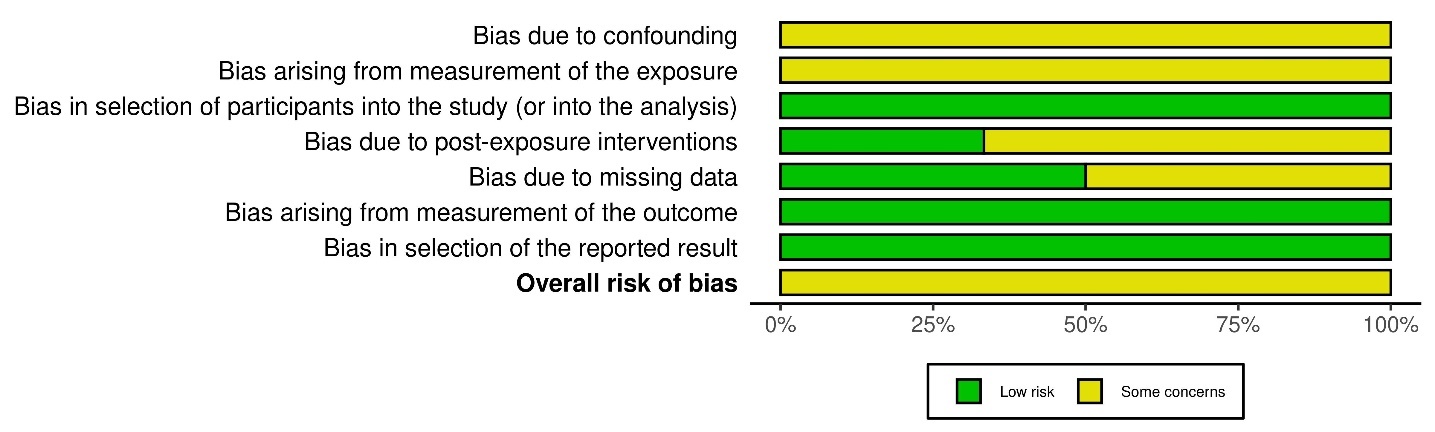


**b**
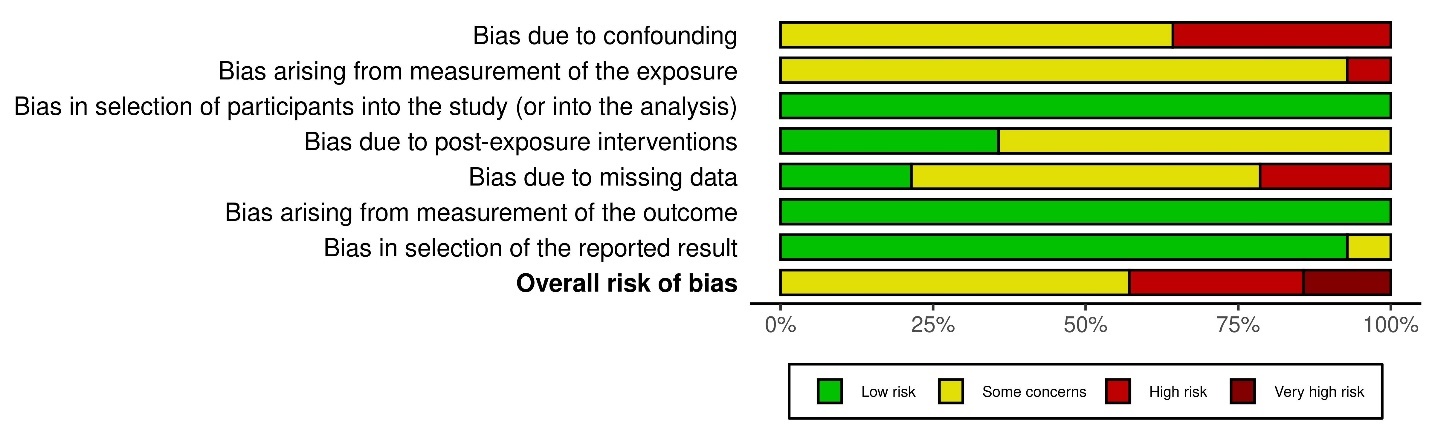


**Supplemental figure 19.** Overall risk of bias of the included studies assessed with the ROBINS-E (Risk Of Bias In Non-randomized Studies - of Exposure) tool. **a**) Including 6 studies on the relation between intake of different dairy types and prediabetes risk. **b**) Including 14 studies assessing the relation between intake of different dairy types and continuous glycemic outcomes.


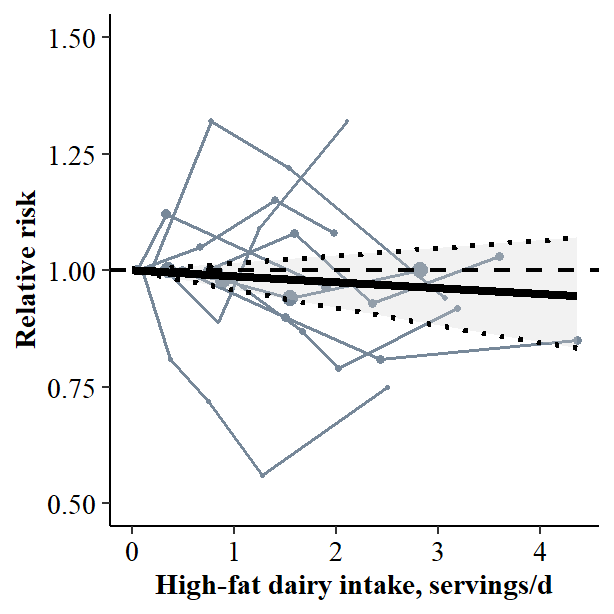

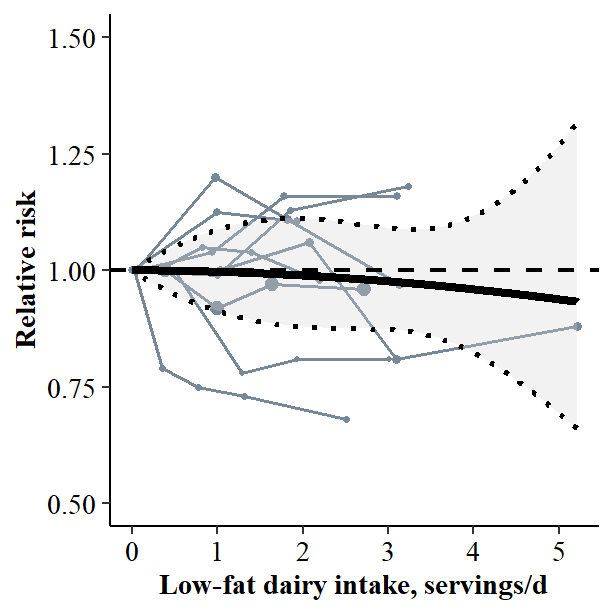


**Supplemental figure 20.** Spaghetti plot based on dose-response meta-analysis including 6 studies and 9 cohorts (6,653 cases among 95,844 participants) for the associations between **high-fat dairy** (RR per serving/day 0.99, 95%CI 0.96-1.02, I^2^ = 37%)(left), and **low-fat dairy** (lowest RR at 5.2 servings/day: 0.93, 95%CI 0.66-1.32, I^2^ = 28%)(right) intake and prediabetes risk. The solid black line represents the pooled RR at each quantity of intake. The light grey coloured area between the dotted black lines indicates the 95% confidence interval. The dashed grey line at RR = 1.00 represents the reference line. Each solid grey line represents a cohort with circles placed at the cohort-specific RRs at the corresponding intake level. The area of the circle is proportional to the study-specific weight. The associations were adjusted for age, sex, energy intake, educational level, smoking behaviour, physical activity, alcohol intake, family history of diabetes, intake of food groups, waist circumference or BMI, hypertension, and dyslipidaemia. Serving sizes were 200 g for liquid dairy foods and 20 g for solid dairy foods.


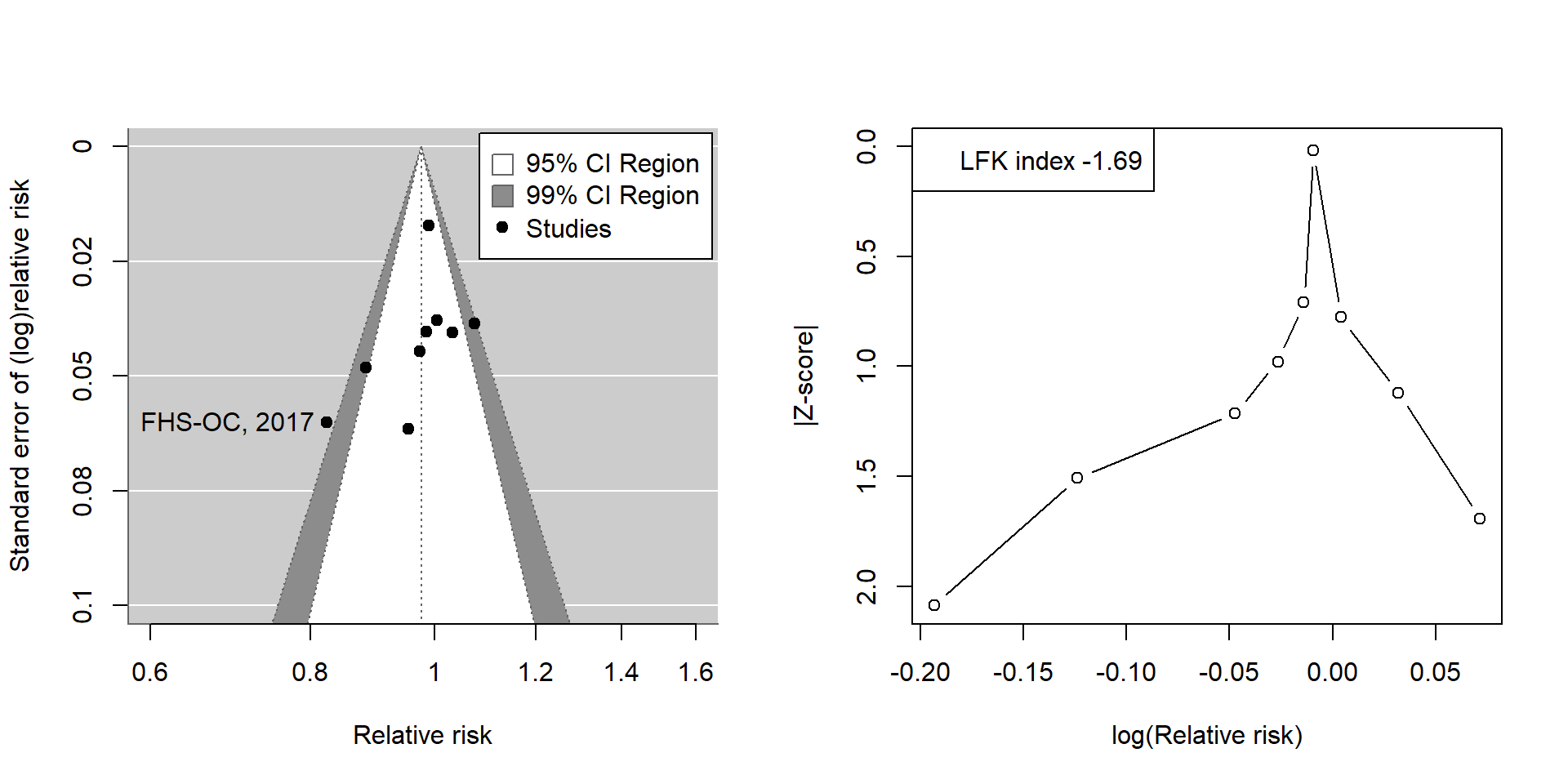


**Supplemental figure 21.** Contour-enhanced funnel plot (left) and Doi plot (right) for studies of the association between total dairy intake and prediabetes risk. Each dot indicates a study population. Egger’s test, P=0.11. LFK index = 1.69 indicating minor asymmetry.


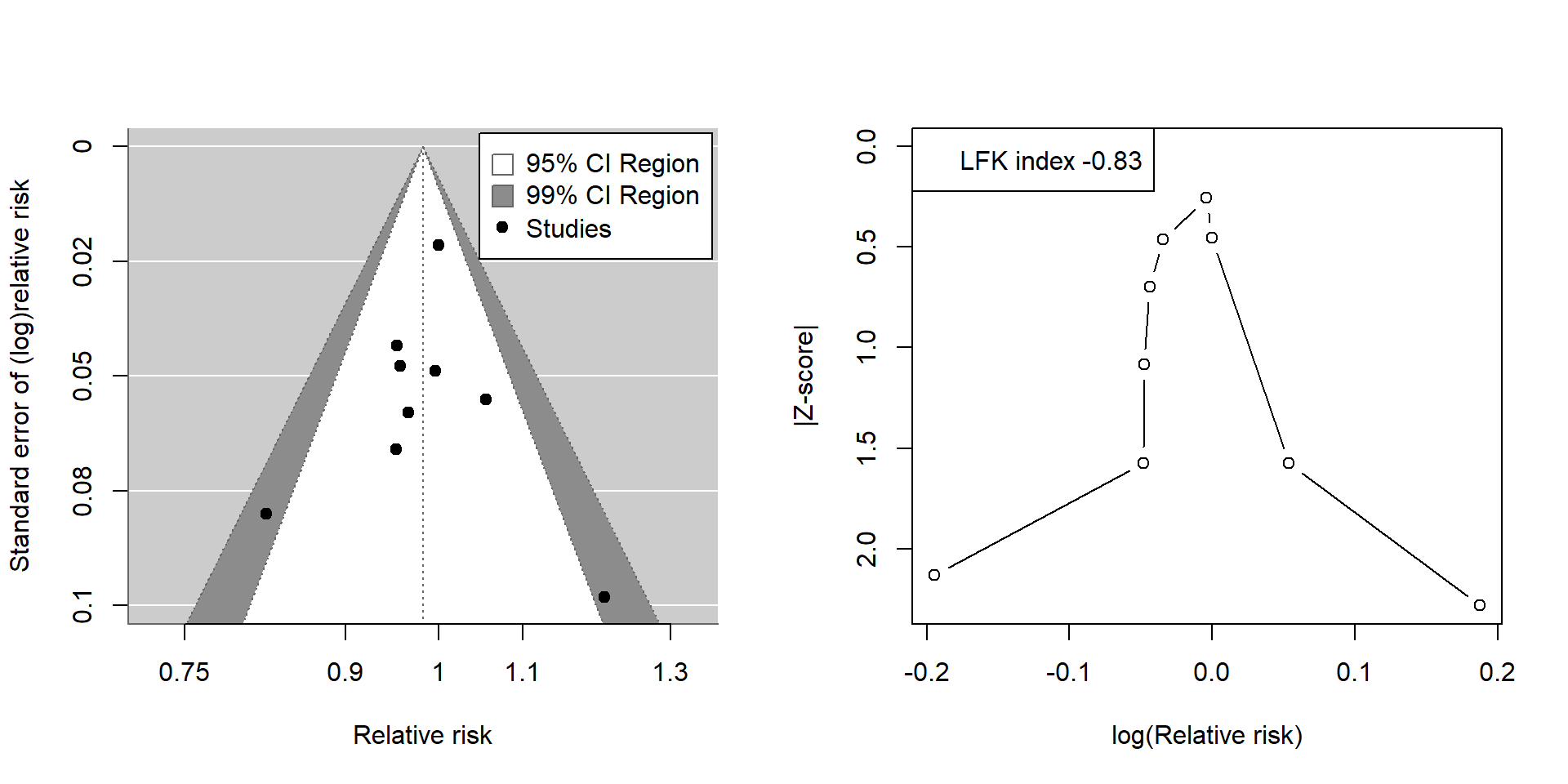


**Supplemental figure 22.** Contour-enhanced funnel plot (left) and Doi plot (right) for studies of the association between high-fat dairy intake and prediabetes risk. Each dot indicates a study population. Egger’s test, P=0.94. LFK index = -0.83 indicating no asymmetry.


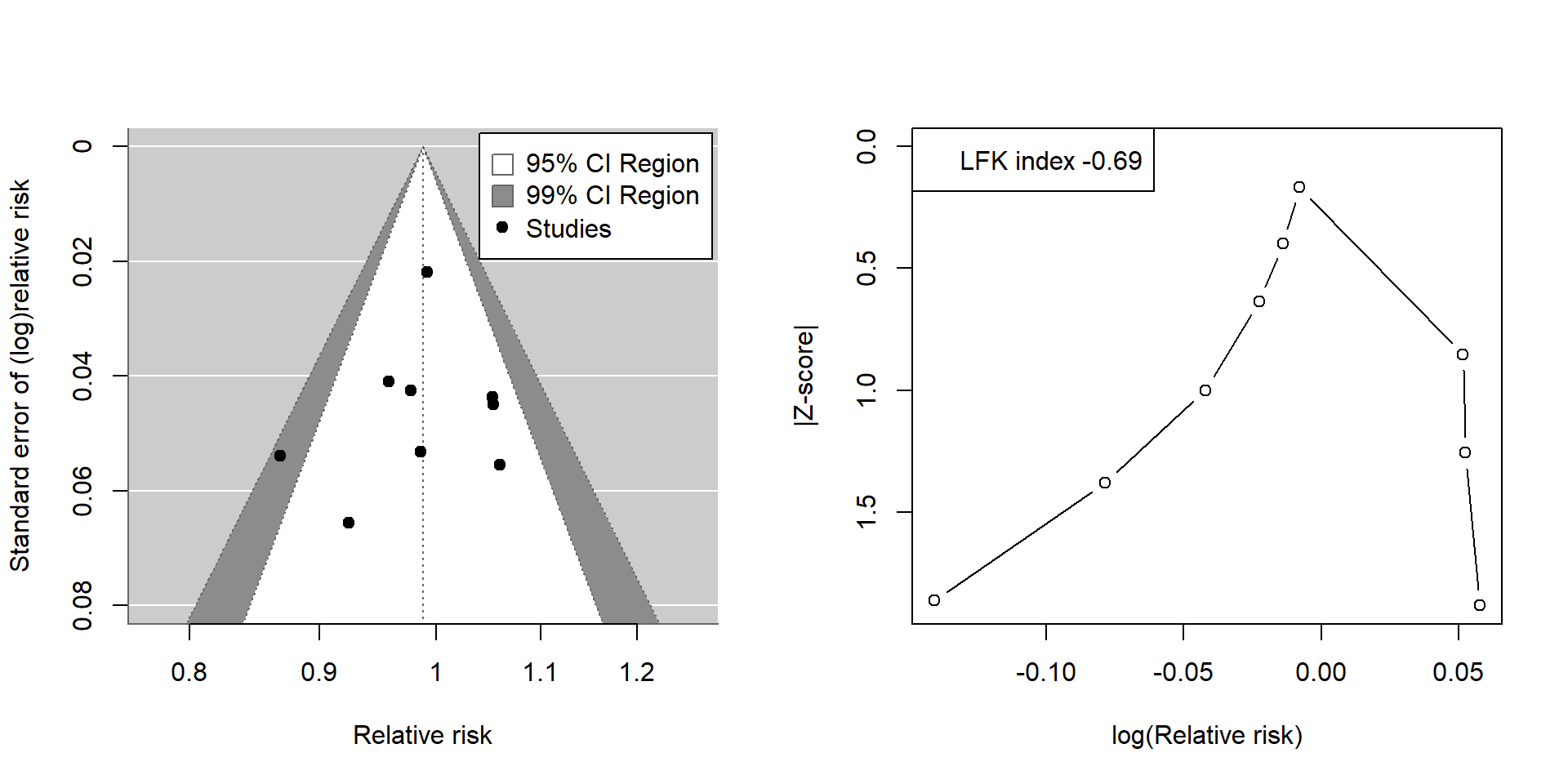


**Supplemental figure 23.** Contour-enhanced funnel plot (left) and Doi plot (right) for studies of the association between low-fat dairy intake and prediabetes risk. Each dot indicates a study population. Egger’s test, P=0.57. LFK index = -0.69 indicating no asymmetry.


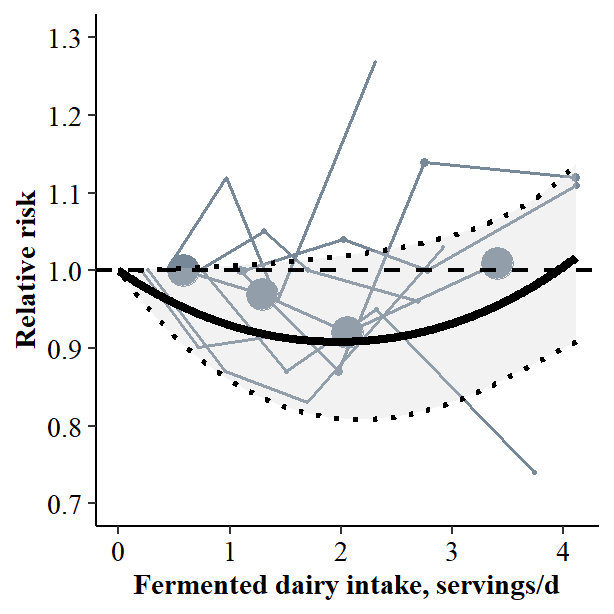

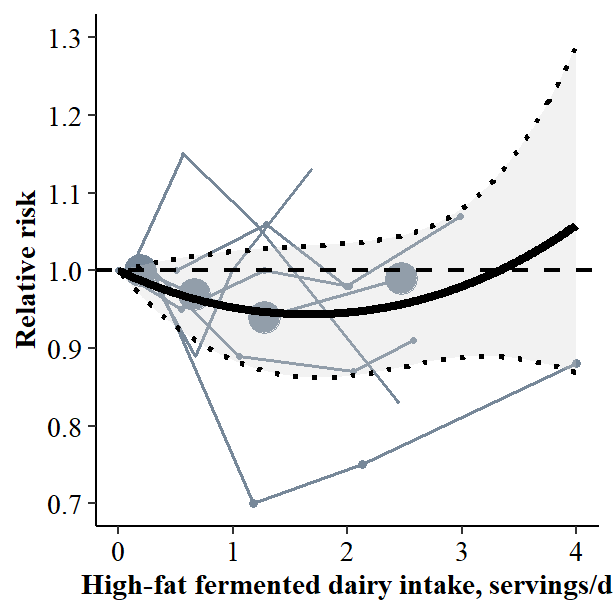

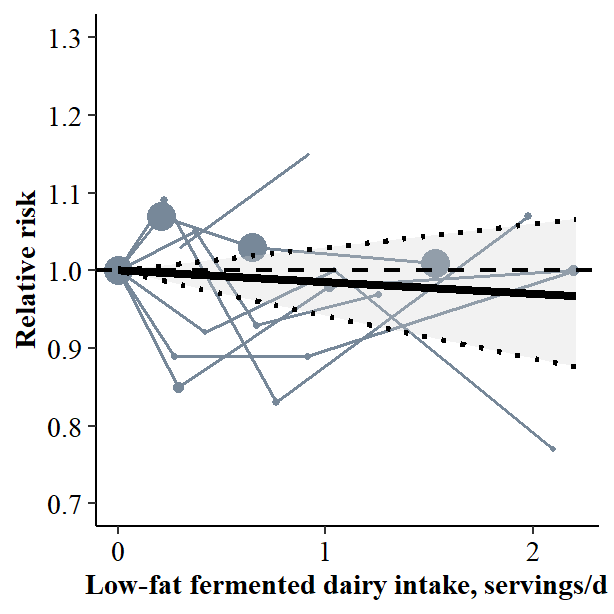


**Supplemental figure 24.** Spaghetti plot based on dose-response meta-analysis for the associations between **fermented dairy** (lowest RR at 2 servings/day: 0.91, 95%CI 0.81-1.02, I^2^ = 0%, including 5 studies and 8 cohorts, 5,751 cases among 93,975 participants)(left), **high-fat fermented dairy** (lowest RR at 1.7 servings/day: 0.94, 95%CI 0.81-1.02, I^2^ = 0%, including 4 studies and 7 cohorts, 4,986 cases among 89,089 participants)(middle), and **low-fat fermented dairy** (RR serving/day: 0.98, 95%CI 0.94-1.03, I^2^ = 28%, including 4 studies and 7 cohorts, 4,986 cases among 89,089 participants)(right) intake and prediabetes risk. The solid black line represents the pooled RR at each quantity of intake. The light grey colored area between the dotted black lines indicates the 95% confidence interval. The dashed grey line at RR = 1.00 represents the reference line. Each solid grey line represents a cohort with circles placed at the cohort-specific RRs at the corresponding intake level. The area of the circle is proportional to the study-specific weight. The associations were adjusted for age, sex, energy intake, educational level, smoking behavior, physical activity, alcohol intake, family history of diabetes, intake of food groups, waist circumference or BMI, hypertension, and dyslipidemia. Serving sizes were 200 g for liquid dairy foods and 20 g for solid dairy foods.


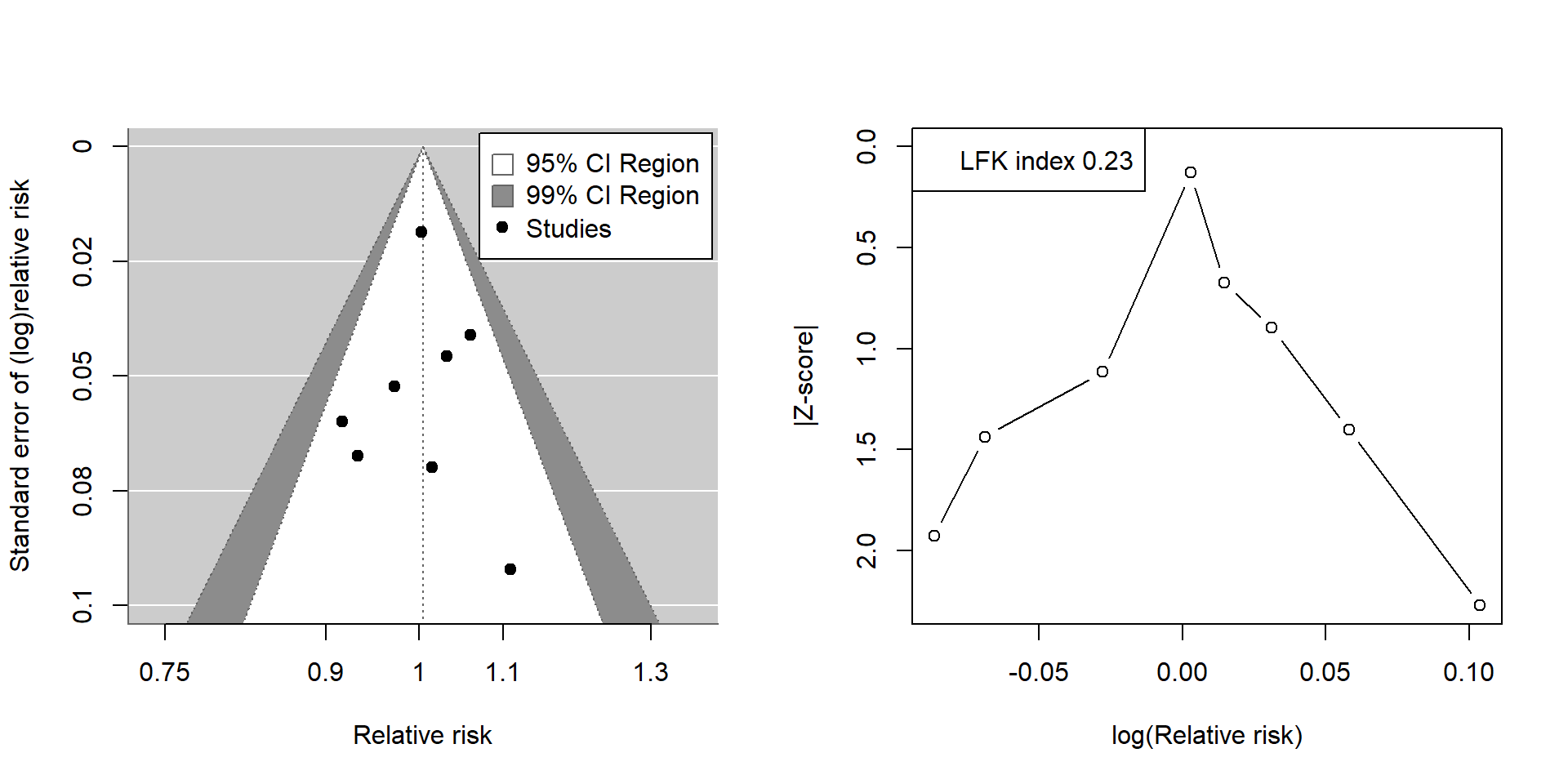


**Supplemental figure 25.** Contour-enhanced funnel plot (left) and Doi plot (right) for studies of the association between fermented dairy intake and prediabetes risk. Each dot indicates a study population. Egger’s test, P=0.86. LFK index = 0.23 indicating no asymmetry.


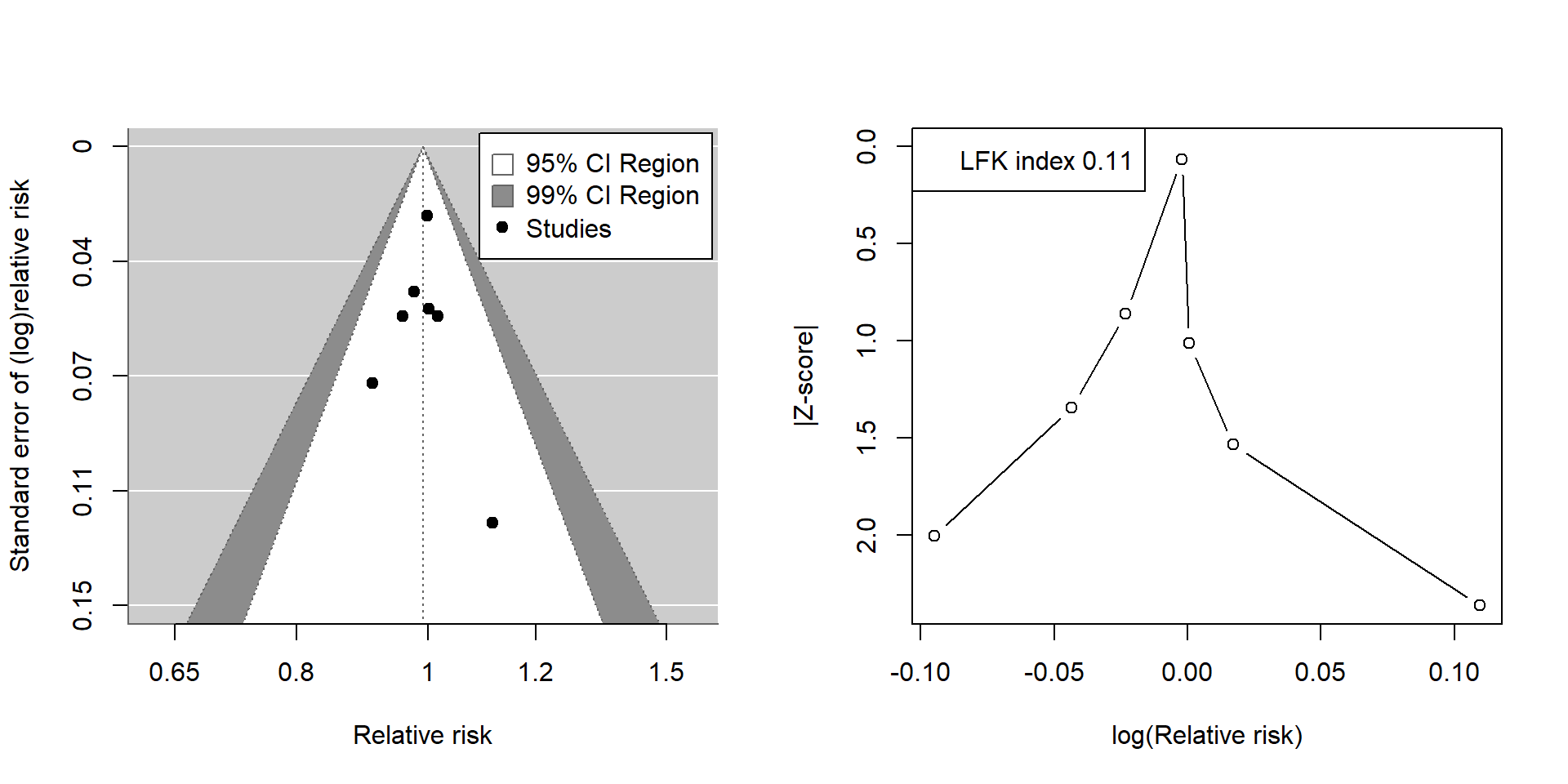


**Supplemental figure 26.** Contour-enhanced funnel plot (left) and Doi plot (right) for studies of the association between high-fat fermented dairy intake and prediabetes risk. Each dot indicates a study population. Egger’s test, P=0.89. LFK index = 0.11 no asymmetry.


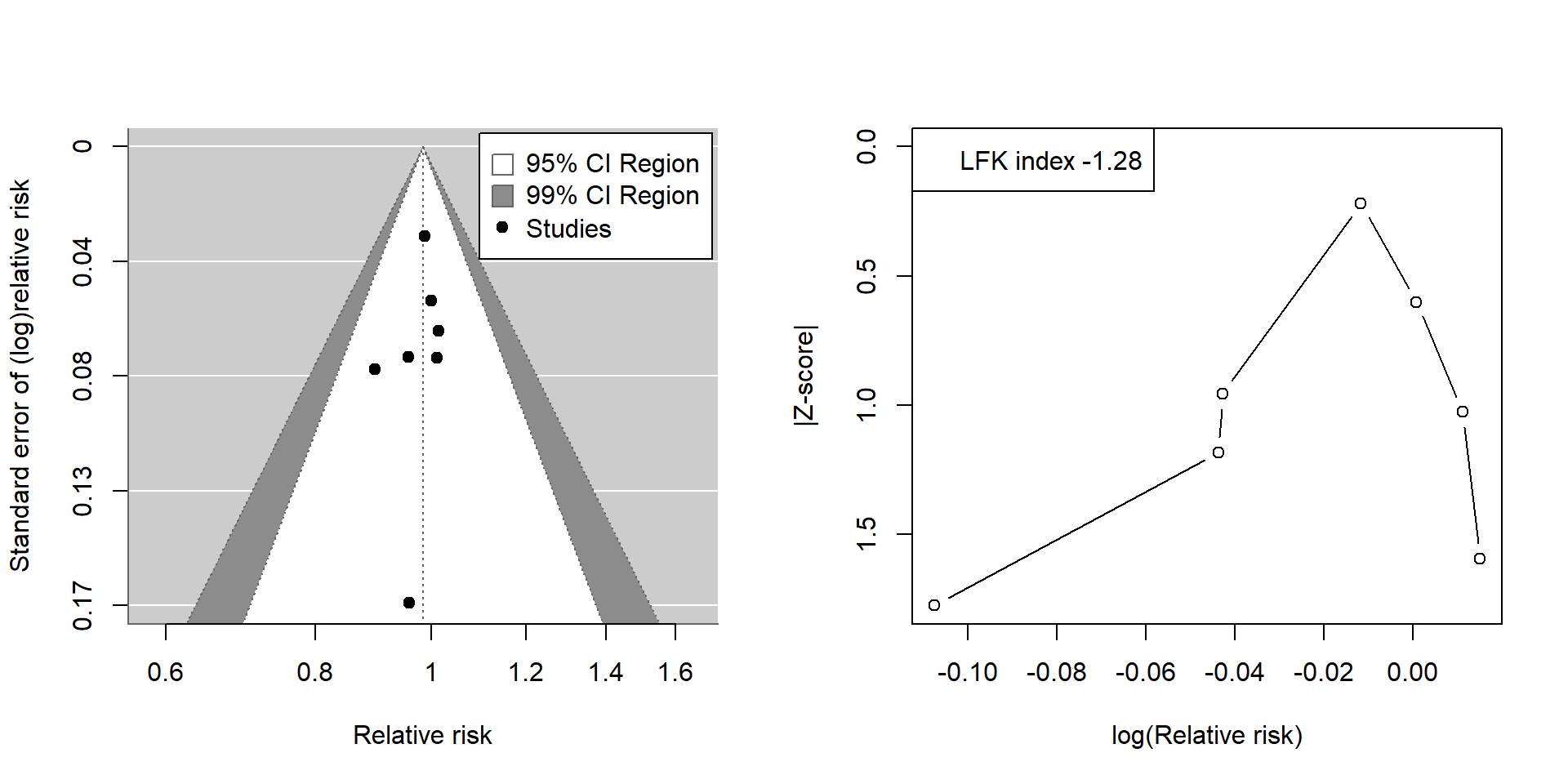


**Supplemental figure 27.** Contour-enhanced funnel plot (left) and Doi plot (right) for studies of the association between low-fat fermented dairy intake and prediabetes risk. Each dot indicates a study population. Egger’s test, P=0.67. LFK index = -1.28 indicating minor asymmetry.


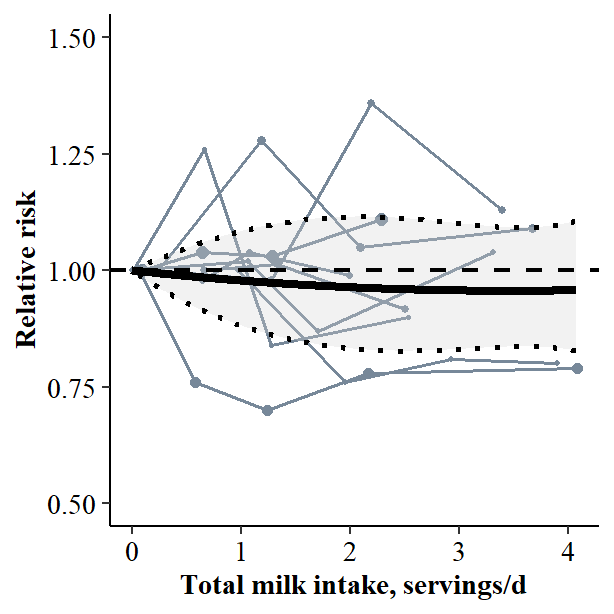

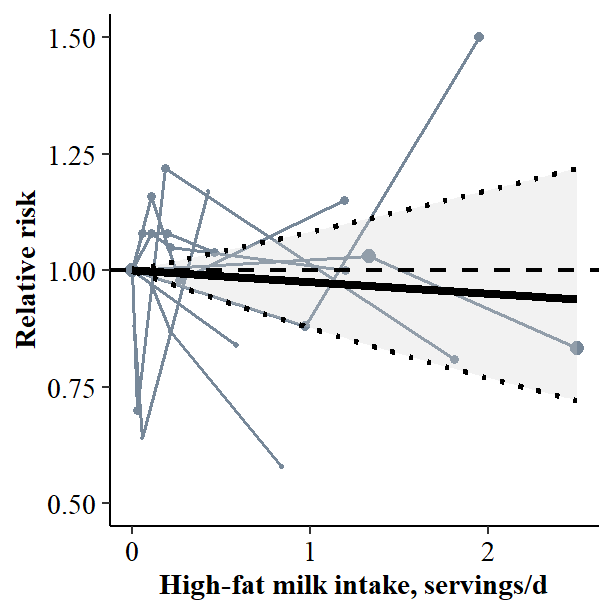

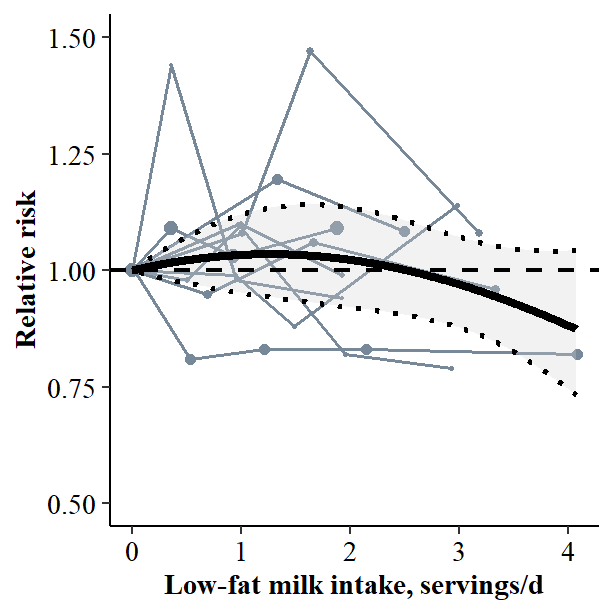


**Supplemental figure 28.** Spaghetti plot based on dose-response meta-analysis including 6 studies and 9 cohorts (6,653 cases among 95,844 participants) for the associations between **total milk** (lowest RR at 3.5 servings/day: 0.96, 95%CI 0.84-1.09, I^2^ = 37%)(left), **high-fat milk** (RR servings/day: 0.97 95%CI 0.88-1.08, I^2^ = 59%)(middle), and **low-fat milk** (lowest RR at 4.1 serving/day: 0.87, 95%CI 0.73-1.04, I^2^ = 30%)(right) intake and prediabetes risk. The solid black line represents the pooled RR at each quantity of intake. The light grey colored area between the dotted black lines indicates the 95% confidence interval. The dashed grey line at RR = 1.00 represents the reference line. Each solid grey line represents a cohort with circles placed at the cohort-specific RRs at the corresponding intake level. The area of the circle is proportional to the study-specific weight. The associations were adjusted for age, sex, energy intake, educational level, smoking behavior, physical activity, alcohol intake, family history of diabetes, intake of food groups, waist circumference or BMI, hypertension, and dyslipidemia. A serving size of milk was 150 g.


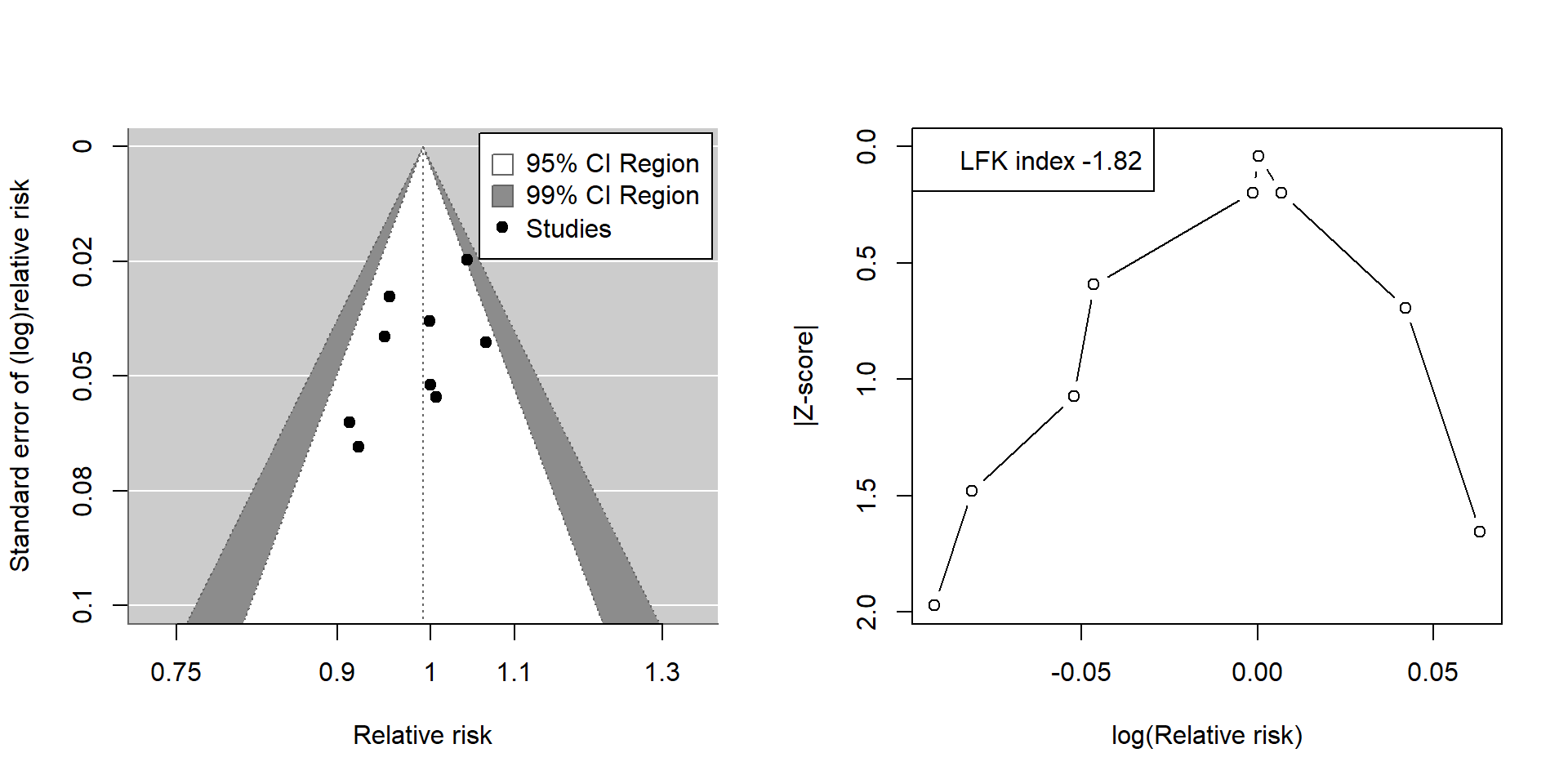


**Supplemental figure 29.** Contour-enhanced funnel plot (left) and Doi plot (right) for studies of the association between total milk intake and prediabetes risk. Each dot indicates a study population. Egger’s test, P=0.13. LFK index = -1.82 indicating minor asymmetry.


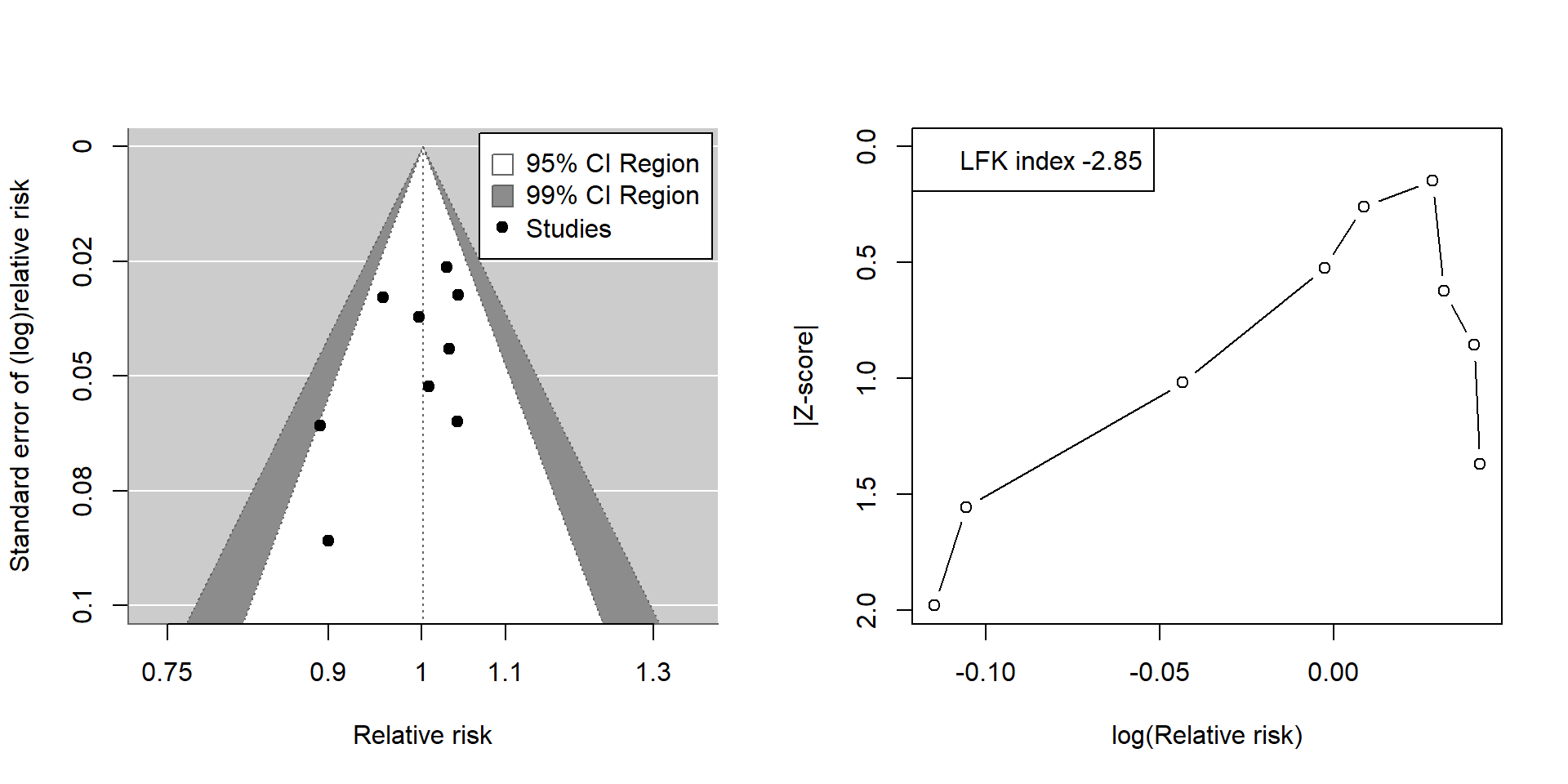


**Supplemental figure 30.** Contour-enhanced funnel plot (left) and Doi plot (right) for studies of the association between low-fat milk intake and prediabetes risk. Each dot indicates a study population. Egger’s test, P=0.11. LFK index = 1.69 indicating minor asymmetry.


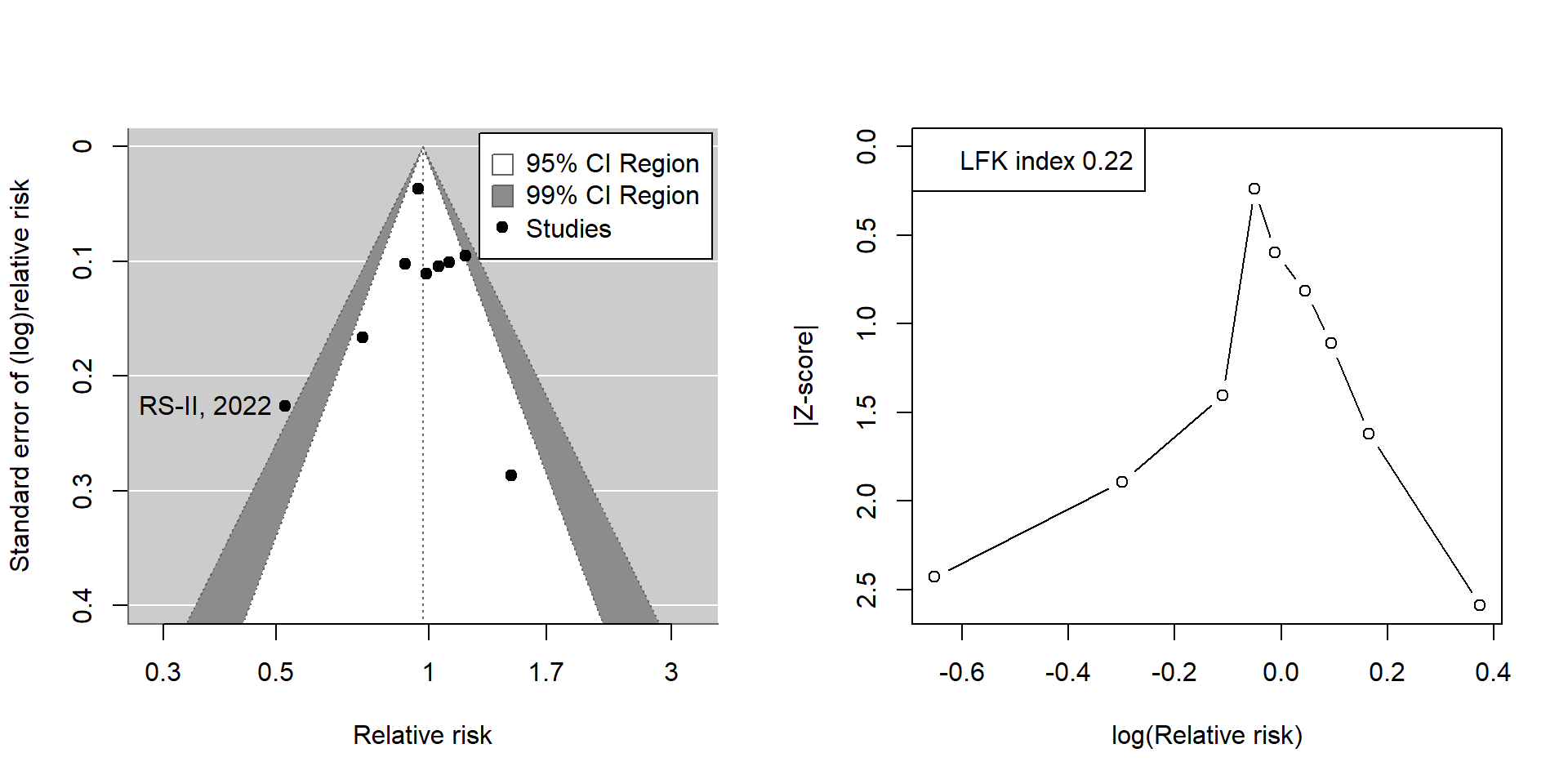


**Supplemental figure 31.** Contour-enhanced funnel plot (left) and Doi plot (right) for studies of the association between high-fat milk intake and prediabetes risk. Each dot indicates a study population. Egger’s test, P=0.45. LFK index = 0.22 indicating no asymmetry.


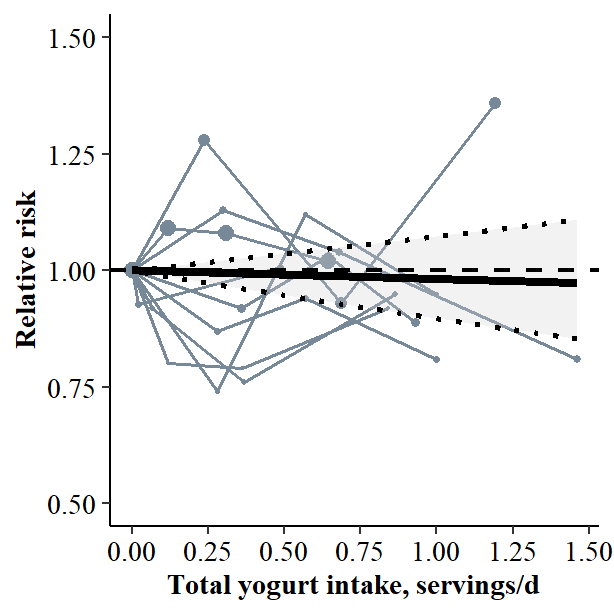

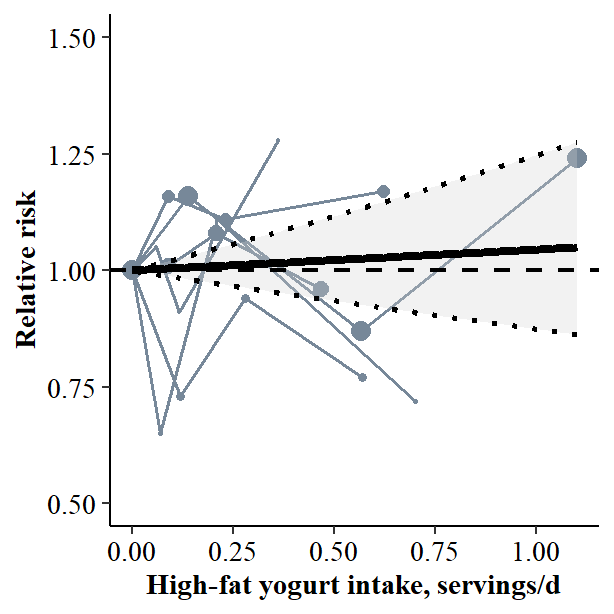

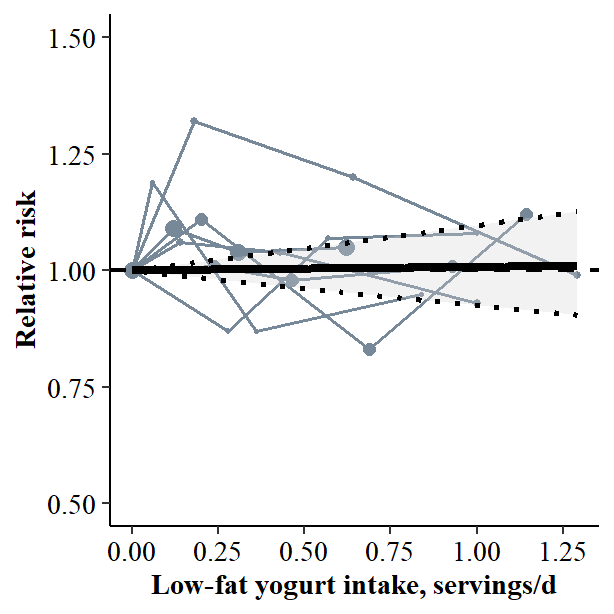


**Supplemental figure 32.** Spaghetti plot based on dose-response meta-analysis for the associations between **total yogurt** (RR servings/day: 0.98, 95%CI 0.90-1.07, I^2^ = 8%, including 6 studies and 9 cohorts, 6,653 cases among 95,844 participants)(left), **high-fat yogurt** (RR servings/day: 1.04 95%CI 0.87-1.25, I^2^ = 21%, including 4 studies and 7 cohorts, 4,986 cases among 89,089 participants)(middle), and **low-fat yogurt** (RR serving/day: 0.97, 95%CI 0.82-1.15, I^2^ = 0%, including 4 studies and 7 cohorts, 4,986 cases among 89,089 participants)(right) intake and prediabetes risk. The solid black line represents the pooled RR at each quantity of intake. The light grey colored area between the dotted black lines indicates the 95% confidence interval. The dashed grey line at RR = 1.00 represents the reference line. Each solid grey line represents a cohort with circles placed at the cohort-specific RRs at the corresponding intake level. The area of the circle is proportional to the study-specific weight. The associations were adjusted for age, sex, energy intake, educational level, smoking behavior, physical activity, alcohol intake, family history of diabetes, intake of food groups, waist circumference or BMI, hypertension, and dyslipidemia. A serving size of yogurt was 150 g.


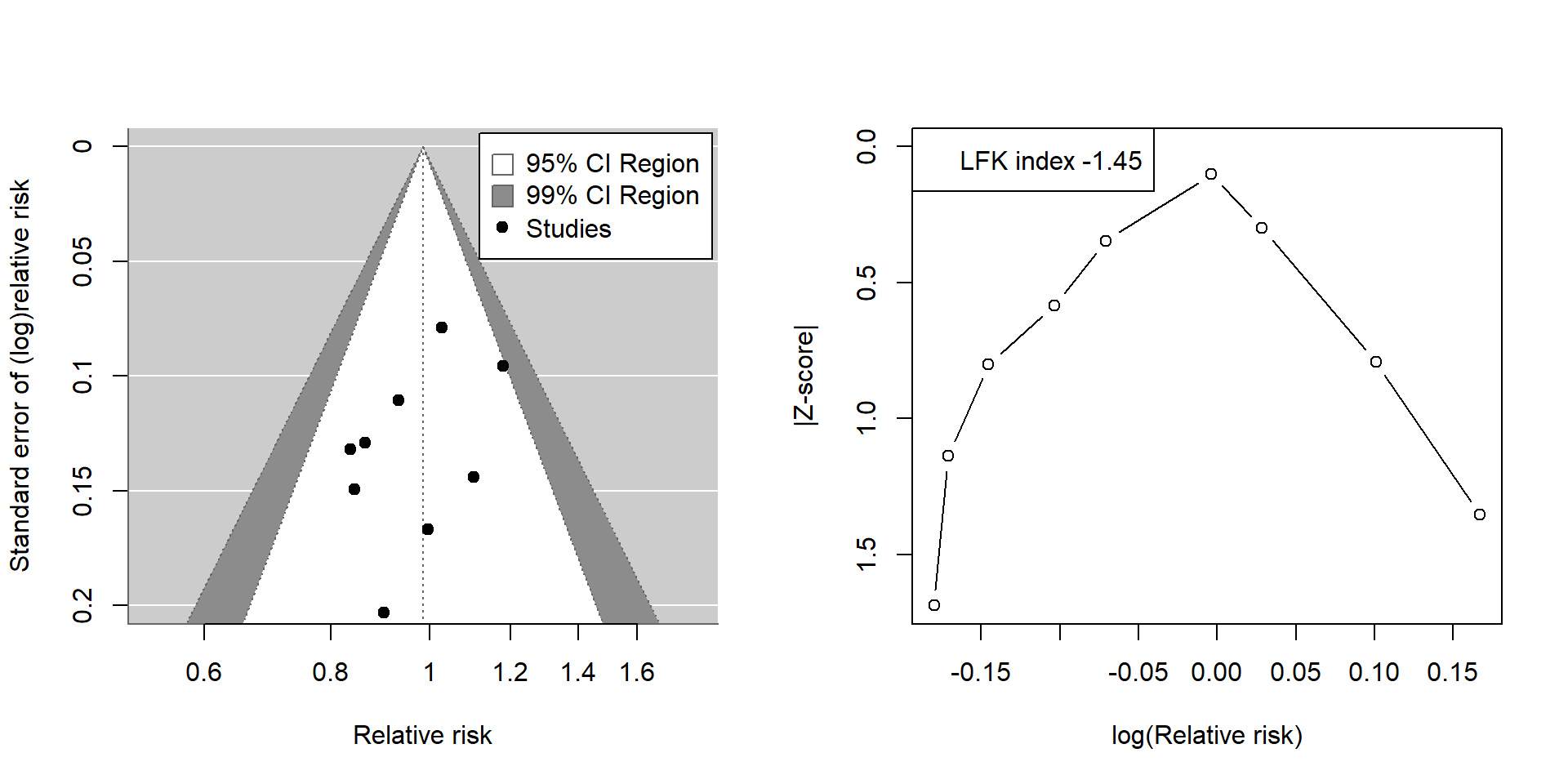


**Supplemental figure 33.** Contour-enhanced funnel plot (left) and Doi plot (right) for studies of the association between total yogurt intake and prediabetes risk. Each dot indicates a study population. Egger’s test, P=0.16. LFK index = -1.45 indicating minor asymmetry.


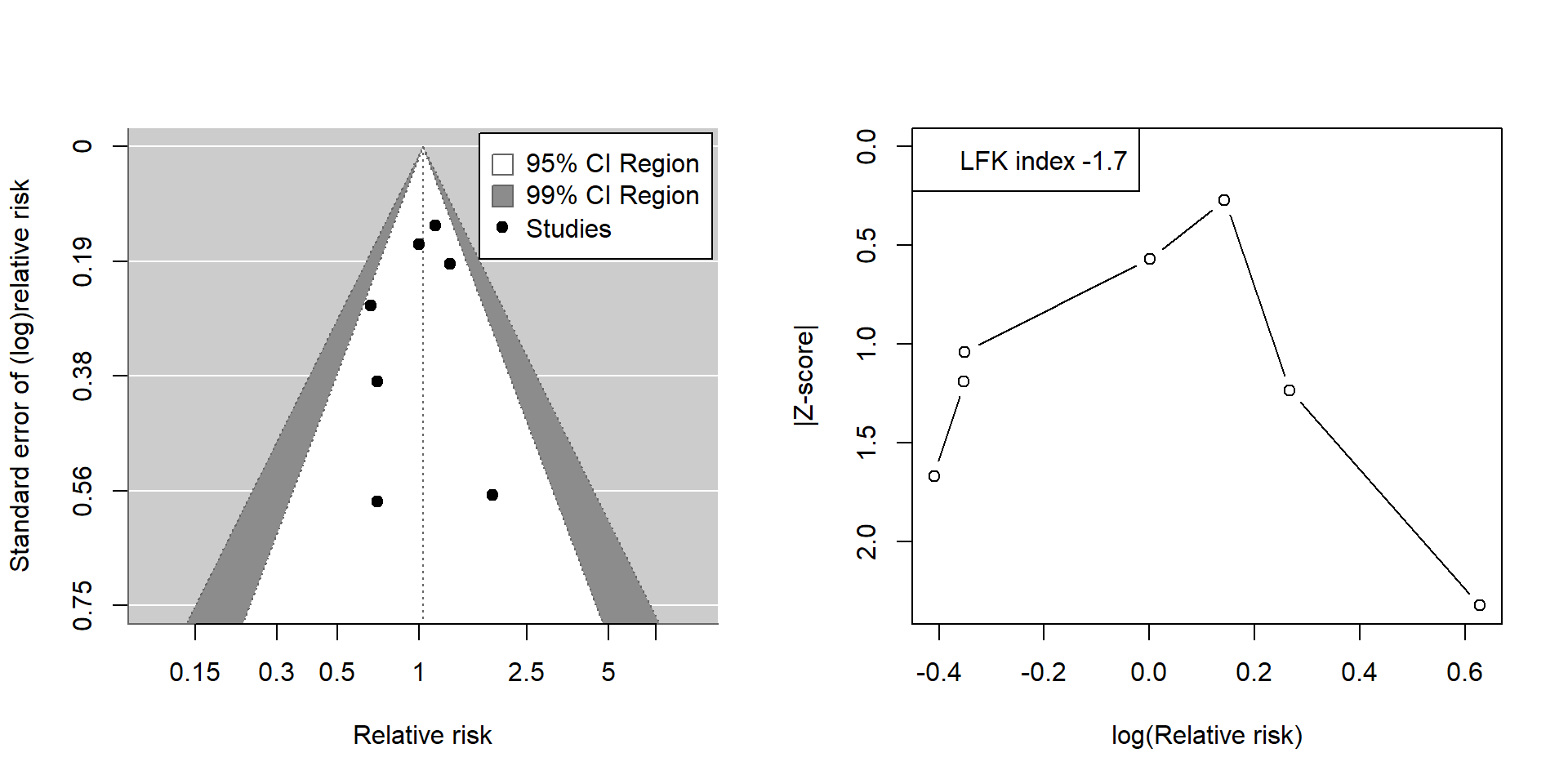


**Supplemental figure 34.** Contour-enhanced funnel plot (left) and Doi plot (right) for studies of the association between high-fat yogurt intake and prediabetes risk. Each dot indicates a study population. Egger’s test, P=0.50. LFK index = -1.70 indicating minor asymmetry.


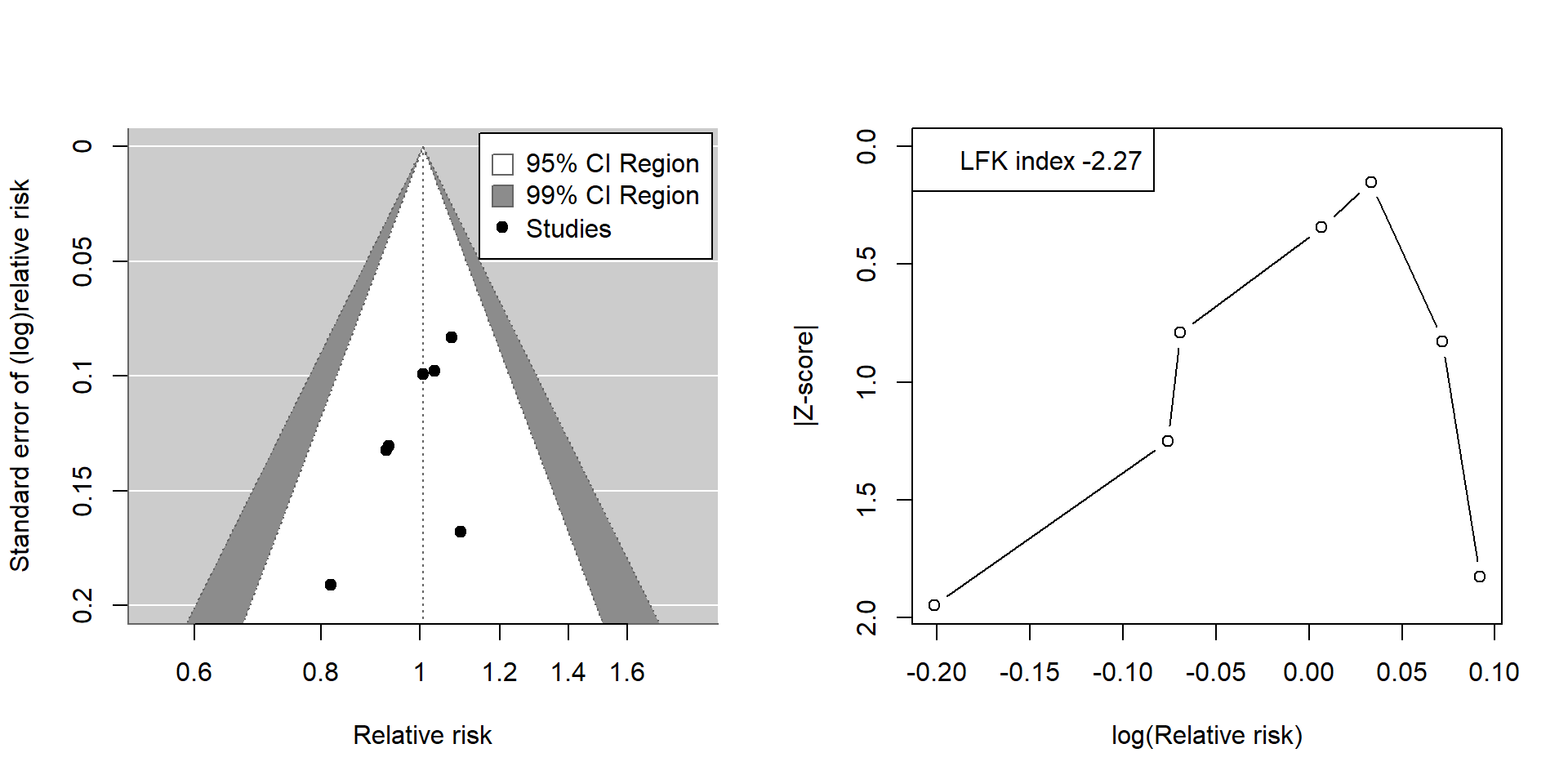


**Supplemental figure 35.** Contour-enhanced funnel plot (left) and Doi plot (right) for studies of the association between low-fat yogurt intake and prediabetes risk. Each dot indicates a study population. Egger’s test, P=0.27. LFK index = -2.27 indicating major asymmetry.


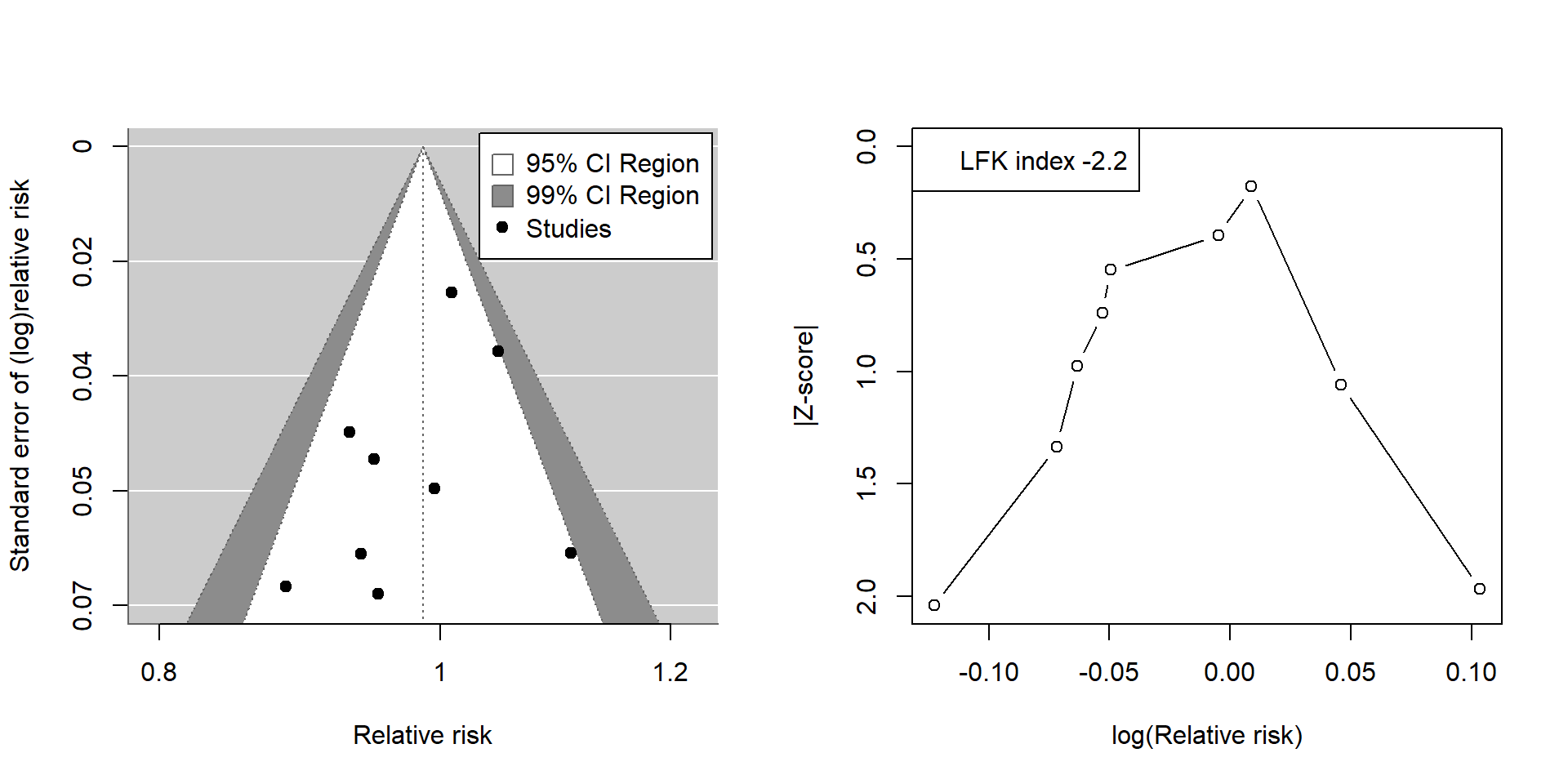


**Supplemental figure 36.** Contour-enhanced funnel plot (left) and Doi plot (right) for studies of the association between total cheese intake and prediabetes risk. Each dot indicates a study population. Egger’s test, P=0.24. LFK index = -2.2 indicating major asymmetry.


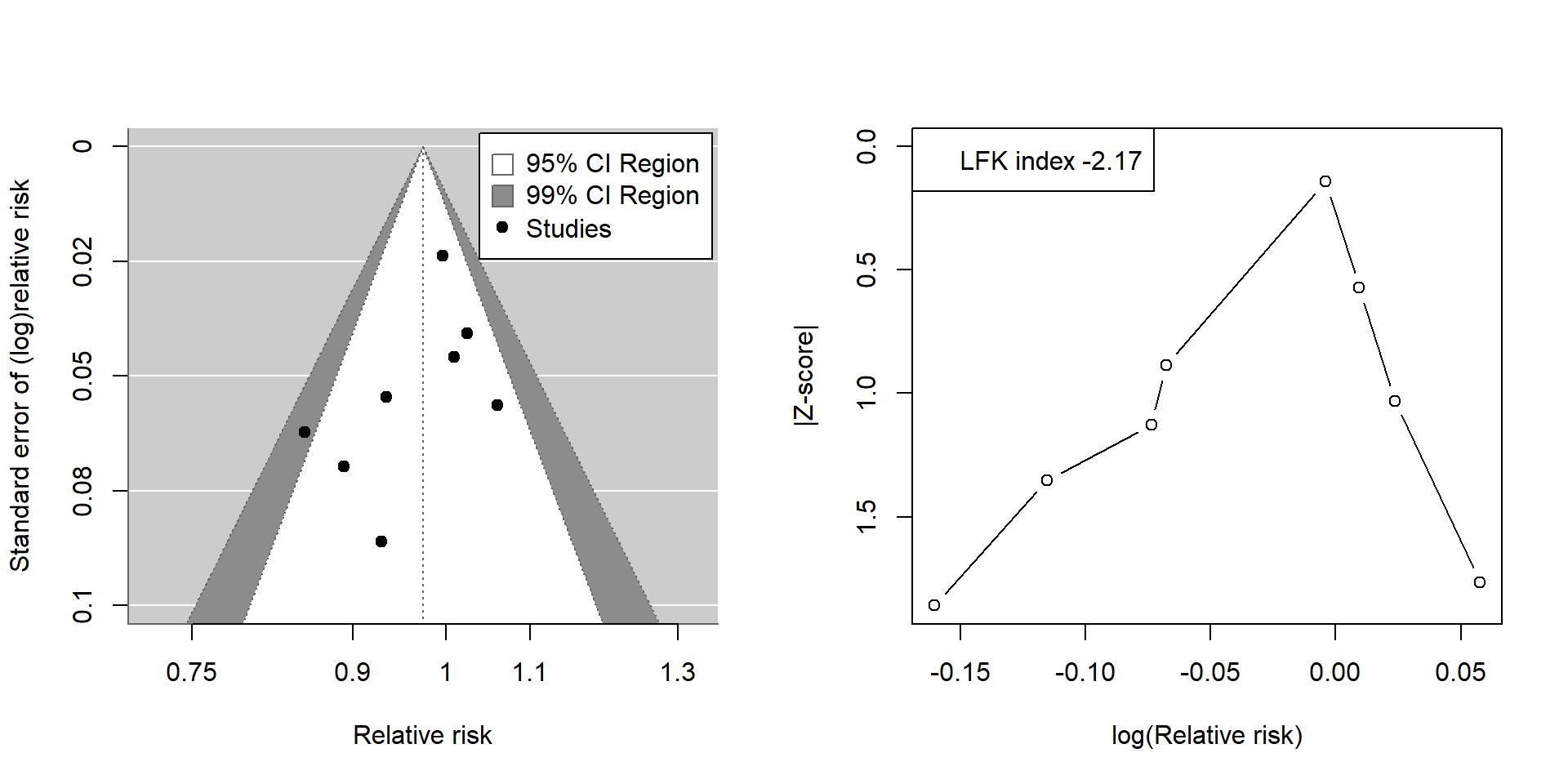


**Supplemental figure 37.** Contour-enhanced funnel plot (left) and Doi plot (right) for studies of the association between high-fat cheese intake and prediabetes risk. Each dot indicates a study population. Egger’s test, P=0.12. LFK index = -2.17 indicating major asymmetry.


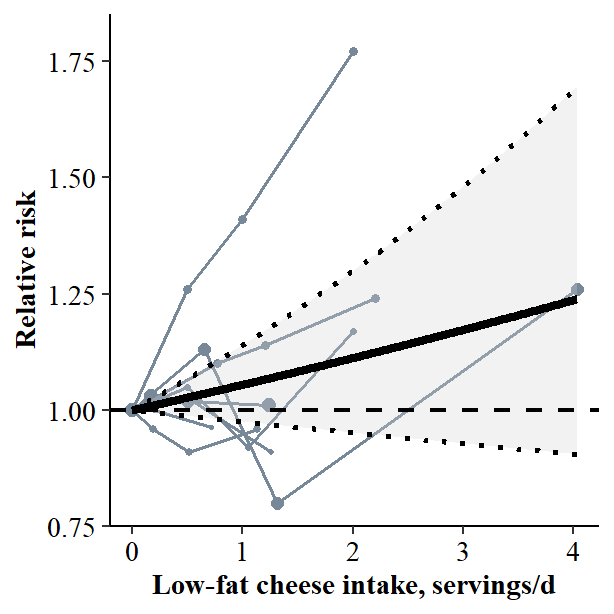


**Supplemental figure 38.** Spaghetti plot based on dose-response meta-analysis including 5 studies and 8 cohorts (5,751 cases among 93,977 participants) for the associations between l**ow-fat cheese** and prediabetes risk (RR per serving/day: 1.05, 95%CI 0.98-1.14, I^2^ = 48%). The solid black line represents the pooled RR at each quantity of intake. The light grey colored area between the dotted black lines indicates the 95% confidence interval. The dashed grey line at RR = 1.00 represents the reference line. Each solid grey line represents a cohort with circles placed at the cohort-specific RRs at the corresponding intake level. The area of the circle is proportional to the study-specific weight. The associations were adjusted for age, sex, energy intake, educational level, smoking behavior, physical activity, alcohol intake, family history of diabetes, intake of food groups, waist circumference or BMI, hypertension, and dyslipidemia. A serving size of cheese was 20 g.


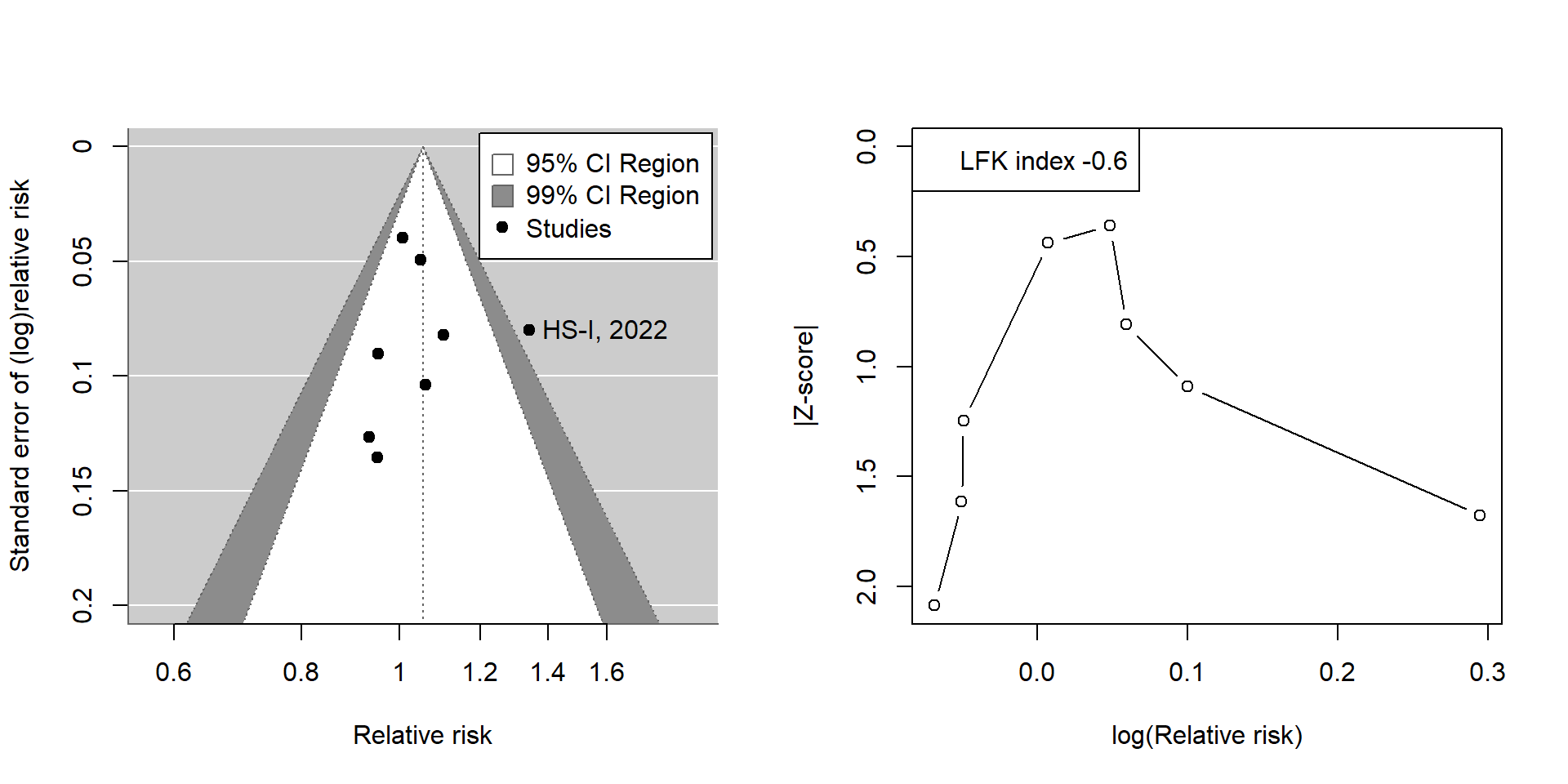


**Supplemental figure 39.** Contour-enhanced funnel plot (left) and Doi plot (right) for studies of the association between low-fat cheese intake and prediabetes risk. Each dot indicates a study population. Egger’s test, P=0.69. LFK index = -0.6 indicating no asymmetry.


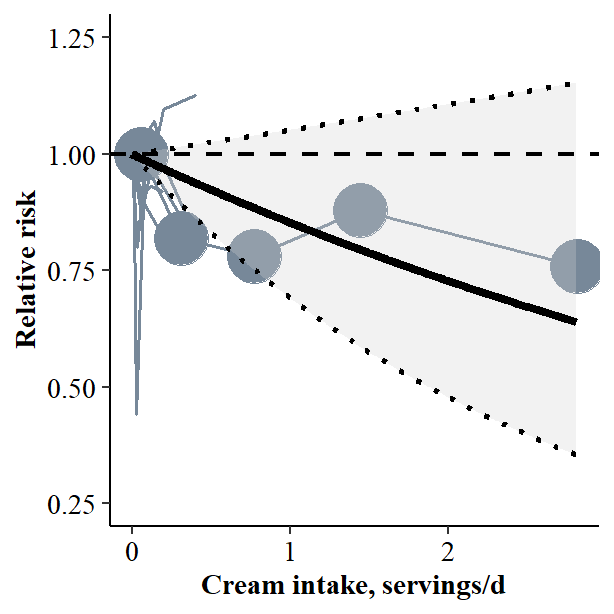


**Supplemental figure 40.** Spaghetti plot based on dose-response meta-analysis including 5 studies and 8 cohorts (5,888 cases among 90,953 participants) for the associations between **cream** intake and prediabetes risk (RR per serving/day: 0.85, 95%CI 0.69-1.05, I^2^ = 0%). The solid black line represents the pooled RR at each quantity of intake. The light grey colored area between the dotted black lines indicates the 95% confidence interval. The dashed grey line at RR = 1.00 represents the reference line. Each solid grey line represents a cohort with circles placed at the cohort-specific RRs at the corresponding intake level. The area of the circle is proportional to the study-specific weight. The associations were adjusted for age, sex, energy intake, educational level, smoking behavior, physical activity, alcohol intake, family history of diabetes, intake of food groups, waist circumference or BMI, hypertension, and dyslipidemia. A serving size of cream was 15 g.


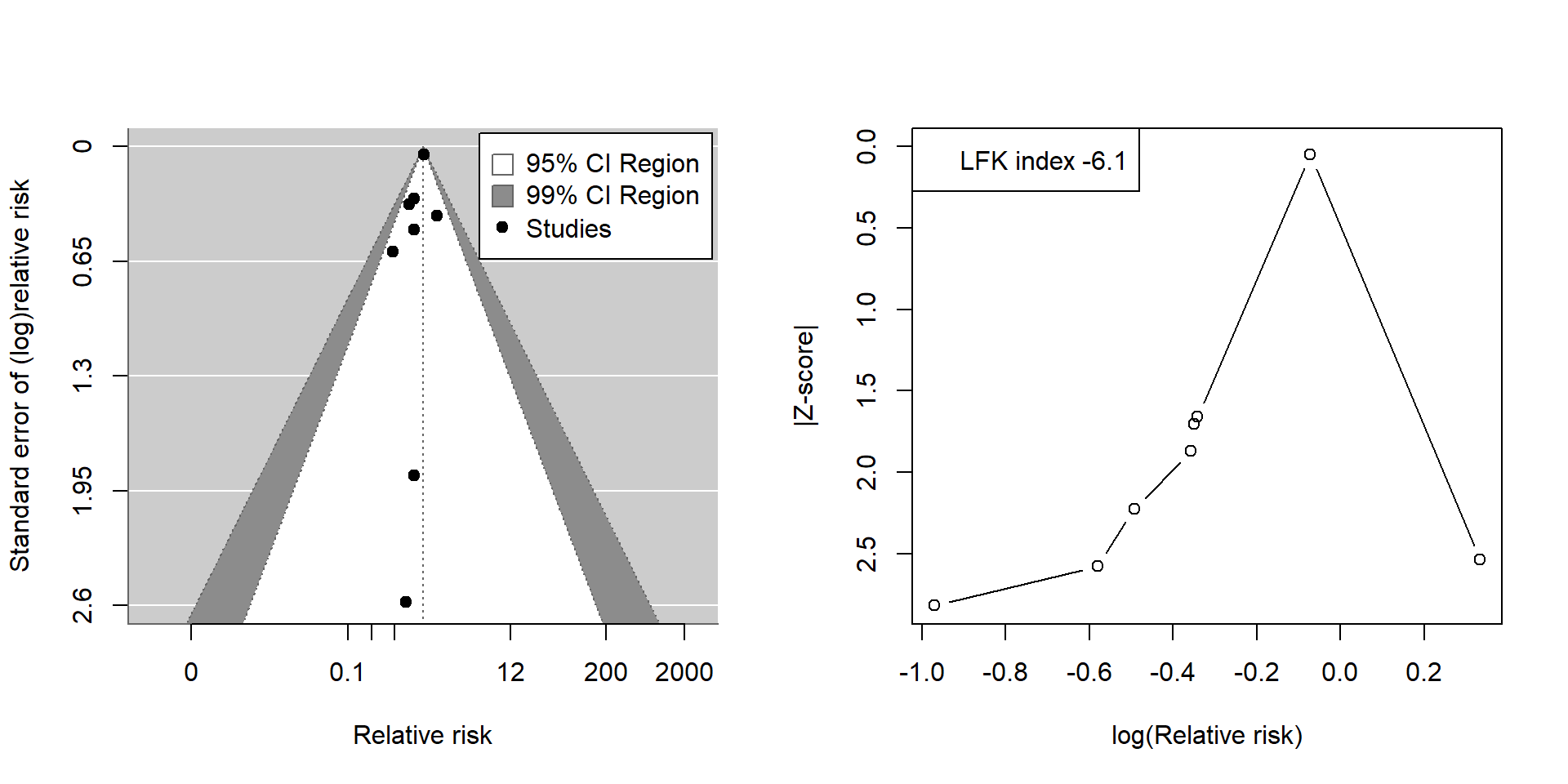


**Supplemental figure 41.** Contour-enhanced funnel plot (left) and Doi plot (right) for studies of the association between cream intake and prediabetes risk. Each dot indicates a study population. Egger’s test, P=0.19. LFK index = -6.1 indicating major asymmetry.


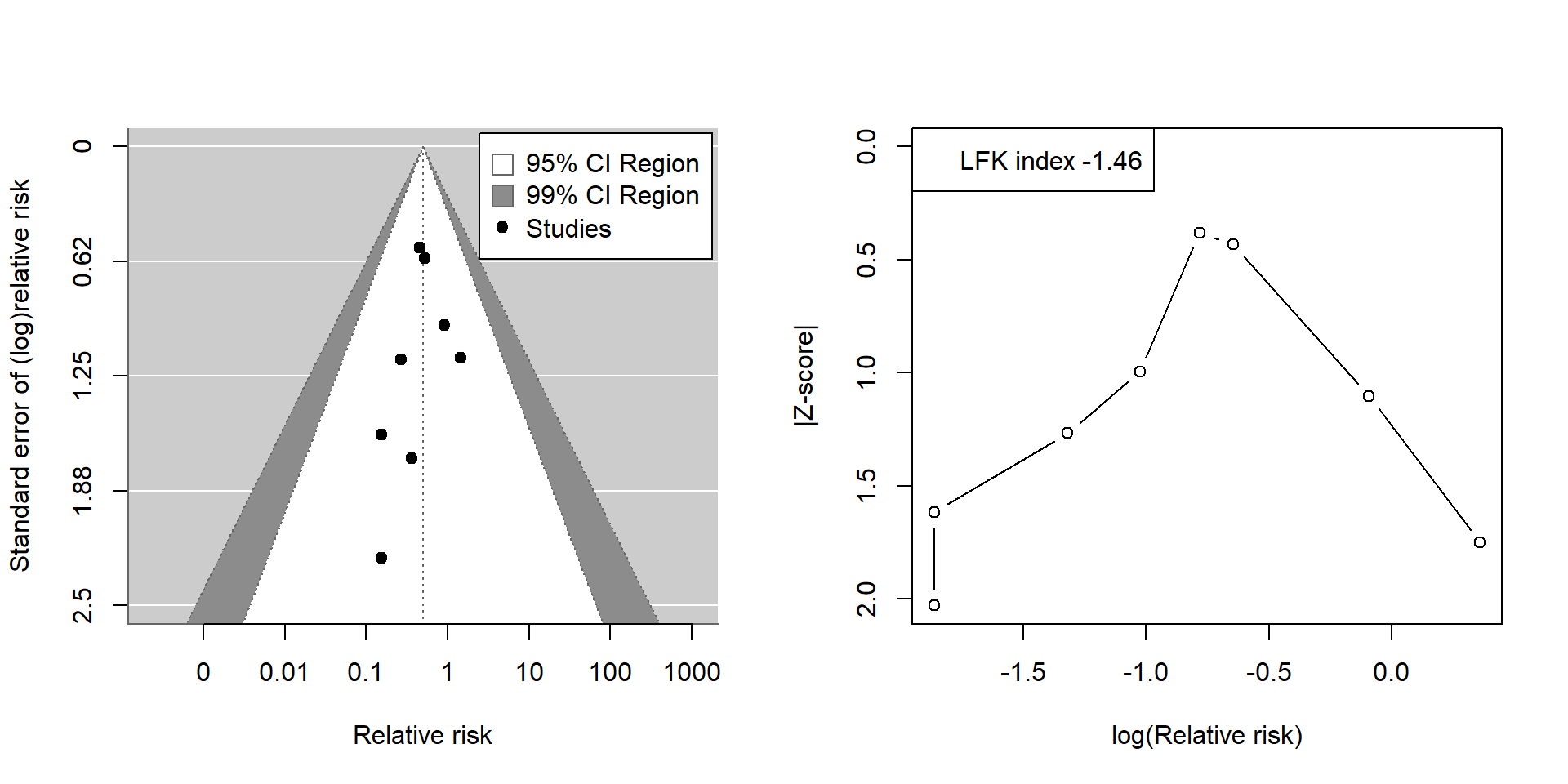


**Supplemental figure 42.** Contour-enhanced funnel plot (left) and Doi plot (right) for studies of the association between ice cream intake and prediabetes risk. Each dot indicates a study population. Egger’s test, P=0.66. LFK index = -1.46 indicating minor asymmetry.

# References

1. Adherence to predefined dietary patterns and incident type 2 diabetes in European populations: EPIC-InterAct Study. Diabetologia 2014;57(2):321-33. doi: 10.1007/s00125-013-3092-9.
2. Babio N, Becerra-Tomás N, Martínez-González M, Corella D, Estruch R, Ros E, Sayón-Orea C, Fitó M, Serra-Majem L, Arós F, et al. Consumption of Yogurt, Low-Fat Milk, and Other Low-Fat Dairy Products Is Associated with Lower Risk of Metabolic Syndrome Incidence in an Elderly Mediterranean Population. J Nutr 2015;145(10):2308-16. doi: 10.3945/jn.115.214593.
3. Bahadoran Z, Mirmiran P, Mirzaee S, Delshad H, Azizi F. The effect of consumption of unhealthy snacks on diet and the risk of metabolic syndrome in adults: Tehran lipid and glucose study, Iran. Journal of Kerman University of Medical Sciences 2014;21(6):485-97. doi: .
4. Baik I, Lee M, Jun NR, Lee JY, Shin C. A healthy dietary pattern consisting of a variety of food choices is inversely associated with the development of metabolic syndrome. Nutr Res Pract 2013;7(3):233-41. doi: 10.4162/nrp.2013.7.3.233.
5. Beydoun MA, Fanelli-Kuczmarski MT, Beydoun HA, Dore GA, Canas JA, Evans MK, Zonderman AB. Dairy product consumption and its association with metabolic disturbance in a prospective study of urban adults. Br J Nutr 2018;119(6):706-19. doi: 10.1017/s0007114518000028.
6. Bhavadharini B, Dehghan M, Mente A, Rangarajan S, Sheridan P, Mohan V, Iqbal R, Gupta R, Lear S, Wentzel-Viljoen E, et al. Association of dairy consumption with metabolic syndrome, hypertension and diabetes in 147 812 individuals from 21 countries. BMJ Open Diabetes Res Care 2020;8(1). doi: 10.1136/bmjdrc-2019-000826.
7. Brouwer-Brolsma EM, van Woudenbergh GJ, Oude Elferink SJ, Singh-Povel CM, Hofman A, Dehghan A, Franco OH, Feskens EJ. Intake of different types of dairy and its prospective association with risk of type 2 diabetes: The Rotterdam Study. Nutr Metab Cardiovasc Dis 2016;26(11):987-95. doi: 10.1016/j.numecd.2016.08.003.
8. Brunner EJ, Mosdøl A, Witte DR, Martikainen P, Stafford M, Shipley MJ, Marmot MG. Dietary patterns and 15-y risks of major coronary events, diabetes, and mortality. Am J Clin Nutr 2008;87(5):1414-21. doi: 10.1093/ajcn/87.5.1414.
9. Buscemi S, Corleo D, Buscemi C, azzo C, Borzì AM, Barile AM, Rosafio G, Ciaccio M, Caldarella R, Meli F, et al. Influence of Habitual Dairy Food Intake on LDL Cholesterol in a Population-Based Cohort. Nutrients 2021;13(2). doi: 10.3390/nu13020593.
10. Chen Z, Kh, pur N, Desjardins C, Wang L, Monteiro CA, Rossato SL, Fung TT, Manson JE, Willett WC, et al. Ultra-Processed Food Consumption and Risk of Type 2 Diabetes: Three Large Prospective U.S. Cohort Studies. Diabetes Care 2023;46(7):1335-44. doi: 10.2337/dc22-1993.
11. Cheraghi Z, Mirmiran P, Mansournia MA, Moslehi N, Khalili D, Nedjat S. The association between nutritional exposures and metabolic syndrome in the Tehran Lipid and Glucose Study (TLGS): a cohort study. Public Health 2016;140:163-71. doi: 10.1016/j.puhe.2016.07.003.
12. Cho GJ, Park HT, Shin JH, Hur JY, Kim YT, Kim SH, Lee KW, Kim T. Calcium intake is inversely associated with metabolic syndrome in postmenopausal women: Korea National Health and Nutrition Survey, 2001 and 2005. MENOPAUSE-THE JOURNAL OF THE NORTH AMERICAN MENOPAUSE SOCIETY 2009;16(5):992-7. doi: 10.1097/gme.0b013e31819e23cb.
13. Choi HK, Willett WC, Stampfer MJ, Rimm E, Hu FB. Dairy consumption and risk of type 2 diabetes mellitus in men: a prospective study. Arch Intern Med 2005;165(9):997-1003. doi: 10.1001/archinte.165.9.997.
14. Delavar MA, Lye MS, Khor GL, Hassan S, Hanachi P. Dietary patterns and the metabolic syndrome in middle aged women, Babol, Iran. ASIA PACIFIC JOURNAL OF CLINICAL NUTRITION 2009;18(2):285-92. doi: .
15. Díaz-López A, Bulló M, Martínez-González MA, Corella D, Estruch R, Fitó M, Gómez-Gracia E, Fiol M, García de la Corte FJ, Ros E, et al. Dairy product consumption and risk of type 2 diabetes in an elderly Spanish Mediterranean population at high cardiovascular risk. Eur J Nutr 2016;55(1):349-60. doi: 10.1007/s00394-015-0855-8.
16. Dominguez LJ, Bes-Rastrollo M, Basterra-Gortari FJ, Gea A, Barbagallo M, Martínez-González MA. Association of a Dietary Score with Incident Type 2 Diabetes: The Dietary-Based Diabetes-Risk Score (DDS). PLoS One 2015;10(11):e0141760. doi: 10.1371/journal.pone.0141760.
17. Dow C, Balkau B, Bonnet F, Mancini F, Rajaobelina K, Shaw J, Magliano DJ, Fagherazzi G. Strong adherence to dietary and lifestyle recommendations is associated with decreased type 2 diabetes risk in the AusDiab cohort study. Prev Med 2019;123:208-16. doi: 10.1016/j.ypmed.2019.03.006.
18. Drehmer M, Odegaard AO, Schmidt MI, Duncan BB, Cardoso LO, Matos SMA, Molina M, Barreto SM, Pereira MA. Brazilian dietary patterns and the dietary approaches to stop hypertension (DASH) diet-relationship with metabolic syndrome and newly diagnosed diabetes in the ELSA-Brasil study. Diabetol Metab Syndr 2017;9:13. doi: 10.1186/s13098-017-0211-7.
19. Drehmer M, Pereira MA, Schmidt MI, Alvim S, Lotufo PA, Luft VC, Duncan BB. Total and Full-Fat, but Not Low-Fat, Dairy Product Intakes are Inversely Associated with Metabolic Syndrome in Adults. J Nutr 2016;146(1):81-9. doi: 10.3945/jn.115.220699.
20. Drouin-Chartier JP, Li Y, Ardisson Korat AV, Ding M, Lamarche B, Manson JE, Rimm EB, Willett WC, Hu FB. Changes in dairy product consumption and risk of type 2 diabetes: results from 3 large prospective cohorts of US men and women. Am J Clin Nutr 2019;110(5):1201-12. doi: 10.1093/ajcn/nqz180.
21. Duffey KJ, Gordon-Larsen P, Steffen LM, Jacobs DR, Jr., Popkin BM. Drinking caloric beverages increases the risk of adverse cardiometabolic outcomes in the Coronary Artery Risk Development in Young Adults (CARDIA) Study. Am J Clin Nutr 2010;92(4):954-9. doi: 10.3945/ajcn.2010.29478.
22. Duffey KJ, Steffen LM, Van Horn L, Jacobs DR, Jr., Popkin BM. Dietary patterns matter: diet beverages and cardiometabolic risks in the longitudinal Coronary Artery Risk Development in Young Adults (CARDIA) Study. Am J Clin Nutr 2012;95(4):909-15. doi: 10.3945/ajcn.111.026682.
23. Elwood PC, Pickering JE, Fehily AM. Milk and dairy consumption, diabetes and the metabolic syndrome: the Caerphilly prospective study. J Epidemiol Community Health 2007;61(8):695-8. doi: 10.1136/jech.2006.053157.
24. Ericson U, Brunkwall L, Alves Dias J, Drake I, Hellstr, S, Gullberg B, Sonestedt E, Nilsson PM, Wirfält E, et al. Food patterns in relation to weight change and incidence of type 2 diabetes, coronary events and stroke in the Malmö Diet and Cancer cohort. Eur J Nutr 2019;58(5):1801-14. doi: 10.1007/s00394-018-1727-9.
25. Ericson U, Hellstr, S, Brunkwall L, Schulz CA, Sonestedt E, Wallström P, Gullberg B, Wirfält E, Orho-Mel, et al. Food sources of fat may clarify the inconsistent role of dietary fat intake for incidence of type 2 diabetes. Am J Clin Nutr 2015;101(5):1065-80. doi: 10.3945/ajcn.114.103010.
26. Forouhi NG, Koulman A, Sharp SJ, Imamura F, Kroger J, Schulze MB, Crowe FL, Huerta JM, Guevara M, Beulens JWJ, et al. Differences in the prospective association between individual plasma phospholipid saturated fatty acids and incident type 2 diabetes: the EPIC-InterAct case-cohort study. LANCET DIABETES & ENDOCRINOLOGY 2014;2(10):810-8. doi: 10.1016/S2213-8587(14)70146-9.
27. Fumeron F, Lamri A, Abi Khalil C, Jaziri R, Porchay-Balderelli I, Lantieri O, Vol S, Balkau B, Marre M, Data from the Epidemiological Study on the Insulin Resistance Syndrome Study G. Dairy consumption and the incidence of hyperglycemia and the metabolic syndrome: results from a french prospective study, Data from the Epidemiological Study on the Insulin Resistance Syndrome (DESIR). Diabetes Care 2011;34(4):813-7. doi: 10.2337/dc10-1772.
28. Fung GJ, Steffen LM, Zhou X, Harnack L, Tang W, Lutsey PL, Loria CM, Reis JP, Van Horn LV. Vitamin D intake is inversely related to risk of developing metabolic syndrome in African American and white men and women over 20 y: the Coronary Artery Risk Development in Young Adults study. Am J Clin Nutr 2012;96(1):24-9. doi: 10.3945/ajcn.112.036863. Epub 2012 May 30.
29. Gao M, Jebb SA, Aveyard P, Ambrosini GL, Perez-Cornago A, Papier K, Carter J, Piernas C. Associations Between Dietary Patterns and Incident Type 2 Diabetes: Prospective Cohort Study of 120,343 UK Biobank Participants. Diabetes Care 2022;45(6):1315-25. doi: 10.2337/dc21-2258.
30. Grantham NM, Magliano DJ, Hodge A, Jowett J, Meikle P, Shaw JE. The association between dairy food intake and the incidence of diabetes in Australia: the Australian Diabetes Obesity and Lifestyle Study (AusDiab). Public Health Nutr 2013;16(2):339-45. doi: 10.1017/S1368980012001310.
31. Hardy DS, Racette SB, Garvin JT, Gebrekristos HT, Mersha TB. Ancestry specific associations of a genetic risk score, dietary patterns and metabolic syndrome: a longitudinal ARIC study. BMC Med Genomics 2021;14(1):118. doi: 10.1186/s12920-021-00961-8.
32. Hong X, Xu F, Wang Z, Liang Y, Li J. Dietary patterns and the incidence of hyperglyacemia in China. Public Health Nutr 2016;19(1):131-41. doi: 10.1017/s1368980015000774.
33. Imamura F, Schulze MB, Sharp SJ, Guevara M, Romaguera D, Bendinelli B, Salamanca-Fernández E, Ardanaz E, Arriola L, Aune D, et al. Estimated Substitution of Tea or Coffee for Sugar-Sweetened Beverages Was Associated with Lower Type 2 Diabetes Incidence in Case-Cohort Analysis across 8 European Countries in the EPIC-InterAct Study. J Nutr 2019;149(11):1985-93. doi: 10.1093/jn/nxz156.
34. Jacobs S, Kroeger J, Schulze MB, Frank LK, Franke AA, Cheng I, Monroe KR, Haiman CA, Kolonel LN, Wilkens LR, et al. Dietary Patterns Derived by Reduced Rank Regression Are Inversely Associated with Type 2 Diabetes Risk across 5 Ethnic Groups in the Multiethnic Cohort. Curr Dev Nutr 2017;1(5):e000620. doi: 10.3945/cdn.117.000620.
35. Jeon J, Jang J, Park K. Effects of Consuming Calcium-Rich Foods on the Incidence of Type 2 Diabetes Mellitus. Nutrients 2018;11(1). doi: 10.3390/nu11010031.
36. Johansson I, Esberg A, Nilsson LM, Jansson JH, Wennberg P, Winkvist A. Dairy Product Intake and Cardiometabolic Diseases in Northern Sweden: A 33-Year Prospective Cohort Study. Nutrients 2019;11(2). doi: 10.3390/nu11020284.
37. Johansson I, Nilsson LM, Esberg A, Jansson JH, Winkvist A. Dairy intake revisited - associations between dairy intake and lifestyle related cardio-metabolic risk factors in a high milk consuming population. Nutr J 2018;17(1):110. doi: 10.1186/s12937-018-0418-y.
38. Jung HJ, Han SN, Song S, Paik HY, Baik HW, Joung H. Association between adherence to the Korean Food Guidance System and the risk of metabolic abnormalities in Koreans. Nutr Res Pract 2011;5(6):560-8. doi: 10.4162/nrp.2011.5.6.560.
39. Keshavarz Z, Rahimlou M, Farjam M, Homayounfar R, Khodadost M, Abdollahi A, Tabrizi R. Non-alcoholic fatty liver disease and dairy products consumption: Results from FASA Persian cohort study. FRONTIERS IN NUTRITION 2022;9. doi: 10.3389/fnut.2022.962834.
40. Kim D, Kim J. Dairy consumption is associated with a lower incidence of the metabolic syndrome in middle-aged and older Korean adults: the Korean Genome and Epidemiology Study (KoGES). Br J Nutr 2017;117(1):148-60. doi: 10.1017/s000711451600444x.
41. Kim J, Kim M, Shin Y, Cho JH, Lee D, Kim Y. Association between Dietary Diversity Score and Metabolic Syndrome in Korean Adults: A Community-Based Prospective Cohort Study. Nutrients 2022;14(24). doi: 10.3390/nu14245298.
42. Kim Y, Kim YM, Shin MH, Koh SB, Kim HC, Kim MK. Empirically identified dietary patterns and metabolic syndrome risk in a prospective cohort study: The Cardiovascular Disease Association Study. CLINICAL NUTRITION 2022;41(10):2156-62. doi: 10.1016/j.clnu.2022.07.038.
43. Kirii K, Mizoue T, Iso H, Takahashi Y, Kato M, Inoue M, Noda M, Tsugane S, Japan Public Health Center-based Prospective Study G. Calcium, vitamin D and dairy intake in relation to type 2 diabetes risk in a Japanese cohort. Diabetologia 2009;52(12):2542-50. doi: 10.1007/s00125-009-1554-x.
44. Kouvari M, Panagiotakos DB, Chrysohoou C, Georgousopoulou EN, Yannakoulia M, Tousoulis D, Pitsavos C. Dairy products, surrogate markers, and cardiovascular disease; a sex-specific analysis from the ATTICA prospective study. Nutr Metab Cardiovasc Dis 2020;30(12):2194-206. doi: 10.1016/j.numecd.2020.07.037.
45. Lampousi AM, Carlsson S, Löfvenborg JE, Cabrera-Castro N, Chirlaque MD, Fagherazzi G, Franks PW, Hampe CS, Jakszyn P, Koulman A, et al. Interaction between plasma phospholipid odd-chain fatty acids and GAD65 autoantibodies on the incidence of adult-onset diabetes: the EPIC-InterAct case-cohort study. Diabetologia 2023;66(8):1460-71. doi: 10.1007/s00125-023-05948-x.
46. Lee KW, Woo HD, Cho MJ, Park JK, Kim SS. Identification of Dietary Patterns Associated with Incidence of Hyperglycemia in Middle-Aged and Older Korean Adults. NUTRIENTS 2019;11(8). doi: 10.3390/nu11081801.
47. Lerchbaum E, Giuliani A, Gruber HJ, Pieber TR, Obermayer-Pietsch B. Adult-type hypolactasia and calcium intake in polycystic ovary syndrome. Clin Endocrinol (Oxf) 2012;77(6):834-43. doi: 10.1111/j.1365-2265.2012.04334.x.
48. Li M, Shi Z. Dietary Pattern during 1991-2011 and Its Association with Cardio Metabolic Risks in Chinese Adults: The China Health and Nutrition Survey. Nutrients 2017;9(11). doi: 10.3390/nu9111218.
49. Liese AD, Weis KE, Schulz M, Tooze JA. Food intake patterns associated with incident type 2 diabetes: the Insulin Resistance Atherosclerosis Study. Diabetes Care 2009;32(2):263-8. doi: 10.2337/dc08-1325.
50. Lin R, Chien KL, Tsai MC, Wang YJ, Hsu LY. Association between a priori and a posteriori dietary patterns and the risk of type 2 diabetes: a representative cohort study in Taiwan. J Nutr Sci 2023;12:e16. doi: 10.1017/jns.2023.8. eCollection 2023.
51. Lin YH, Chang HT, Tseng YH, Lin MH, Chen YC, Yang HW, Chen TJ, Hwang SJ. Characteristics and Health Behavior of Newly Developed Metabolic Syndrome Among Community-Dwelling Elderly in Taiwan. INTERNATIONAL JOURNAL OF GERONTOLOGY 2013;7(2):90-6. doi: 10.1016/j.ijge.2012.07.003.
52. Liu M, Liu C, Zhang Z, Zhou C, Li Q, He P, Zhang Y, Li H, Qin X. Quantity and variety of food groups consumption and the risk of diabetes in adults: A prospective cohort study. Clin Nutr 2021;40(12):5710-7. doi: 10.1016/j.clnu.2021.10.003.
53. Liu S, Choi HK, Ford E, Song Y, Klevak A, Buring JE, Manson JE. A prospective study of dairy intake and the risk of type 2 diabetes in women. Diabetes Care 2006;29(7):1579-84. doi: 10.2337/dc06-0256.
54. Louie JC, Flood VM, Rangan AM, Burlutsky G, Gill TP, Gopinath B, Mitchell P. Higher regular fat dairy consumption is associated with lower incidence of metabolic syndrome but not type 2 diabetes. Nutr Metab Cardiovasc Dis 2013;23(9):816-21. doi: 10.1016/j.numecd.2012.08.004.
55. Lutsey PL, Steffen LM, Stevens J. Dietary intake and the development of the metabolic syndrome: the Atherosclerosis Risk in Communities study. Circulation 2008;117(6):754-61. doi: 10.1161/circulationaha.107.716159.
56. Malik VS, Sun Q, van Dam RM, Rimm EB, Willett WC, Rosner B, Hu FB. Adolescent dairy product consumption and risk of type 2 diabetes in middle-aged women. Am J Clin Nutr 2011;94(3):854-61. doi: 10.3945/ajcn.110.009621.
57. Mirmiran P, Moslehi N, Hosseinpanah F, Sarbazi N, Azizi F. Dietary determinants of unhealthy metabolic phenotype in normal weight and overweight/obese adults: results of a prospective study. Int J Food Sci Nutr 2020;71(7):891-901. doi: 10.1080/09637486.2020.1746955.
58. Montonen J, Knekt P, Härkänen T, Järvinen R, Heliövaara M, Aromaa A, Reunanen A. Dietary patterns and the incidence of type 2 diabetes. Am J Epidemiol 2005;161(3):219-27. doi: 10.1093/aje/kwi039.
59. Nettleton JA, Steffen LM, Ni H, Liu K, Jacobs DR, Jr. Dietary patterns and risk of incident type 2 diabetes in the Multi-Ethnic Study of Atherosclerosis (MESA). Diabetes Care 2008;31(9):1777-82. doi: 10.2337/dc08-0760.
60. O'Connor L, Imamura F, Lentjes MA, Khaw KT, Wareham NJ, Forouhi NG. Prospective associations and population impact of sweet beverage intake and type 2 diabetes, and effects of substitutions with alternative beverages. Diabetologia 2015;58(7):1474-83. doi: 10.1007/s00125-015-3572-1.
61. O'Connor LM, Lentjes MA, Luben RN, Khaw KT, Wareham NJ, Forouhi NG. Dietary dairy product intake and incident type 2 diabetes: a prospective study using dietary data from a 7-day food diary. Diabetologia 2014;57(5):909-17. doi: 10.1007/s00125-014-3176-1.
62. Oh JM, Woo HW, Kim MK, Lee YH, Shin DH, Shin MH, Choi BY. Dietary total, animal, vegetable calcium and type 2 diabetes incidence among Korean adults: The Korean Multi-Rural Communities Cohort (MRCohort). NUTRITION METABOLISM AND CARDIOVASCULAR DISEASES 2017;27(12):1152-64. doi: 10.1016/j.numecd.2017.10.005.
63. Osadnik K, Osadnik T, Lonnie M, Lejawa M, Reguła R, Fronczek M, Gawlita M, Wądołowska L, Gąsior M, Pawlas N. Metabolically healthy obese and metabolic syndrome of the lean: the importance of diet quality. Analysis of MAGNETIC cohort. Nutr J 2020;19(1):19. doi: 10.1186/s12937-020-00532-0.
64. Pastorino S, Richards M, Pierce M, Ambrosini GL. A high-fat, high-glycaemic index, low-fibre dietary pattern is prospectively associated with type 2 diabetes in a British birth cohort. Br J Nutr 2016;115(9):1632-42. doi: 10.1017/s0007114516000672.
65. Pereira MA, Jacobs DR, Jr., Van Horn L, Slattery ML, Kartashov AI, Ludwig DS. Dairy consumption, obesity, and the insulin resistance syndrome in young adults: the CARDIA Study. JAMA 2002;287(16):2081-9. doi: 10.1001/jama.287.16.2081.
66. Prada M, Wittenbecher C, Eichelmann F, Wernitz A, Drouin-Chartier JP, Schulze MB. Association of the odd-chain fatty acid content in lipid groups with type 2 diabetes risk: A targeted analysis of lipidomics data in the EPIC-Potsdam cohort. Clin Nutr 2021;40(8):4988-99. doi: 10.1016/j.clnu.2021.06.006.
67. Ribeiro AG, Mill JG, Matos SMA, Velasquez-Melendez G, Cade NV, Molina MDB. Associations between consumption of dairy products, C-reactive protein, and lipid profile in adults: results of the ELSA-Brasil study. CADERNOS DE SAUDE PUBLICA 2020;36(1). doi: 10.1590/0102-311X00028019.
68. Rosenberg L, Robles YP, Li S, Ruiz-Narvaez EA, Palmer JR. A prospective study of yogurt and other dairy consumption in relation to incidence of type 2 diabetes among black women in the USA. Am J Clin Nutr 2020;112(3):512-8. doi: 10.1093/ajcn/nqaa143.
69. Sarebanhassanabadi M, Mirhosseini SJ, Mirzaei M, Namay, eh SM, Soltani MH, Pakseresht M, Pedarzadeh A, Baramesipour Z, Faraji R, et al. Effect of dietary habits on the risk of metabolic syndrome: Yazd Healthy Heart Project. PUBLIC HEALTH NUTRITION 2018;21(6):1139-46. doi: 10.1017/S1368980017003627.
70. Satija A, Bhupathiraju SN, Rimm EB, Spiegelman D, Chiuve SE, Borgi L, Willett WC, Manson JE, Sun Q, Hu FB. Plant-Based Dietary Patterns and Incidence of Type 2 Diabetes in US Men and Women: Results from Three Prospective Cohort Studies. PLoS Med 2016;13(6):e1002039. doi: 10.1371/journal.pmed.1002039.
71. Sayón-Orea C, Bes-Rastrollo M, Martí A, Pimenta AM, Martín-Calvo N, Martínez-González MA. Association between yogurt consumption and the risk of metabolic syndrome over 6 years in the SUN study. BMC Public Health 2015;15:170. doi: 10.1186/s12889-015-1518-7.
72. Seah JYH, Ong CN, Koh WP, Yuan JM, van Dam RM. A Dietary Pattern Derived from Reduced Rank Regression and Fatty Acid Biomarkers Is Associated with Lower Risk of Type 2 Diabetes and Coronary Artery Disease in Chinese Adults. J Nutr 2019;149(11):2001-10. doi: 10.1093/jn/nxz164.
73. Shin H, Yoon YS, Lee Y, Kim CI, Oh SW. Dairy product intake is inversely associated with metabolic syndrome in Korean adults: Anseong and Ansan cohort of the Korean Genome and Epidemiology Study. J Korean Med Sci 2013;28(10):1482-8. doi: 10.3346/jkms.2013.28.10.1482.
74. Sluijs I, Forouhi NG, Beulens JW, van der Schouw YT, Agnoli C, Arriola L, Balkau B, Barricarte A, Boeing H, Bueno-de-Mesquita HB, et al. The amount and type of dairy product intake and incident type 2 diabetes: results from the EPIC-InterAct Study. Am J Clin Nutr 2012;96(2):382-90. doi: 10.3945/ajcn.111.021907.
75. Soedamah-Muthu SS, Masset G, Verberne L, Geleijnse JM, Brunner EJ. Consumption of dairy products and associations with incident diabetes, CHD and mortality in the Whitehall II study. Br J Nutr 2013;109(4):718-26. doi: 10.1017/s0007114512001845.
76. Sossa C, Delisle H, Agueh V, Sodjinou R, Nt, ou G, Makoutodé M. Lifestyle and dietary factors associated with the evolution of cardiometabolic risk over four years in West-African adults: the Benin study. J Obes 2013;2013:298024. doi: 10.1155/2013/298024.
77. Str, MA, Perry J, Wang P, Liu S, Lynn H. Risk factors for metabolic syndrome in a cohort study in a north China urban middle-aged population. Asia Pac J Public Health 2015;27(2):Np255-65. doi: 10.1177/1010539512438609.
78. Ushula TW, Mamun A, Darssan D, Wang WYS, Williams GM, Whiting SJ, Najman JM. Dietary patterns explaining variations in blood biomarkers in young adults are associated with the 30-year predicted cardiovascular disease risks in midlife: A follow-up study. Nutr Metab Cardiovasc Dis 2023;33(5):1007-18. doi: 10.1016/j.numecd.2023.02.019. Epub 2023 Feb 27.
79. van Dam RM, Hu FB, Rosenberg L, Krishnan S, Palmer JR. Dietary calcium and magnesium, major food sources, and risk of type 2 diabetes in U.S. black women. Diabetes Care 2006;29(10):2238-43. doi: 10.2337/dc06-1014.
80. van Dam RM, Rimm EB, Willett WC, Stampfer MJ, Hu FB. Dietary patterns and risk for type 2 diabetes mellitus in U.S. men. Ann Intern Med 2002;136(3):201-9. doi: 10.7326/0003-4819-136-3-200202050-00008.
81. van Nielen M, Feskens EJM, Mensink M, Sluijs I, Molina E, Amiano P, Ardanaz E, Balkau B, Beulens JWJ, Boeing H, et al. Dietary Protein Intake and Incidence of Type 2 Diabetes in Europe: The EPIC-InterAct Case-Cohort Study. DIABETES CARE 2014;37(7):1854-62. doi: 10.2337/dc13-2627.
82. Villegas R, Gao YT, Dai Q, Yang G, Cai H, Li H, Zheng W, Shu XO. Dietary calcium and magnesium intakes and the risk of type 2 diabetes: the Shanghai Women's Health Study. Am J Clin Nutr 2009;89(4):1059-67. doi: 10.3945/ajcn.2008.27182.
83. Villegas R, Yang G, Gao YT, Cai H, Li H, Zheng W, Shu XO. Dietary patterns are associated with lower incidence of type 2 diabetes in middle-aged women: the Shanghai Women's Health Study. Int J Epidemiol 2010;39(3):889-99. doi: 10.1093/ije/dyq008.
84. Virtanen HEK, Koskinen TT, Voutilainen S, Mursu J, Tuomainen TP, Kokko P, Virtanen JK. Intake of different dietary proteins and risk of type 2 diabetes in men: the Kuopio Ischaemic Heart Disease Risk Factor Study. Br J Nutr 2017;117(6):882-93. doi: 10.1017/S0007114517000745.
85. Vissers LET, Sluijs I, van der Schouw YT, Forouhi NG, Imamura F, Burgess S, Barricarte A, Boeing H, Bonet C, Chirlaque MD, et al. Dairy Product Intake and Risk of Type 2 Diabetes in EPIC-InterAct: A Mendelian Randomization Study. Diabetes Care 2019;42(4):568-75. doi: 10.2337/dc18-2034.
86. Vitezova A, Zillikens MC, van Herpt TTW, Sijbr, s EJG, Hofman A, Uitterlinden AG, Franco OH, Kiefte-de Jong JC. Vitamin D status and metabolic syndrome in the elderly: the Rotterdam Study. EUROPEAN JOURNAL OF ENDOCRINOLOGY 2015;172(3):327-35. doi: 10.1530/EJE-14-0580.
87. Wei L, Fan J, Dong R, Zhang M, Jiang Y, Zhao Q, Zhao G, Chen B, Li J, Liu S. The Effect of Dietary Pattern on Metabolic Syndrome in a Suburban Population in Shanghai, China. Nutrients 2023;15(9). doi: 10.3390/nu15092185.
88. Woo HW, Lim YH, Kim MK, Shin J, Lee YH, Shin DH, Shin MH, Choi BY. Prospective associations between total, animal, and vegetable calcium intake and metabolic syndrome in adults aged 40 years and older. CLINICAL NUTRITION 2020;39(7):2282-91. doi: 10.1016/j.clnu.2019.10.020.
89. Xiao X, Qin Z, Lv X, Dai Y, Ciren Z, Yangla Y, Zeng P, Ma Y, Li X, Wang L, et al. Dietary patterns and cardiometabolic risks in diverse less-developed ethnic minority regions: results from the China Multi-Ethnic Cohort (CMEC) Study. Lancet Reg Health West Pac 2021;15:100252. doi: 10.1016/j.lanwpc.2021.100252. eCollection 2021 Oct.
90. Yakoob MY, Shi P, Willett WC, Rexrode KM, Campos H, Orav EJ, Hu FB, Mozaffarian D. Circulating Biomarkers of Dairy Fat and Risk of Incident Diabetes Mellitus Among Men and Women in the United States in Two Large Prospective Cohorts. Circulation 2016;133(17):1645-54. doi: 10.1161/CIRCULATIONAHA.115.018410. Epub 2016 Mar 22.
91. Yogal C, Borgen M, Shakya S, Karmarcharya B, Koju R, Mosti MP, Gustafsson MK, Åsvold BO, Schei B, Stunes AK, et al. Vitamin D Status among Women in a Rural District of Nepal: Determinants and Association with Metabolic Profile-A Population-Based Study. Nutrients 2022;14(11). doi: 10.3390/nu14112309.
92. Yoko J, Nanri A, Eguchi M, Kochi T, Kabe I, Mizoue T. Total, low-fat, and full-fat dairy consumption and risk of metabolic syndrome among workers. Clin Nutr ESPEN 2021;46:350-5. doi: 10.1016/j.clnesp.2021.09.733.
93. Yu R, Woo J, Chan R, Sham A, Ho S, Tso A, Cheung B, Lam TH, Lam K. Relationship between dietary intake and the development of type 2 diabetes in a Chinese population: the Hong Kong Dietary Survey. Public Health Nutr 2011;14(7):1133-41. doi: 10.1017/S136898001100053X. Epub 2011 Apr 5.
94. Yuan MJ, Singer MR, Pickering RT, Moore LL. Saturated fat from dairy sources is associated with lower cardiometabolic risk in the Framingham Offspring Study. AMERICAN JOURNAL OF CLINICAL NUTRITION 2022;116(6):1682-92. doi: 10.1093/ajcn/nqac224.
95. Yuzbashian E, Pakseresht M, Vena J, Chan CB. Association of dairy consumption patterns with the incidence of type 2 diabetes: Findings from Alberta's Tomorrow Project. Nutr Metab Cardiovasc Dis 2022;32(12):2760-71. doi: 10.1016/j.numecd.2022.09.022.
96. Zhang J, Lim K, Shin S. Dairy product consumption and type 2 diabetes among Korean adults: a prospective cohort study based on the Health Examinees (HEXA) study. Epidemiol Health 2022;44:e2022019. doi: 10.4178/epih.e2022019.
97. Abiemo EE, Alonso A, Nettleton JA, Steffen LM, Bertoni AG, Jain A, Lutsey PL. Relationships of the Mediterranean dietary pattern with insulin resistance and diabetes incidence in the Multi-Ethnic Study of Atherosclerosis (MESA). Br J Nutr 2013;109(8):1490-7. doi: 10.1017/s0007114512003339.
98. Anderson AL, Harris TB, Tylavsky FA, Perry SE, Houston DK, Lee JS, Kanaya AM, Sahyoun NR. Dietary patterns, insulin sensitivity and inflammation in older adults. Eur J Clin Nutr 2012;66(1):18-24. doi: 10.1038/ejcn.2011.162. Epub 2011 Sep 14.
99. Batis C, Mendez MA, Sotres-Alvarez D, Gordon-Larsen P, Popkin B. Dietary pattern trajectories during 15 years of follow-up and HbA1c, insulin resistance and diabetes prevalence among Chinese adults. J Epidemiol Community Health 2014;68(8):773-9. doi: 10.1136/jech-2013-203560. Epub 2014 Apr 12.
100. Chen Z, Franco OH, Lamballais S, Ikram MA, Schoufour JD, Muka T, Voortman T. Associations of specific dietary protein with longitudinal insulin resistance, prediabetes and type 2 diabetes: The Rotterdam Study. Clin Nutr 2020;39(1):242-9. doi: 10.1016/j.clnu.2019.01.021.
101. Czekajło A, Różańska D, Zatońska K, Szuba A, Regulska-Ilow B. Association between dietary patterns and cardiovascular risk factors in a selected population of Lower Silesia (PURE Study Poland). Ann Agric Environ Med 2018;25(4):635-41. doi: 10.26444/aaem/76321.
102. Doostv, i T, Bahadoran Z, Mozaffari-Khosravi H, Tahmasebinejad Z, Mirmiran P, Azizi F. The association of dietary patterns and the incidence of insulin resistance after a 3-year follow-up: Tehran Lipid and Glucose Study. Asia Pac J Clin Nutr 2017;26(3):531-8. doi: 10.6133/apjcn.032016.12.
103. Drake I, Sonestedt E, Ericson U, Wallström P, Orho M, er M. A Western dietary pattern is prospectively associated with cardio-metabolic traits and incidence of the metabolic syndrome. Br J Nutr 2018;119(10):1168-76. doi: 10.1017/S000711451800079X.
104. Drouillet P, Balkau B, Charles MA, Vol S, Bedouet M, Ducimetière P. Calcium consumption and insulin resistance syndrome parameters. Data from the Epidemiological Study on the Insulin Resistance Syndrome (DESIR). Nutr Metab Cardiovasc Dis 2007;17(7):486-92. doi: 10.1016/j.numecd.2007.01.006. Epub 2007 Jul 6.
105. Ericson U, Brunkwall L, Alves Dias J, Drake I, Hellstr, S., Gullberg B, Sonestedt E, Nilsson PM, Wirfält E, et al. Food patterns in relation to weight change and incidence of type 2 diabetes, coronary events and stroke in the Malmö Diet and Cancer cohort. Eur J Nutr 2019;58(5):1801-14. doi: 10.1007/s00394-018-1727-9. Epub 2018 May 31.
106. Forouhi NG, Koulman A, Sharp SJ, Imamura F, Kroger J, Schulze MB, Crowe FL, Huerta JM, Guevara M, Beulens JWJ, et al. Differences in the prospective association between individual plasma phospholipid saturated fatty acids and incident type 2 diabetes: the EPIC-InterAct case-cohort study. LANCET DIABETES & ENDOCRINOLOGY 2014;2(10):810-8. doi: 10.1016/S2213-8587(14)70146-9.
107. Fung TT, Rimm EB, Spiegelman D, Rifai N, Tofler GH, Willett WC, Hu FB. Association between dietary patterns and plasma biomarkers of obesity and cardiovascular disease risk. Am J Clin Nutr 2001;73(1):61-7. doi: 10.1093/ajcn/73.1.61.
108. Gagnon C, Lu ZX, Magliano DJ, Dunstan DW, Shaw JE, Zimmet PZ, Sikaris K, Grantham N, Ebeling PR, Daly RM. Serum 25-Hydroxyvitamin D, Calcium Intake, and Risk of Type 2 Diabetes After 5 Years Results from a national, population-based prospective study (the Australian Diabetes, Obesity and Lifestyle study). DIABETES CARE 2011;34(5):1133-8. doi: 10.2337/dc10-2167.
109. Günther AL, Schulze MB, Kroke A, Diethelm K, Joslowski G, Krupp D, Wudy S, Buyken AE. Early Diet and Later Cancer Risk: Prospective Associations of Dietary Patterns During Critical Periods of Childhood with the GH-IGF Axis, Insulin Resistance and Body Fatness in Younger Adulthood. Nutr Cancer 2015;67(6):877-92. doi: 10.1080/01635581.2015.1056313. Epub 2015 Jul 30.
110. Hu MJ, Tan JS, Gao XJ, Yang JG, Yang YJ. Effect of Cheese Intake on Cardiovascular Diseases and Cardiovascular Biomarkers. Nutrients 2022;14(14). doi: 10.3390/nu14142936.
111. Imamura F, Schulze MB, Sharp SJ, Guevara M, Romaguera D, Bendinelli B, Salamanca-Fernández E, Ardanaz E, Arriola L, Aune D, et al. Estimated Substitution of Tea or Coffee for Sugar-Sweetened Beverages Was Associated with Lower Type 2 Diabetes Incidence in Case-Cohort Analysis across 8 European Countries in the EPIC-InterAct Study. J Nutr 2019;149(11):1985-93. doi: 10.1093/jn/nxz156.
112. Ivey KL, Nguyen XT, Posner D, Rogers GB, Tobias DK, Song R, Ho YL, Li R, Wilson PWF, Cho K, et al. The Structure of Relationships between the Human Exposome and Cardiometabolic Health: The Million Veteran Program. Nutrients 2021;13(4). doi: 10.3390/nu13041364.
113. Jacobo Cejudo MG, Ochoa-Rosales C, Ahmadizar F, Kavousi M, Geleijnse JM, Voortman T. The healthy beverage index is not associated with insulin resistance, prediabetes and type 2 diabetes risk in the Rotterdam Study. Eur J Nutr 2023;62(7):3021-31. doi: 10.1007/s00394-023-03209-6. Epub 2023 Jul 25.
114. Jacobs S, Kroeger J, Schulze MB, Frank LK, Franke AA, Cheng I, Monroe KR, Haiman CA, Kolonel LN, Wilkens LR, et al. Dietary Patterns Derived by Reduced Rank Regression Are Inversely Associated with Type 2 Diabetes Risk across 5 Ethnic Groups in the Multiethnic Cohort. Curr Dev Nutr 2017;1(5):e000620. doi: 10.3945/cdn.117.000620. eCollection 2017 May.
115. Kerver JM, Eun JY, Bianchi L, Song WO. Dietary patterns associated with risk factors for cardiovascular disease in healthy US adults. American Journal of Clinical Nutrition 2003;78(6):1103-10. doi: 10.1093/ajcn/78.6.1103.
116. Laaksonen DE, Lakka TA, Lakka HM, Nyyssönen K, Rissanen T, Niskanen LK, Salonen JT. Serum fatty acid composition predicts development of impaired fasting glycaemia and diabetes in middle-aged men. Diabet Med 2002;19(6):456-64. doi: 10.1046/j.1464-5491.2002.00707.x.
117. Lee DH, Tabung FK, Giovannucci EL. Association of animal and plant protein intakes with biomarkers of insulin and insulin-like growth factor axis. Clinical Nutrition 2022;41(6):1272-80. doi: 10.1016/j.clnu.2022.04.003.
118. Maldonado LE, Sotres-Alvarez D, Mattei J, Daviglus ML, Talavera GA, Perreira KM, Van Horn L, Mossavar-Rahmani Y, LeCroy MN, Gallo LC, et al. A Posteriori dietary patterns, insulin resistance, and diabetes risk by Hispanic/Latino heritage in the HCHS/SOL cohort. Nutr Diabetes 2022;12(1):44. doi: 10.1038/s41387-022-00221-3.
119. Mattei J, Sotos-Prieto M, Bigornia SJ, Noel SE, Tucker KL. The Mediterranean Diet Score Is More Strongly Associated with Favorable Cardiometabolic Risk Factors over 2 Years Than Other Diet Quality Indexes in Puerto Rican Adults. J Nutr 2017;147(4):661-9. doi: 10.3945/jn.116.245431. Epub 2017 Mar 8.
120. McNaughton SA, Mishra GD, Stephen AM, Wadsworth ME. Dietary patterns throughout adult life are associated with body mass index, waist circumference, blood pressure, and red cell folate. J Nutr 2007;137(1):99-105. doi: 10.1093/jn/137.1.99.
121. Mozaffarian D, de Oliveira Otto MC, Lemaitre RN, Fretts AM, Hotamisligil G, Tsai MY, Siscovick DS, Nettleton JA. trans-Palmitoleic acid, other dairy fat biomarkers, and incident diabetes: the Multi-Ethnic Study of Atherosclerosis (MESA). Am J Clin Nutr 2013;97(4):854-61. doi: 10.3945/ajcn.112.045468.
122. Richart T, Thijs L, Nawrot T, Yu J, Kuznetsova T, Balkestein EJ, Struijker-Boudier HA, Staessen JA. The metabolic syndrome and carotid intima-media thickness in relation to the parathyroid hormone to 25-OH-D(3) ratio in a general population. Am J Hypertens 2011;24(1):102-9. doi: 10.1038/ajh.2010.124. Epub 2010 Jul 1.
123. Santaren ID, Watkins SM, Liese AD, Wagenknecht LE, Rewers MJ, Haffner SM, Lorenzo C, Hanley AJ. Serum pentadecanoic acid (15:0), a short-term marker of dairy food intake, is inversely associated with incident type 2 diabetes and its underlying disorders. Am J Clin Nutr 2014;100(6):1532-40. doi: 10.3945/ajcn.114.092544. Epub 2014 Oct 8.
124. Santiago-Torres M, Tinker LF, Allison MA, Breymeyer KL, Garcia L, Kroenke CH, Lampe JW, Shikany JM, Van Horn L, Neuhouser ML. Development and Use of a Traditional Mexican Diet Score in Relation to Systemic Inflammation and Insulin Resistance among Women of Mexican Descent. J Nutr 2015;145(12):2732-40. doi: 10.3945/jn.115.213538. Epub 2015 Oct 21.
125. Sarebanhassanabadi M, Mirhosseini SJ, Mirzaei M, Namay, eh SM, Soltani MH, Pedarzadeh A, Baramesipour Z, Faraji R, Ahmadi N, et al. Association between dietary habits and changes in cardiometabolic risk factors in patients with metabolic syndrome: a 10-year follow-up study. PROGRESS IN NUTRITION 2019;21:348-58. doi: 10.23751/pn.v21i1-S.6493.
126. Shin D, Song SJ, Krumhar K, Song WO. Snack patterns are associated with biomarkers of glucose metabolism in US men. INTERNATIONAL JOURNAL OF FOOD SCIENCES AND NUTRITION 2015;66(5):595-602. doi: 10.3109/09637486.2015.1064873.
127. Sun J, Buys N, Shen S. Dietary patterns and cardiovascular disease-related risks in chinese older adults. Front Public Health 2013;1:48. doi: 10.3389/fpubh.2013.00048. eCollection 2013.
128. Szypowska A, Regulska-Ilow B, Zatońska K, Szuba A. Comparison of Intake of Food Groups Based on Dietary Inflammatory Index (DII) and Cardiovascular Risk Factors in the Middle-Age Population of Lower Silesia: Results of the PURE Poland Study. Antioxidants (Basel) 2023;12(2). doi: 10.3390/antiox12020285.
129. van Nielen M, Feskens EJM, Mensink M, Sluijs I, Molina E, Amiano P, Ardanaz E, Balkau B, Beulens JWJ, Boeing H, et al. Dietary Protein Intake and Incidence of Type 2 Diabetes in Europe: The EPIC-InterAct Case-Cohort Study. DIABETES CARE 2014;37(7):1854-62. doi: 10.2337/dc13-2627.
130. Wu F, Pahkala K, Juonala M, Rovio SP, Sabin MA, Rönnemaa T, Buscot MJ, Smith KJ, Männistö S, Jula A, et al. Dietary Pattern Trajectories from Youth to Adulthood and Adult Risk of Impaired Fasting Glucose: A 31-year Cohort Study. J Clin Endocrinol Metab 2021;106(5):e2078-e86. doi: 10.1210/clinem/dgab044.
131. Yehia NA, Isai L, Semnani-Azad Z, Lai KZH, Retnakaran R, Harris SB, Beaudry JL, Bazinet RP, Hanley AJ. Association of circulating branched chain fatty acids with insulin sensitivity and beta cell function in the PROMISE cohort. Lipids 2023;58(4):171-83. doi: 10.1002/lipd.12373. Epub 2023 May 11.
132. Azadbakht L, Mirmiran P, Esmaillzadeh A, Azizi F. Dairy consumption is inversely associated with the prevalence of the metabolic syndrome in Tehranian adults. American Journal of Clinical Nutrition 2005;82(3):523-30. doi: 10.1093/ajcn.82.3.523.
133. Bergholdt HK, Nordestgaard BG, Varbo A, Ellervik C. Milk intake is not associated with ischaemic heart disease in observational or Mendelian randomization analyses in 98,529 Danish adults. Int J Epidemiol 2015;44(2):587-603. doi: 10.1093/ije/dyv109.
134. Charatcharoenwitthaya P, Tansakul E, Chaiyasoot K, idniyamanon W, Charatcharoenwitthaya N. Dietary composition and its association with newly diagnosed nonalcoholic fatty liver disease and insulin resistance. Nutrients 2021;13(12). doi: 10.3390/nu13124438.
135. Crichton GE, Alkerwi A. Dairy food intake is positively associated with cardiovascular health: findings from Observation of Cardiovascular Risk Factors in Luxembourg study. NUTRITION RESEARCH 2014;34(12):1036-44. doi: 10.1016/j.nutres.2014.04.002.
136. Crichton GE, Bogucki OE, Elias MF. Dairy food intake, diet patterns, and health: Findings from the Maine-Syracuse Longitudinal Study. Int Dairy J 2019;91:64-70. doi: 10.1016/j.idairyj.2018.12.009.
137. Drehmer M, Pereira MA, Schmidt MI, Del Carmen BMM, Alvim S, Lotufo PA, Duncan BB. Associations of dairy intake with glycemia and insulinemia, independent of obesity, in Brazilian adults: the Brazilian Longitudinal Study of Adult Health (ELSA-Brasil). Am J Clin Nutr 2015;101(4):775-82. doi: 10.3945/ajcn.114.102152.
138. Lawlor DA, Ebrahim S, Timpson N, Smith GD. Avoiding milk is associated with a reduced risk of insulin resistance and the metabolic syndrome: Findings from the British Women's Heart and Health Study. Diabetic Medicine 2005;22(6):808-11. doi: 10.1111/j.1464-5491.2005.01537.x.
139. Mozaffarian D, Cao H, King IB, Lemaitre RN, Song X, Siscovick DS, Hotamisligil GS. Trans-palmitoleic acid, metabolic risk factors, and new-onset diabetes in U.S. adults: a cohort study. Ann Intern Med 2010;153(12):790-9. doi: 10.7326/0003-4819-153-12-201012210-00005.
140. Wang H, Livingston KA, Fox CS, Meigs JB, Jacques PF. Yogurt consumption is associated with better diet quality and metabolic profile in American men and women. Nutrition Research 2013;33(1):18-26. doi: 10.1016/j.nutres.2012.11.009.
141. Westerman KE, Miao J, Chasman DI, Florez JC, Chen H, Manning AK, Cole JB. Genome-wide gene-diet interaction analysis in the UK Biobank identifies novel effects on hemoglobin A1c. Hum Mol Genet 2021;30(18):1773-83. doi: 10.1093/hmg/ddab109.
142. Zhu R, Fogelholm M, Jalo E, Poppitt SD, Silvestre MP, Møller G, Huttunen-Lenz M, Stratton G, Sundvall J, Macdonald IA, et al. Animal-based food choice and associations with long-term weight maintenance and metabolic health after a large and rapid weight loss: The PREVIEW study. Clin Nutr 2022;41(4):817-28. doi: 10.1016/j.clnu.2022.02.002.
143. Netto BD, Earthman CP, Farias G, Masquio DCL, Clemente APG, Peixoto P, Bettini SC, von Der Heyde ME, Damaso AR. Eating patterns and food choice as determinant of weight loss and improvement of metabolic profile after RYGB. NUTRITION 2017;33:125-31. doi: 10.1016/j.nut.2016.05.007.
144. Sarebanhassanabadi M, Mirhosseini SJ, Mirzaei M, Namayandeh SM, Soltani MH, Pedarzadeh A, Baramesipour Z, Faraji R, Ahmadi N, Salehi-Abargouei A. Association between dietary habits and changes in cardiometabolic risk factors in patients with metabolic syndrome: a 10-year follow-up study. PROGRESS IN NUTRITION 2019;21:348-58. doi: 10.23751/pn.v21i1-S.6493.
145. Lecomte P, Cacès E, Lasfargues G, Combe H, Laurent S, Tichet J. Impaired fasting glycaemia and undiagnosed diabetes: prevalence, cardiovascular and behavioural risk factors. Diabetes & metabolism 2002;28(4 Pt 1):311-20.
146. Hruby A, Ma J, Rogers G, Meigs JB, Jacques PF. Associations of Dairy Intake with Incident Prediabetes or Diabetes in Middle-Aged Adults Vary by Both Dairy Type and Glycemic Status. J Nutr 2017;147(9):1764-75. doi: 10.3945/jn.117.253401.
147. Slurink IAL, den Braver NR, Rutters F, Kupper N, Smeets T, Elders PJM, Beulens JWJ, Soedamah-Muthu SS. Dairy product consumption and incident prediabetes in Dutch middle-aged adults: the Hoorn Studies prospective cohort. Eur J Nutr 2022;61(1):183-96. doi: 10.1007/s00394-021-02626-9.
148. Slurink IA, Chen L, Magliano DJ, Kupper N, Smeets T, Soedamah-Muthu SS. Dairy Product Consumption and Incident Prediabetes in the Australian Diabetes, Obesity, and Lifestyle Study With 12 Years of Follow-Up. J Nutr 2023;153(6):1742-52. doi: 10.1016/j.tjnut.2023.03.032.
149. Slurink IAL, Voortman T, Ochoa-Rosales C, Ahmadizar F, Kavousi M, Kupper N, Smeets T, Soedamah-Muthu SS. Dairy Product Consumption in Relation to Incident Prediabetes and Longitudinal Insulin Resistance in the Rotterdam Study. Nutrients 2022;14(3). doi: 10.3390/nu14030415.
150. Slurink IA, Corpeleijn E, Bakker SJ, Jongerling J, Kupper N, Smeets T, Soedamah-Muthu SS. Dairy consumption and incident prediabetes: prospective associations and network models in the large population-based Lifelines Study. Am J Clin Nutr 2023;118(6):1077-90. doi: 10.1016/j.ajcnut.2023.10.002.
151. Slurink I, Kupper N, Smeets T, Soedamah-Muthu S. Dairy consumption and risk of prediabetes and type 2 diabetes in the Fenland Study [Manuscript submitted for publication].
152. Feldman AL, Long GH, Johansson I, Weinehall L, Fhärm E, Wennberg P, Norberg M, Griffin SJ, Rolandsson O. Change in lifestyle behaviors and diabetes risk: evidence from a population-based cohort study with 10 year follow-up. International Journal of behavioral nutrition and physical activity 2017;14:1-10.
153. VanWormer JJ, Boucher JL, Sidebottom AC, Sillah A, Knickelbine T. Lifestyle changes and prevention of metabolic syndrome in the Heart of New Ulm Project. Preventive medicine reports 2017;6:242-5.
154. Higgins JP, Morgan RL, Rooney AA, Taylor KW, Thayer KA, Silva RA, Lemeris C, Akl EA, Bateson TF, Berkman ND. A tool to assess risk of bias in non-randomized follow-up studies of exposure effects (ROBINS-E). Environment international 2024;186:108602.
155. Kiesswetter E, Neuenschwander M, Stadelmaier J, Szczerba E, Hofacker L, Sedlmaier K, Kussmann M, Roeger C, Hauner H, Schlesinger S. Substitution of dairy products and risk of death and cardiometabolic diseases: a systematic review and meta-analysis of prospective studies. Current Developments in Nutrition 2024:102159.
156. Feskens EJ, Virtanen SM, Rasanen L, Tuomilehto J, Stengard J, Pekkanen J, Nissinen A, Kromhout D. Dietary factors determining diabetes and impaired glucose tolerance. A 20-year follow-up of the Finnish and Dutch cohorts of the Seven Countries Study. Diabetes Care 1995;18(8):1104-12. doi: 10.2337/diacare.18.8.1104.
157. Ma B, Lawson AB, Liese AD, Bell RA, Mayer-Davis EJ. Dairy, magnesium, and calcium intake in relation to insulin sensitivity: approaches to modeling a dose-dependent association. Am J Epidemiol 2006;164(5):449-58. doi: 10.1093/aje/kwj246.
158. Snijder MB, van Dam RM, Stehouwer CD, Hiddink GJ, Heine RJ, Dekker JM. A prospective study of dairy consumption in relation to changes in metabolic risk factors: the Hoorn Study. Obesity (Silver Spring) 2008;16(3):706-9. doi: 10.1038/oby.2007.93.
159. Struijk EA, Heraclides A, Witte DR, Soedamah-Muthu SS, Geleijnse JM, Toft U, Lau CJ. Dairy product intake in relation to glucose regulation indices and risk of type 2 diabetes. Nutr Metab Cardiovasc Dis 2013;23(9):822-8. doi: 10.1016/j.numecd.2012.05.011.
160. Samara A, Herbeth B, Ndiaye NC, Fumeron F, Billod S, Siest G, Visvikis-Siest S. Dairy product consumption, calcium intakes, and metabolic syndrome-related factors over 5 years in the STANISLAS study. Nutrition 2013;29(3):519-24. doi: 10.1016/j.nut.2012.08.013.
161. Panahi S, Doyon CY, Despres JP, Perusse L, Vohl MC, Drapeau V, Tremblay A. Yogurt consumption, body composition, and metabolic health in the Quebec Family Study. Eur J Nutr 2018;57(4):1591-603. doi: 10.1007/s00394-017-1444-9.
162. Huang L, Lin JS, Aris IM, Yang G, Chen WQ, Li LJ. Circulating Saturated Fatty Acids and Incident Type 2 Diabetes: A Systematic Review and Meta-Analysis. Nutrients 2019;11(5):998. doi: 10.3390/nu11050998.
163. Trichia E, Luben R, Khaw KT, Wareham NJ, Imamura F, Forouhi NG. The associations of longitudinal changes in consumption of total and types of dairy products and markers of metabolic risk and adiposity: findings from the European Investigation into Cancer and Nutrition (EPIC)-Norfolk study, United Kingdom. Am J Clin Nutr 2020;111(5):1018-26. doi: 10.1093/ajcn/nqz335.
164. Riseberg E, Lopez-Cepero A, Mangano KM, Tucker KL, Mattei J. Specific Dietary Protein Sources Are Associated with Cardiometabolic Risk Factors in the Boston Puerto Rican Health Study. J Acad Nutr Diet 2022;122(2):298-308 e3. doi: 10.1016/j.jand.2021.05.020.
165. Yun H, Sun L, Wu Q, Luo Y, Qi Q, Li H, Gu W, Wang J, Ning G, Zeng R. Lipidomic signatures of dairy consumption and associated changes in blood pressure and other cardiovascular risk factors among chinese adults. Hypertension 2022;79(8):1617-28.
166. Chatzidiakou Y, Jackson K, Givens D, Lovegrove J. Relationship between the consumption of dairy foods and markers of glycaemic control: evidence from the Caerphilly prospective cohort study. Proceedings of the Nutrition Society 2023;82(OCE1):E6.
